# Extracellular vesicle-mediated approaches for the diagnosis and therapy of MASLD: current advances and future prospective

**DOI:** 10.1186/s12944-024-02396-3

**Published:** 2025-01-07

**Authors:** Swasthika Gurjar, Ramanarayana Bhat A, Raghavendra Upadhya, Revathi P. Shenoy

**Affiliations:** 1 Department of Biochemistry, Kasturba Medical College, Manipal, Manipal Academy of Higher Education, Karnataka, 576104 Manipal India; 2 Manipal Centre for Biotherapeutics Research, Manipal, Manipal Academy of Higher Education, Karnataka, 576104 Manipal India

**Keywords:** Metabolic dysfunction-associated steatotic liver disease, Liver diseases, Extracellular vesicles, Biomarker, Targeted therapy

## Abstract

Metabolic dysfunction-associated steatotic liver disease (MASLD) is an asymptomatic, multifaceted condition often associated with various risk factors, including fatigue, obesity, insulin resistance, metabolic syndrome, and sleep apnea. The increasing burden of MASLD underscores the critical need for early diagnosis and effective therapies. Owing to the lack of efficient therapies for MASLD, early diagnosis is crucial. Consequently, noninvasive biomarkers and imaging techniques are essential for analyzing disease risk and play a pivotal role in the global diagnostic process. The use of extracellular vesicles has emerged as promising for early diagnosis and therapy of various liver ailments. Herein, a comprehensive summary of the current diagnostic modalities for MASLD is presented, highlighting their advantages and limitations while exploring the potential of extracellular vesicles (EVs) as innovative diagnostic and therapeutic tools for MASLD. With this aim, this review emphasizes an in-depth understanding of the origin of EVs and the pathophysiological alterations of these ectosomes and exosomes in various liver diseases. This review also explores the therapeutic potential of EVs as key components in the future management of liver disease. The dual role of EVs as biomarkers and their therapeutic utility in MASLD essentially highlights their clinical integration to improve MASLD diagnosis and treatment. While EV-based therapies are still in their early stages of development and require substantial research to increase their therapeutic value before they can be used clinically, the diagnostic application of EVs has been extensively explored. Moving forward, developing diagnostic devices leveraging EVs will be crucial in advancing MASLD diagnosis. Thus, the literature summarized provides suitable grounds for clinicians and researchers to explore EVs for devising diagnostic and treatment strategies for MASLD.

**Supplementary Information:**

The online version contains supplementary material available at 10.1186/s12944-024-02396-3.

## Introduction

Lifestyle is known to determine an individual’s quality of life by influencing various factors, including physical and mental well-being. Metabolic dysfunction-associated steatotic liver disease (MASLD) is a hepatic pathology in developed countries that affects approximately one-fourth of the population at the global level. In the rapidly growing era of the urban lifestyle, the adoption of a sedentary lifestyle accompanied by unhealthy dietary patterns has significantly contributed to health-related diseases, especially noncommunicable diseases [1, 2], such as obesity [3], diabetes mellitus [4], hypertension [5], dyslipidemia [6], cardiovascular diseases [7], metabolic syndrome [8], and musculoskeletal diseases [9]. Furthermore, it has also been shown to affect quality of sleep [10], life expectancy [11], productivity [12], and social interaction [13].

The liver, which is the metabolic center of the human body, is strongly affected by the combined effects of an inappropriate diet and a state of physical inactivity. It has been reported that overnutrition causes an energy imbalance in metabolic processes and the accumulation of fatty acids in the liver. In addition, reduced fatty oxidation in the liver as a result of physical inactivity exacerbates liver health [14, 15]. MASLD is one such outcome of hepatic pathology [16] and was previously known as nonalcoholic fatty liver disease.

MASLD is a liver pathology characterized by the sequential progression from simple benign to more severe forms and mainly consists of simple steatosis or metabolic dysfunction associated with the steatotic liver and metabolic dysfunction associated with steatohepatitis. MASLD is associated with additional complications, such as fibrosis, cirrhosis, and end-stage hepatocellular carcinoma (HCC) [17, 18]. It is a condition of hepatic steatosis that is primarily associated with cardiometabolic risk factors such as obesity, insulin resistance, dyslipidemia, and hypertension, with the exclusion of other identifiable causes [19].

According to a systematic review conducted in 2022, the worldwide prevalence of MASLD (NAFLD) is 32.4%, with an increase from 25.5% between 1990 and 2022. Although its prevalence has increased in women, it is lower than that in men [20]. In terms of mortality, there is a 1.93-fold greater risk of death in the MASLD population than in the general population, according to a study conducted in 2020 [21].

The pathology of this disease during the initial stage manifests as a silent killer with no potential symptoms but later results in severe health complications in the absence of an early diagnosis [22]. In such circumstances, histopathological analysis represents the sole diagnostic method for most hepatic diseases, and MASLD follows suit without exception. The conventional diagnostic method for MASLD involves a cell-penetrating method of reaping the tissue sample, which is often associated with the risk of death [23]. Even though biochemical parameters can elucidate some of the metabolic variations associated with hepato-pathophysiology, understanding the hepatic ailment by employing these parameters alone would be misleading, resulting in misinterpretations. Despite these noninvasive techniques, there is no single biomarker that accurately assists in diagnosis and staging, whereas imaging techniques lack sensitivity in detecting disease progression [24, 25]. Early diagnosis remains the only effective strategy for addressing the disease before it progresses too far.

Liquid biopsy is an emerging convenient alternative way of diagnosing and monitoring molecular changes associated with various diseases, including cancer [26, 27]. This minimally invasive technology simplifies the task of sampling and reduces the risk associated with diagnosis. Presently, the technique is widely utilized in cancer screening, particularly for screening genetic aberrations originating from any combination of components such as extracellular vesicles (EVs) [28–30], circulating tumor cells [31], and cell-free DNA [32]. These circulating components with greater accessibility can be utilized in real-time monitoring of disease progression, enabling the selection of appropriate personalized therapy [33].

EVs present in circulating blood can provide valuable information about the physiological status of parent cells [34, 35]. EVs are tiny, heterogeneously sized entities delimited by a lipid bilayer. These tiny vesicles, irrespective of their size, carry a variety of molecular cargos. They are incapable of replicating themselves, compelling these particles to obtain information about the parent cells [36]. EVs are primarily engaged in cellular communications. Additionally, they can influence cell signaling cascades, leading to the activation of multiple pathways, thereby participating in both normal physiological and pathophysiological functions [37]. EVs are a heterogeneous class of particles encompassing ectosomes produced by outward budding, whereas plasma membrane fusion of parent cells and exosomes originate in the endosomal network, which is released upon fusion of the multivesicular body to the plasma membrane, and apoptotic bodies, which are released as blebs of cells undergoing apoptosis [36, 38, 39].

Many molecular cargos, such as proteins, nucleic acids, and lipids, are enclosed within them, which can attain signature confirmation upon reaching certain physiological states or disease stages [40–47]. Even highly fragile molecules such as RNA can remain intact and protected from degradation by RNases when they are sorted and packed carefully within EVs [48]. Multiple studies have experimentally curated EVs as a source of biomarkers for liver-related diseases. EVs have been extensively explored in the past and continue to be investigated for the treatment of a variety of liver diseases, including MASLD [49], alcoholic fatty liver disease (AFLD) [50], drug-induced liver injury (DILI) [51], autoimmune hepatitis (AIH) [52], HCC [53], and viral hepatitis [54].

## Review aim

The aim of this study was to explore and summarize recent advancements in the diagnosis and treatment of MASLD, with a particular focus on the role of EVs in clinical applications. This review expounds on commonly employed diagnostic biomarkers in clinical settings for MASLD diagnosis. Additionally, this study highlights recent discoveries of blood-based biomarkers with promising diagnostic potential for MASLD. With respect to EV-centered approaches, this review aims to understand the effective utilization of these cellular vehicles in MASLD diagnosis and therapy, covering current advances and prospects.

In line with this aim, the relevant literature was selected primarily from the PubMed database, with a focus on clinically significant data. The publications from 1999 to November 2024, with a particular emphasis on research from the past decade, were included. This approach aligns with trends shown in Fig 2., which demonstrate a substantial rise in studies related to MASLD in the last ten years, reflecting the growing public health focus on its diagnosis and treatment. Similarly, research on EVs has expanded rapidly over the same period, especially in the fields of diagnostics and therapy. The overlap in these research timelines provided a strong rationale for concentrating literature selection within this timeframe.

### Extracellular vesicles

EVs, which were previously disregarded as cellular debris [55], are tiny, heterogeneous classes of naturally occurring nanoparticles delimited by the plasma membrane. These tiny particles lack the ability to self-replicate and are produced by parental cells to the extracellular spaces to perform a plethora of physiological functions [56]. The classification of EVs is still debatable; however, on the basis of the biogenetic pathway, EVs can be either ectosomes or exosomes [57]. Ectosomes are formed by the outward membrane blebbing of cells, whereas exosomes are formed by the inward blebbing of the endosomal membrane followed by the formation of multivesicular bodies, which release exosomes by exocytosis upon fusion with the plasma membrane [36, 58]. The biogenetic mechanisms of ectosomes and exosomes are intricate complex cellular mechanisms involving sorting mechanisms that specifically load specific molecular cargo, such as RNA, lipids, and proteins, into vesicles, making them distinct from parental cells [59]. Interestingly, exosome biogenesis is eukaryote specific, as it requires endosomes, whereas ectosomes are produced by both prokaryotic and eukaryotic cells [60, 61].

### Biogenesis of circulating EVs

#### Biogenesis of ectosomes

Ectosomes were first described as subcellular particles derived from platelets in normal serum and plasma, and they were often termed “platelet dust” [55]. Later, ectocytosis was described using stimulated neutrophils. Several studies on ectocytosis termed them shedding bodies or shedding particles and oncosomes to determine their functions and roles in cellular communication. They are produced by a biogenetic process that involves the vertical transfer of molecular cargo to the plasma membrane, which is subsequently packed in lipid bilayer particles via a distinct pathway. Parent cells utilize a distinct contractile machinery that enables cells to pinch off these vesicles at the cell surface [62].

The complexity of this biogenetic pathway is intermediate and is neither as complicated as the biogenesis of exosomes nor as simple as the production of apoptotic bodies due to indiscriminate plasma membrane blebbing [63].

Ectosomes originate from membrane blebbing, which is usually associated with specific changes in the lipid and protein components at specific sites of the plasma membrane altering its properties, such as its rigidity and curvature [64]. The formation of ectosomes is achieved through the dynamic interplay of phospholipid redistribution and the contraction of cytoskeletal proteins [60].

A diverse range of eukaryotic cells produce ectosomes under normal physiological conditions as well as during disease conditions. Under disease conditions, the highly regulated biogenetic pathway can undergo abrupt changes, leading to the aberrant shedding of ectosomes [64]. The biogenetic pathway and the factors influencing the biogenesis of ectosomes under normal physiological conditions and under altered physiological conditions are summarized below.

#### Mechanism of ectosomes biogenesis under normal physiological conditions

##### Changes in lipid composition

The structural properties and shapes of lipids depend upon their hydrophilic head groups, hydrophobic acyl chain length, and saturation. The compositions of the inner and outer leaflets of the plasma membrane are distinct from each other; the inner leaflet predominantly harbors amino phospholipids such as phosphatidyl serine (PS) and the external leaflet is enriched with sphingomyelin and phosphatidylcholine. In general, vesicle formation is associated with a change in lipid composition assisted by PS and the local recruitment of lipid-modifying enzymes such as aminophospholipid translocases, flippases, floppases, gelsolin, scramblases, and calpains [61, 65]. The lipid composition is strongly influenced by these enzymes, in which flippases translocate specifically PS into the inner leaflet and floppases translocate lipids outward. However, the enzyme scramblase promotes the unspecific bidirectional distribution of lipids across the plasma membrane [65–68].

Membrane asymmetry collapses during ectosome biogenesis, with an increase in the cytosolic Ca^2+^ concentration activating floppases and scramblases while simultaneously inhibiting flippases. The biodistribution of PS induces signals that release ectosomes. The induction of the budding/vesicle formation signal occurs due to surface exposure of phosphatidylserine, wherein the translocation of PS occurs from the inner leaflet to the outer leaflet of the plasma membrane [69].

#### Activation of the contractile machinery

The formation of ectosomes is a well-orchestrated cellular event wherein phospholipid redistribution coincides with the contractile machinery, which is primarily governed by cytoskeletal proteins. The cytoskeleton contractile machinery relies on a set of enzymes such as ADP-ribosylation factor 6 (ARF6) and myosin light chain kinase (MLCK). ADP-ribosylation factor 6 (ARF6) is a small GTPase protein that activates Phospholipase D and activated phospholipase D recruits extracellular signal-regulated kinase (ERK). ERK recruited at the plasma membrane activates MLCK via phosphorylation [70]. The biogenesis of ectosomes is completed through cytoskeletal contractions regulated by enzymes that govern the interaction between actin and myosin [60]. The phosphorylation of MLCK at Thr18/Ser19 induces the actin-myosin-based cytoskeletal contraction by generating the necessary force required for ectosome budding/shedding [70]. This enhances the activity of myosin II and the enhanced activity of Myosin II enables it to engage in highly efficient interactions with actin filaments increasing the cellular contraction [71]. A study on the regulation of the Rho/MLC pathway by ADP-ribosylation factor 1 (ARF1) for controlling breast cancer cell invasion demonstrated that ARF1 also functions like ARF6 and plays a crucial role in the contractile machinery of the cytoskeleton [72].

##### Ectosome biogenesis in disease and altered physiological conditions

Ectosomes biogenesis can be abruptly altered under pathological and altered physiological conditions. Under altered physiological conditions, biogenesis can be affected by several factors. Some of the factors affecting the biogenesis of ectosomes and the biogenetic mechanism involved are discussed below.

##### ARRDC1-mediated ectosome biogenesis

Ectosome biogenesis invariably exploits the tumor-suppressing gene 101 (TSG101) protein, and the endosomal sorting complex required for transport (ESCRT) machinery to produce ectosomes. A study on arrestin domain-containing protein 1-mediated ectosomes (ARMMs) demonstrated that Arrestin Domain Containing 1 (ARRDC1) recruits TSG101 to the surface of cells to produce ectosomes. The ectosomes produced are distinct from exosomes, as they are devoid of late endosomal markers such as CD63 and lysosomal associated membrane protein 1 (LAMP1), indicating that these vesicles are released by direct plasma membrane budding [73].

##### Hypoxic ectosome biogenesis

Investigation of the role of hypoxia-inducible factors (HIFs) in breast cancer invasion and metastasis revealed that hypoxia in breast cancer cells induces an increase in the expression of the Ras-related protein Rab-22A (RAB22A) which colocalizes with increased expression of ectosomes formation. Moreover, RAB22A had a limited influence on ectosomes formation under nonhypoxic conditions. The study suggested selective recruitment of RAB proteins under hypoxia conditions for the shedding of ectosomes [74]. Hypoxia can exacerbate liver inflammation and fibrosis through the activation of hypoxia-inducible factors in MASLD.

##### Hyaluronan production and ectosome biogenesis

Hyaluronan synthesis coincides with various physiological events involving rapid tissue remodeling phases, such as embryonic development, inflammation, wound healing, and malignant tumor formation. Rilla et al*.* (2013) revealed that hyaluronan synthesis enhances the secretion of ectosomes. It is hypothesized that the ectosomes are shed either from tips of hyaluronan synthase (HAS)-induced microvilli or through budding of the plasma membrane. It is believed that cells that synthesize high quantities of hyaluronan generally harbor microvilli, which can serve as platforms for the formation of ectosomes. HAS activity is also influenced by cholesterol, and cellular cholesterol influences the secretion of microvesicles; thus, this study hypothesizes that microvesicle secretion occurs at the plasma membrane because of confirmational changes caused in lipid rafts due to HAS-induced hyaluronan synthesis [75].

##### RhoA-mediated ectosome formation

Ras-related C3 botulinum toxin substrate 1** (**RAC1) and the Ras homolog gene family, member A (RhoA) signaling are important for promoting invadopodia or ectosomes in tumor cells. The Rho family proteins Rac 1 and Rho A act against each other, and the action of these proteins determines the switching of the tumor cell phenotype between ameboid and mesenchymal phenotypes, which are distinct from each other; the former is involved in the shedding of ectosomes and later in the utilization of invadopodia. Tumor cell-derived ectosome formation is driven primarily by the Rho-ROCK pathway, which involves ARF6 activation downstream [76]. RhoA-mediated ectosome formation may be actively involved in the production of cancer ectosomes in HCC.

##### Biogenesis of exosomes

Exosomes originate from the endosomes on exocytosis of multivesicular bodies. The biogenesis of exosomes is the most complex and well-coordinated cellular event. The complex cellular events of exosome biogenesis include several key events, such as endocytosis, early endosome formation, formation of multivesicular bodies (MVB), intraluminal vesicle (ILV) formation with molecular cargo sorting, multivesicular body maturation, and exosome release. Early endosome formation is the first step of exosome biogenesis and begins with the endocytosis, which can be clathrin-mediated, caveolin-mediated or clathrin-or-caveolin independent endocytosis [59].

#### Endocytosis and early endosome formation

##### Clathrin-mediated endocytosis

Cellular uptake was first visualized via glutaraldehyde fixation via electron microscopy in 1960, which led to the discovery of vesicles coated with proteinaceous substances. Clathrin was then identified as a major protein of proteinaceous coating around the vesicles being taken up. Clathrin-mediated endocytosis has been explained in detail in previous studies. It involves a clathrin-coated vesicle cycle with five stages: nucleation, cargo selection, clathrin coat assembly, vesicle scission, vesicle formation, and budding. Briefly, nucleation begins with membrane invagination driven by F-BAR (Fes/CIP4 Homology-Bin/Amphiphysin/Rvs) domain-containing proteins (FCHO proteins), epidermal growth factor receptor pathway substrate 15 (EPS15) and intersectins. The nucleation model then recruits clathrin for budding and adaptor protein complex 2 (AP2) for cargo selection. Clathrin then stabilizes the vesicle, while Dynamin enables scission. Heat shock cognate 70 (HSC70) disassembles the coat, allowing clathrin recycling [77].

##### Clathrin-independent endocytosis

Clathrin-independent endocytosis poses challenges due to membrane flexibility and restrictions in capturing molecular cargo in small areas. Caveolae-mediated endocytosis is one of the major types of clathrin-independent endocytosis machinery. Small pits on the plasma membrane characterized by proteins such as caveolin and cavins called caveolae can dynamically detach from the membrane to form endocytic carriers [78].

In addition to caveolae-mediated endocytosis, several clathrin-independent endocytosis pathways, including the clathrin-independent carrier/GPI-AP-enriched early endosomal compartment (CLIC/GEEC) pathway and the ARF6-associated pathway, are involved in endocytosis. The detailed mechanisms of these pathways are not well understood; however, reorganization of the actin cytoskeleton is a common key factor in all of these pathways [78, 79].

##### Early endosome formation

Endocytosis results in the formation of pleomorphic structures known as early endosomes. They play a central role in regulating the recycling and breakdown of membrane elements. Few components of early endosomes are recycled, whereas others are transported into trans-Golgi networks. The molecular cargo predetermined for late endosomes or EVs is sorted into intraluminal vesicles (ILVs), which results in the formation of multi vesicular endosomes (MVEs) [80]. Multivesicular bodies were initially considered important components of the endosomal lysosomal degradation pathway [81]. These multivesicular bodies have multiple fates; they can be sorted toward late endosomes, followed by delivery to lysosomes or the plasma membrane. The molecular cargo destined for degradation follows the former path, whereas the cargo involved in cellular communication through exosomes follows the latter path.

##### Cargo sorting, multivesicular body formation, and maturation

Although exosomes are tiny, they carry a wide variety of molecular cargo, including proteins, lipids, metabolites, and various forms of RNA, such as messenger RNA (mRNA), microRNA (miRNA), long noncoding RNA (lncRNA), circular RNA (circRNA), and PIWI-interacting RNA (piRNA) [59, 79, 82, 83]. The physiological state of parent cells from which exosomes are produced greatly influences the molecular profile of their cargo. Interestingly, exosomes attain definitive cellular functions on the basis of the molecular cargo they carry [84]. Hence, precise sorting of these cargoes is crucial for exosome biogenesis and function.

##### Protein cargo sorting

Ubiquitylation and farnesylation are two important posttranslational protein modifications that play prominent roles in the segregation of certain proteins into ILVs [85]. The sorting of molecular cargo occurs through ESCRT-dependent and ESCRT-independent pathways. ESCRT plays a key role in the formation of ILVs by incorporating specific protein cargo. The key components of the pathway include Hepatocyte Growth Factor-Regulated Tyrosine Kinase Substrate (HRS/ESCRT0), ESCRT (I, II, III), ALG-2-Interacting Protein X (ALIX), and Syntennin-1 [86]. They play critical roles in membrane scission during ILV formation, cargo selection, incorporate syndecans and other cargos into ILVs, and help in the formation or secretion of exosomes.

ESCRT associated pathways can also be involved in the formation of ILVs. The syndecan-synthenin and Alix pathways and His-domain protein tyrosine phosphatase pathways also allocate ESCRT III to form ILVs. Syndecan-synthenin and Alix can sort proteins such as CD63, CD81, CD82, CD9, and fibroblast growth factor receptor (FGFR) [87, 88]. The ESCRT- independent pathway for the formation of ILVs involves components of lipid rafts such as ceramides. Ceramides actively participate in ILV formation by playing an important role in membrane budding and curvature. Tetraspanins such as CD63 and TSPN6 can also contribute to ILV formation independent of ESCRT. Chaperones such as heat shock protein 70 (HSP70), HSC70, and GPI-anchored proteins can co-sort cytosolic proteins into ILVs and facilitate the incorporation of lipid domains into ILVs.

### Nucleic acid cargo sorting

RNA cargo sorting: RNA cargo can be sorted through multiple pathways, wherein RNAs can be directly incorporated into exosomes due to the presence of particular sequence motifs [89] or can be incorporated with the assistance of RNA-binding proteins such as RNA-induced silencing complex (RISC) and Argonaute 2 (AGO2) [90], ESCRT-assisted RNA sorting, or with the help of RNA binding proteins sequestered within tetraspanins enriched microdomains, or with the help of other proteins such as major vault protein and Y-box-binding protein 1 (YBX1) [91].

DNA cargo sorting: Although protein cargo sorting and RNA cargo sorting have been extensively studied, knowledge of sorting of DNA cargo into EVs is limited. Few recent studies have provided some insights into the potential mechanisms that might be involved in DNA cargo sorting. Yokoi et al. (2019) reported that in ovarian cancer cells, genomic DNA is sorted into exosomes through tetraspanins into multivesicular bodies where micronuclei formed during cancer collapse releasing genomic DNA, which is then shuttled to Multivesicular bodies. Similarly, mitochondria also serve as a precursor for DNA cargo for exosomes. The PTEN-induced putative kinase 1 (PINK1) protein released during mitochondrial damage facilitates the interaction of mitochondria and multivesicular bodies leading to the sorting of the mitochondrial cargo into MVBs [92]. Knowledge about the involvement of the ESCRT mechanism in DNA cargo sorting is lacking. However, some contradictory findings suggest that the extracellular secretion of DNA is histone-mediated and is exosome-independent in nature [93]. Future studies in this domain are essential for enhancing the understanding of the mechanisms involved in the sorting of DNA cargo into EVs.

#### Exosome release

**Fig. 1:**
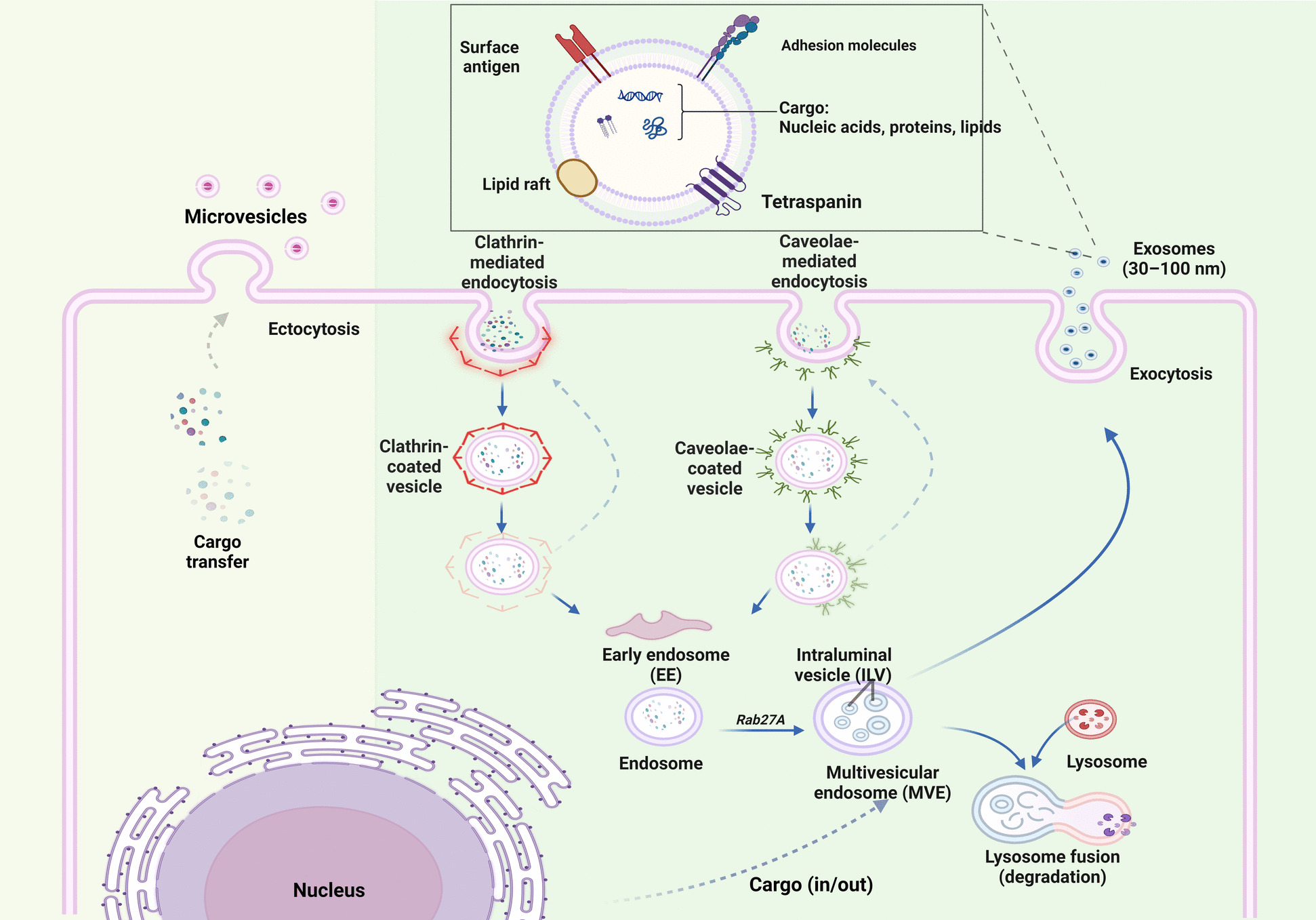
Extracellular vesicle biogenesis. **A** Ectosome Biogenesis: Ectosomes are released upon membrane blebbing from the cells, which typically involves specific changes in the lipid and protein components at certain plasma membrane sites. During ectosome formation, horizontal cargo sorting is followed by ectocytosis. **B** Exosome Biogenesis: Exosomes originate from endosomes via exocytosis of multivesicular bodies. Exosome biogenesis involves several key events, such as endocytosis, early endosome formation, MVB formation, ILV formation with molecular cargo sorting, multivesicular body maturation, and exosome release. Endocytosis can be clathrin-mediated or caveolin-mediated or clathrin or caveolin-independent endocytosis

Multivesicular bodies can attain a secretory or degradative fate, and MVBs destined to reach the secretory face translocate toward the plasma membrane and fuse with the plasma membrane marking the end of exosome biogenesis. Exosome release involves soluble N-ethylmaleimide-sensitive factor attachment protein receptors (SNARE) proteins which mediate membrane fusion events. The Fas/Fap-1/caveolin-1 cascade and long non-coding RNA HOX transcript antisense intergenic RNA (lncRNA HOTAIR) regulate SNARE formation in stem cells and hepatocellular carcinoma cells respectively [94, 95]. The fate of MVBs is also strongly influenced by cytoskeletal elements, such as actin and microtubules, which play crucial roles in transport, docking, and membrane fusion. Proteins such as Rab27a, Rab 7, and Rab 31 play essential roles in stabilizing docking sites, promoting exosome secretion, and interacting with motor cytoskeletal proteins. Additionally, divalent cations such as calcium ions play important roles in the regulation of Rab11-mediated exosome secretion pathways [59, 79, 84]. The biogenesis of ectosomes and exosomes is summarized in Fig. 1.

*Clathrin-mediated endocytosis* involves nucleation, cargo selection, clathrin coat assembly, vesicle scission, vesicle formation, and budding.

*Caveolae-mediated endocytosis* begins with invagination of the plasma membrane, which is rich in proteins such as caveolins and cavins. The caveolae then pinches off from the membrane to form vesicles that transport cargo into the cell.

*Cargo Sorting, MVB Formation, and Exosome release:* The molecular cargo within early endosomes is selectively sorted into intraluminal vesicles that form MVBs, which eventually fuse with the plasma membrane to release their contents as exosomes into the extracellular space.

*Exosomes:* Exosomes harbor diverse molecular cargo including nucleic acids, proteins and lipids.

### EV biogenetic pathways: bridging MASLD pathogenesis

Careful observation of EV biogenesis pathways indicates that various molecular signatures overlap MASLD. For example, CD53, a tetraspanin membrane protein involved in EV biogenesis and immune function, has been upregulated in hepatocytes following a high-fat diet and inflammatory triggers. The inhibition of CD53 was found to prevent diet-induced fat accumulation and liver inflammation, highlighting its role in integrating metabolic and inflammatory signals in hepatocytes and its potential as a therapeutic target for conditions such as MASLD and type 2 diabetes [96, 97]. Similarly, other components of EV biogenesis are implicated in disease pathogenesis and are summarized in Table 1.

**Table 1:** Role of EV biogenesis components in Liver diseases

Components of EV biogenesis	Role in disease	Disease and the model used	Remarks- Future prospective/ possible applications
CD53-Exosome cargo selection [96]	Mediates TNF-α and lipopolysaccharide proinflammatory signaling pathways associated with overnutrition	MASLD- dyslipidemia, MASH- *invitro* model derived from primary murine hepatocytes [97]	The inflammatory response in MASH possibly be inhibited by suppressing the expression of CD53
ESCRT- regulates secretion and composition of exosomes [98]	CHMP5 of the ESCRT-III family, is associated with the dysfunction of BSEP trafficking by apical targeting of the canalicular transporter BSEP in cholestatic liver diseases	Cholestatic Liver Diseases in vitro- hepatoma cell lines in vivo-human liver samples and hydrodynamically injected mouse model [99]	Targeting CHMP5 of ESCRT-III may provide a therapeutic strategy to encounter BSEP-associated cholestasis
Rab GTPase- plays a significant role in vesicle budding, trafficking, and fusion with the plasma membrane [100]	Upregulated GP73 activates Rab GTPase through its TBC domain, affecting ApoB activity thereby decreasing VLDL secretion and increasing lipid accumulation in the hepatocytes	MASLD in vivo* human* blood samples [101]	Targeting Rab GTPase especially to inhibit GP73's GAP activity through a common anti-diabetic drug may aid in improving the disease [101]
SNARE Protein Specially VAMP-7 mediates the fusion of (MVBs with the plasma membrane, facilitating exosome secretion in cancer cells [102]	SNARE protein complex, especially VAMP2 gets disrupted resulting in the accumulation of Ferroportin in activated Hepatic Stellate Cells leading to liver fibrosis	Liver fibrosis in vitro-HSC-LX-2 cells in vivo*-* mouse model of liver fibrosis induced by intraperitoneal injection of CCl_4_ [103]	-
TSG101-facilitates formation and cargo sorting of MVB [104]	TSG101 safeguards oncogenic protein PEG10, leading to overexpression of both TSG101 and PEG10 facilitating metastasis in HCC	Hepatocellular carcinoma in vitro models -HCC cell lines HepG2 and SMMC-7721 and in vivo models of tissue samples [105]	-
CHMP -formation of Sonic Hedgehog in EV subtype [106]	CHMP proteins are significantly elevated and are involved in elevated immune cell levels, resistance to drugs, and tumor progression in liver hepatocellular carcinoma (LIHC)	In-silico evaluation using differential expression analysis [107]	-
Alix- MVB formation and the inward budding of endosomal membranes [108]	Alix interacts with the HBV core protein and regulates the release of nonenveloped capsids from infected cells, independent of the ESCRT machinery in hepatitis B virus (HBV)-related liver disease [109]	HBV related liver disease in vitro*: HuH*-7 cells and MIHA immortalized hepatocyte cell line, Plasmid-based models, siRNA-mediated knockdown models [109]	-
Phospholipids- formation and stabilization of vesicles [110]	PLs, particularly elevated level of PEs and alteration in its composition results in MASLD-related complications by promoting steatosis, inflammation and mitochondrial dysfunction [111]	MASLD in vitro- HepG2 and LX2 cells [78]	-

*EV* Extracellular vesicle, *TNF-α* Tumor Necrosis Factor-alpha, *MASLD* Metabolic dysfunction Associated Steatotic Liver Disease, *MASH* Metabolic dysfunction-associated steatohepatitis, *ESCRT* Endosomal Sorting Complex Required for Transport, *CHMP5* Charged Multivesicular Body Protein 5, *Rab* Ras-associated binding proteins, *GTPases* Guanosine triphosphatases, *GP73* Golgi protein 73, *SNARE* Soluble N-ethylmaleimide-sensitive factor attachment protein receptors, *BSEP* Bile salt export pump, *VAMP* Vesicle-Associated Membrane Protein, *HSC- LX-2* Human hepatic stellate cell line, *CCl*_*4*_ Carbon tetrachloride, *VLDL* Very Low-Density Lipoprotein, *TSG101* Tumor Suppressing Gene 101, *MVB* Multivesicular body, *PEG 10* Paternally expressed gene 10, *HepG2* Hepatoblastoma cell line, *SMMC-7721* Human hepatocarcinoma cell line, *HCC* Hepatocellular carcinoma, *LIHC* Liver hepatocellular carcinoma, *HBV* Hepatitis B virus, *Alix* ALG-2-interacting protein X, *HuH-7* (hereafter Huh7)

### MASLD—a historical preview:

#### NAFLD-MASLD

Obesity, a major physical and physiological change, has been recognized as an important physiological event since prehistoric times. The prehistoric recognition of obesity is evident from the “Venus figurines” from the upper Paleolithic era, such as the Venus of Willendorf. While ancient civilizations, including those in Egypt and China, viewed obesity as a symbol of prosperity and fertility, the medical recognition of obesity began with the Indian physician Sushruta in the sixth century BC, who linked it to overindulgence and inactivity. European physicians and philosophers Hippocrates and Galen’s views on obesity were highly influential in medieval and renaissance Europe. They emphasized diet and exercise as primary ways to manage obesity, which has been practiced in medicine for centuries. Fatty liver disease was not identified as a distinct condition until the early nineteenth century [112]. From 1975–2018, global obesity rates tripled, coinciding with the introduction of food rich in high-fructose corn syrup [113]. Initially, the effects of these diet forms were directly linked with obesity; however, it took a long time to confirm the role of such dietary regimens in metabolic syndrome-related diseases such as MASLD. Although there is historical evidence for fatty liver disease, recent findings define the impact of obesity on fatty liver disease, providing a crucial connection. MASLD is a recently identified condition that is significantly driven by obesity. While the ancient people recognized and recorded obesity, related conditions such as MASLD reveal the long-standing consequences of obesity, which can be traced back to historical observations and practices concerning weight and health. Obesity has been prevalent since prehistoric ages; however, MASLD as a disease has been overlooked by the medical community.

Historical records suggest that the autopsy studies carried out during the nineteenth century revealed that hepatic steatosis was a common ailment affecting one-third of French and German populations, predominantly women and tuberculosis patients [112]. The earliest use of the term “fatty liver” dates to 1825, in Louis’s textbook of anatomy and pathology. It was then Thomas Addison in 1836 who introduced the term “fatty liver,” relating it to the presence of tuberculosis and alcohol consumption through histological differences [114]. Much more emphasis has been placed on understanding the mechanism of cirrhosis, as the initial liver manifestations leading to cirrhosis were not known at the time. Most diagnoses occurred at this advanced stage by 19th-century researchers, which ultimately led to the discovery that fatty infiltration in the liver due to metabolic disorders or alcoholism causes cirrhosis.

In the 1960s, "fatty liver hepatitis" emerged in the German literature, where the histopathological description of the liver with necroinflammation in obese individuals distinguished it from alcoholic steatohepatitis. In 1980, Ludwig et al. used the term nonalcoholic steatohepatitis (NASH) for the first time after inspecting liver biopsies of 20 patients who presented similar traits such as alcoholic steatohepatitis, including significant fat accumulation in the liver with signs of lobular hepatitis, focal necrosis, mixed inflammation, and often Mallory bodies, mostly in obese women with mild liver functional abnormalities and common fibrosis [115]. The term NAFLD was introduced to hepatology by Fenton Schaffner in 1986, and NASH progression to fibrosis and cirrhosis was reported by Randall Lee (American pathologist) in 1989 [116, 117]. Recently, the term NAFLD was changed to MASLD and NASH, now replaced with the term MASH) in early 2020s to better reflect the root cause for the disease, including cardiometabolic risk factors, and to reduce stigmatizing language associated with the words “nonalcoholic" and "fatty.". This change from NAFLD to MASLD was driven by global collaborative efforts led by the American Association for the Study of Liver Diseases (AASLD), the European Association for the Study of the Liver (EASL), and *Asociación Latinoamericana para el Estudio del Hígado* (Latin American Association for the Study of the Liver) (ALEH) with the Delphi proceedings to achieve consensus among experts from various fields [118, 119].

#### MASLD diagnosis

The understanding and diagnosis of MASLD have significantly evolved over the past 5 decades. The earliest milestone in diagnosing this condition dates to the post-World War II era when it was observed that nonalcoholic individuals exhibit symptoms similar to those caused by alcohol. The drastic shift in research focus toward understanding MASLD progression and establishing diagnostic criteria occurred in the 1990s leading to the development of histological grading and staging systems for MASH and the assessment of steatosis, ballooning, inflammation, and fibrosis [120]. MASLD scoring systems were introduced in 1999 by the NASH Clinical Research Network with standardized methods and protocols to quantify disease activity, disease stage, and fibrosis which essentially guided clinical trials and research [121].

**Fig. 2:**
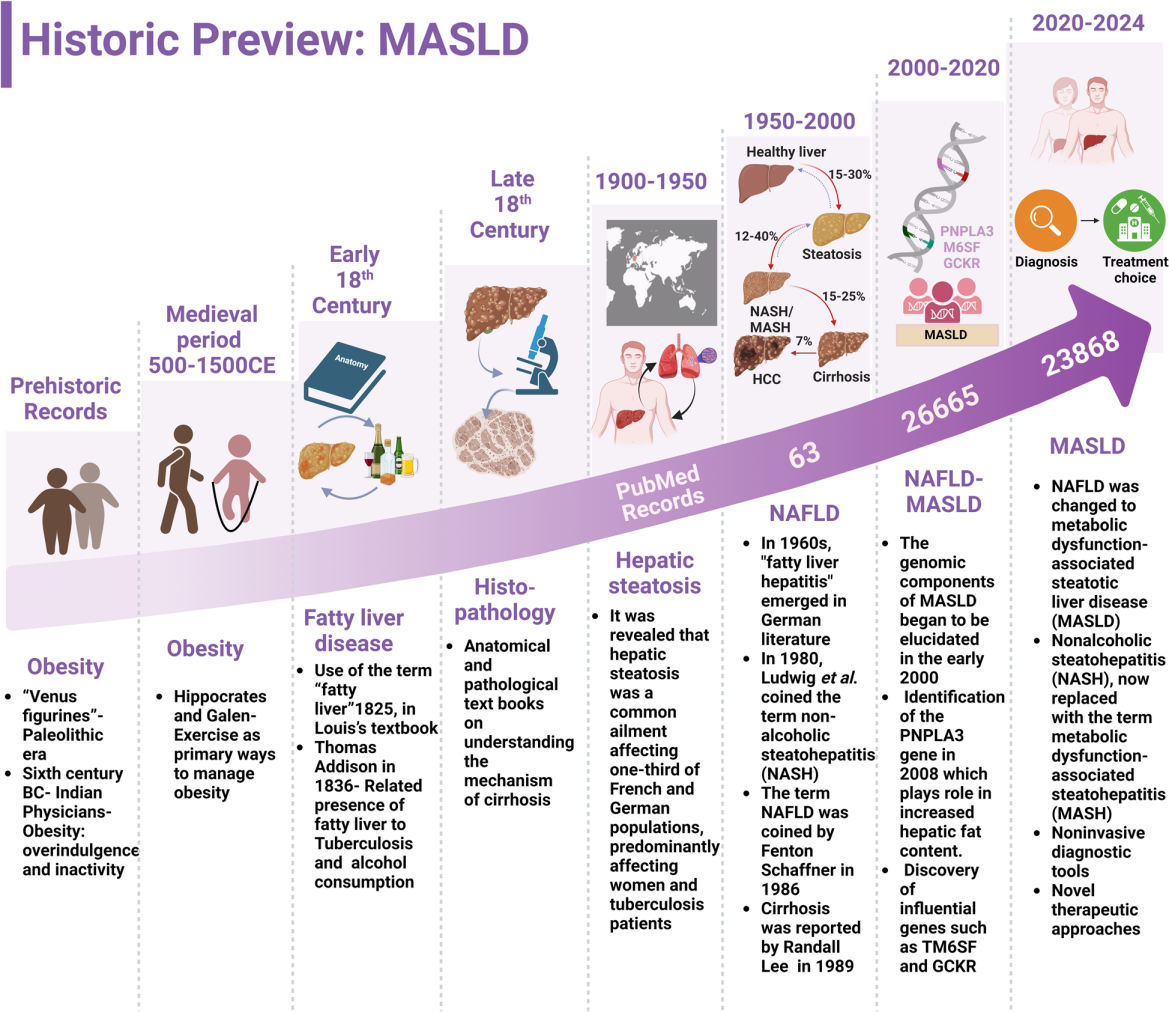
Historic preview of MASLD- The understanding of MASLD pathology and the development of therapeutic modalities for MASLD have evolved from prehistoric times to the present day. The historical overview of MASLD highlights the key milestones in understanding of the disease

The genomic components of MASLD began to be elucidated in the early 2000s, with significant advancements in 2008, with the identification of the PNPLA3 gene as a key factor in increased hepatic fat content [122, 123]. This discovery highlighted genetic predisposition to NAFLD along with the subsequent discovery of influential genes such as TM6SF and GCKR [124–126]. The increasing prevalence of MASLD has prompted the scientific community to focus on its diagnosis, highlighting several noninvasive diagnostic methods between 2007 and 2015. Noninvasive diagnostic methods such as the NAFLD Fibrosis Score (NFS), Fibrosis-4 (FIB-4) index, and vibration-controlled transient elastography were developed and became popular during this period [24, 127, 128]. These noninvasive tools have increased the ability to accurately diagnose advanced fibrosis without the need for liver biopsy. By 2015, fibrosis was identified as a crucial prognostic and diagnostic indicator in MASLD and was utilized for predicting overall and liver-specific mortality. Following 2020, the focus remained on refining and sensitizing the noninvasive diagnostic tools and developing advanced technologies aided with machine learning algorithms and accurate histological assessments. Several research consortia such as Liver Investigation Testing Marker Utility in Steatohepatitis (LITMUS) and Non-Invasive Biomarkers of Metabolic Liver Disease (NIMBLE) are aimed at identifying new biomarkers and validating these biomarkers for the diagnosis of early MASLD stages such as MASH, to reduce the reliance of the whole diagnostic sector for MASLD on invasive liver biopsy procedures. However, despite these recent developments in MASLD diagnostics research, liver biopsy continues to be the gold standard for diagnosing MASH and early-stage fibrosis which emphasizes the need for further advancements in diagnostic methodologies. A historic preview of MASLD from prehistoric times to the present day is presented in Fig. 2.

### Guidelines for the diagnosis of MASLD

**Fig. 3:**
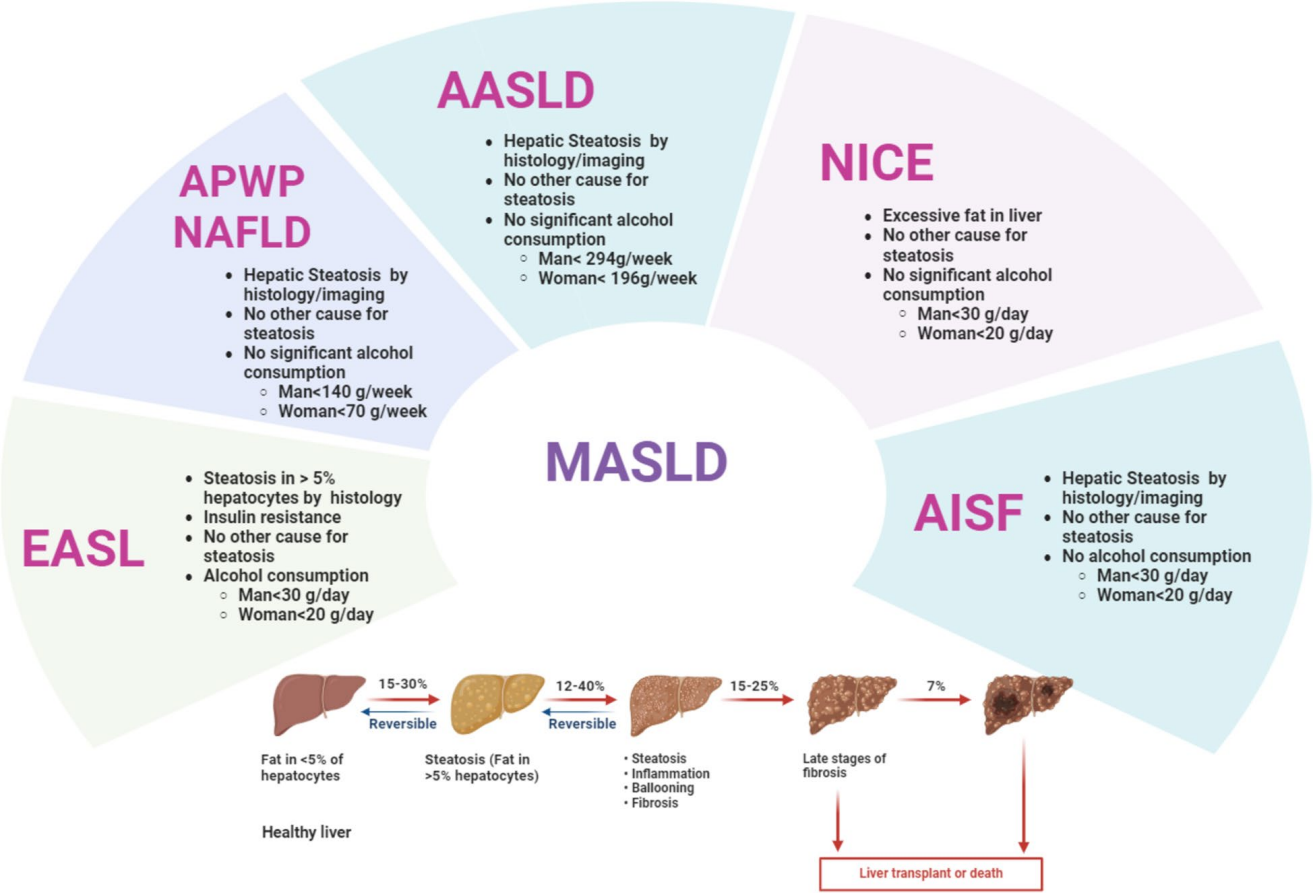
**a** Guidelines for MASLD diagnosis produced by the European Association for the Study of the Liver (EASL), **b** Asia–Pacific Working Party on NAFLD (APWP-NAFLD), **c** American Association for the Study of Liver Diseases (AASLD), **d** National Institute for Health and Care Excellence (NICE), and e.) Italian Association for the Study of the Liver (AISF)

The diagnosis of MASLD is guided by protocols established by several prominent liver disease associations. These guidelines are produced by the EASL, the Asia–Pacific Working Party on NAFLD (APWP-NAFLD), the American Association for the Study of Liver Diseases (AASLD), the National Institute for Health and Care Excellence (NICE), and the Italian Association for the Study of the Liver (AISF). Each of these organizations provides comprehensive criteria and diagnostic tools, which are summarized in Fig. 3, offering a consolidated reference for clinicians to accurately diagnose MASLD.

### MASLD diagnosis and challenges

MASLD is a silent nonsymptomatic disease generally diagnosed through unintentional clinical or imaging tests. Although it is a slowly progressive disease with no symptoms during the early stage, fatigue, abdominal discomfort, and jaundice are the initial common indicators for suspecting the presence of disease [129]. In addition, some risk factors such as dyslipidemia, obesity, insulin resistance, type 2 diabetes, metabolic syndrome, improper diet, physical inactivity, and sleep apnea make individuals more prone to the disease than the general healthy population [130].

#### Existing diagnostic tools

Current diagnostic approaches for MASLD include various invasive and noninvasive techniques.

##### Noninvasive diagnostic approach-MASLD scoring systems using blood-based biomarkers

**Fig. 4:**
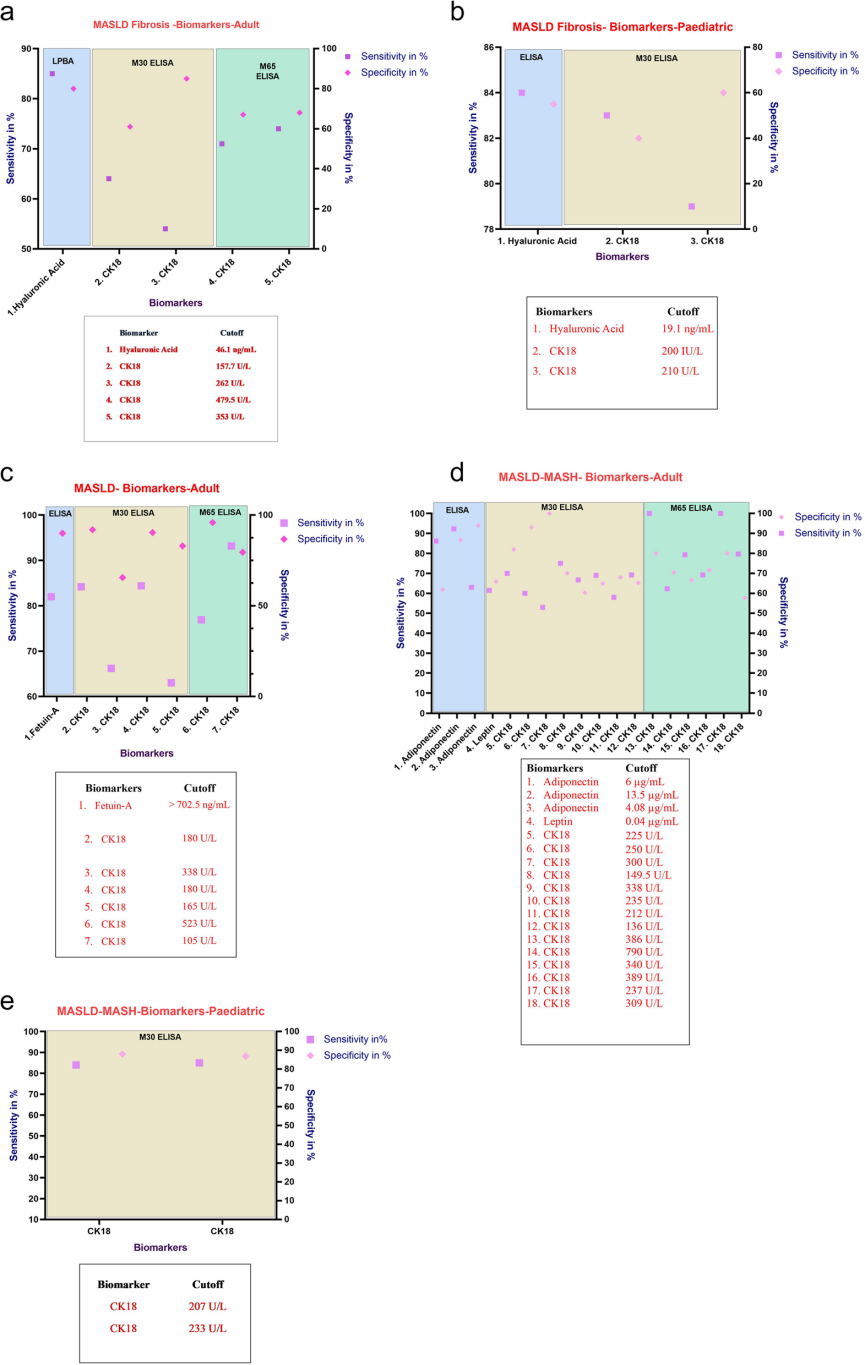
**a** Biomarkers for MASLD: Sensitivity and specificity with the cutoff value for noninvasive blood-based biomarkers of MASLD-Fibrosis Adult population. **b** Biomarkers for MASLD: Sensitivity and specificity with the cutoff value for noninvasive blood-based biomarkers of the MASLD—Fibrosis Pediatric Population. **c** Biomarkers for MASLD: Sensitivity and specificity with the cutoff value for noninvasive blood-based biomarkers of the MASLD spectrum specific adult population. **d** Biomarkers for MASLD: Sensitivity and specificity with the cutoff value for noninvasive blood-based biomarkers of the MASLD- MASH- Adult population. **e** Biomarkers for MASLD: Sensitivity and specificity with the cutoff value for noninvasive blood-based biomarkers of the MASLD MASH- Pediatric population

The diagnosis of MASLD usually starts with elevated liver enzyme levels, typically elevated alanine aminotransferase (ALT) levels compared with aspartate aminotransferase (AST) levels. The use of these enzyme levels as markers, along with other markers, such as gamma-glutamyl transferase (GGT) and alkaline phosphatase (ALP), provides a comprehensive understanding of the liver condition. Although ALT is liver specific, it is also altered in non-MASLD conditions. Furthermore, owing to its low specificity, MASLD cannot be solely dependent on liver enzymes [131, 132]. Even though ALT is frequently used as a biomarker due to its affordability and availability, its results depend upon overall liver function, and it may not exclusively indicate MASLD. For example, ALT has also been utilized in determination of metabolic syndrome [133]. Similarly, several stage-specific and disease-specific molecular signatures for MASLD have been identified in the past decade. The noninvasive blood-based molecular signatures for MASLD with sensitivity and specificity and their expression patterns in disease patients and relevant studies are summarized in Fig. 4 and Supplementary Table S1 [134–147].

However, the limitations associated with these biomarkers diminish their reliability when used alone for disease identification. Therefore, diagnostic scores or indices are usually used to diagnose the risk and degree of the disease, which are calculated by estimating the synergistic outcomes of clinical parameters and hematological parameters [148]. The diagnostic indices are generally calculated on the basis of the combination of specific panels pertaining to the organ of interest. A panel can be defined as a group of medical diagnostic tests generally recommended by physicians that can provide comprehensive information about a particular organ system, disease state, or function. The appropriate index for a disease state or condition is the one that considers a comprehensive range of diagnostic factors along with clinical parameters to assign a score. This approach ensures a more accurate and holistic assessment of the disease, considering various aspects of the patient's health and specific characteristics of the condition being evaluated. The indices and panels used to evaluate the MASLD risk are listed in Table 2.

**Table 2:** Noninvasive scores/indices and panels used to evaluate the risk of MASLD

Index/Pannel	Description	Utility and Limitations
**Fatty Liver index (FLI) = **(e ^0.953×loge (Triglycerides) +0.139×BMI+0.718×loge (GGT) +0.053×Waist circumference−15.745^) / (1 + e ^0.953×loge (Triglycerides) +0.139×BMI+0.718×loge (GGT) +0.053×waist circumference−15.745^) × 100	• Algorithm basis: BMI, Waist circumference, Serum TG, GGT • Accuracy: 0.84 (95%CI 0.81–0.87) in detecting Fatty Liver [149] • FLI Range: 0 and 100 • FLI Interpretation: FLI ≤ 30 (negative likelihood ratio = 0.2) rules out fatty liver disease and FLI ≥ 60 (positive likelihood ratio = 4.3) rules in fatty liver disease	**Utility**: It is simple to calculate which can assist physicians in selecting patients for ultrasonography and recommending lifestyle modification. It is also greatly utilized by researchers to select patients for epidemiological studies **Limitations:** It has low accuracy in identifying several grades of steatosis [150] and hence validation in the external population is necessary before adopting it for clinical purposes
**Triglyceride and Glucose Index (TyG) = **ln [Triglycerides (mg/dl) Fasting Blood Glucose (mg/dl)/2] [151]	• Index is influenced by Triglyceride level and Fasting Blood Glucose • TyG Index is utilized in determining the risk of metabolic syndrome, T2D and cardiovascular diseases • Accuracy: TyG has Greater predictive performance for MASLD with the AUROC of 0.782 compared to ALT Test alone. Additionally, at the cut off 8.5 Tyg has the sensitivity and specificity of 72.2% and 70.5%, respectively demonstrating TyG has better predictive capability for diagnosing MASLD [152]	**Utility:** TyG Index is a mathematical model developed based on Mexican population, it was previously used for analyzing insulin resistance in Chinese populations [153]. Later, on, the relationship between the insulin resistance and hepatic fat content was established in the literature [153]. It can be utilized for determination of MASLD risk **Limitations**: TyG Index determines the Insulin resistance however it cannot differentiate the degree and cause of insulin resistance It also does not direct information about insulin level as it takes account of fasting sugar level Validation of this index in different population and ethnic group is required to clinically implicate this particular parameter for MASLD diagnosis
**Hepatic steatosis index (HIS) = **8 × ALT/AST + BMI + 2 (if diabetic) + 2 (if female)	• The index is derived from the high-risk factors for MASLD obtained from multivariate analysis • Algorithm basis: ALT/AST, BMI, Sex, and Presence of Type 2 diabetes • Accuracy: HSI value of > 36.0 could detect MASLD with a specificity of 93.1% (95% CI: 92.0–94.0) and a positive likelihood ratio of 6.505 (95% CI: 5.628–7.519) [154]	**Utility:** Prescreening tool, Selection of populations for Epidemiological studies **Limitations:** The study by which the HSI was derived showed an AUROC value of 0.812. Although acceptable, it indicates that HSI is not perfect and there is room for improvement Population Specificity: The findings are specific to Korean populations; hence a pilot study is required in the population of interest before adopting this scoring system Limited Scope: HSI is not dependent on all the factors contributing to MASLD and additional clinical parameters may be needed for comprehensive screening ALT/AST Ratio dependent: The reliability of HSI is dependent on accurate measurements of ALT and AST, which can vary between laboratories [150, 154]
**NAFLD Liver Fat Score** (NLFS) = { -2.89 + 1.18 × MetS (yes = 2, no = 0) + 0.45 × type 2 diabetes (yes = 2, no = 0) + 0.15 × fasting serum insulin (mU/L) + 0.04 × AST (U/L)—0.94 × AST/ALT ratio}	• The index considers the key components such as Metabolic syndrome, Serum insulin, ALT, and AST levels. The scores above-0.640 show a high likelihood of MASLD and a score below -0.640 rules out MASLD • NLFS values greater than -0.64 have shown a sensitivity of 86% and a specificity of 71% for identifying hepatic steatosis greater than 5.56% • NLFS values are specifically associated with increased liver mortality [155]	**Utility:** NLFS can be used as a noninvasive prescreening tool, for Clinical Decision Making, Epidemiological Studies, and Risk Stratification **Limitations:** The determinant depends on insulin level, which is not a routinely used test, limits its use in clinical practice [150]. It has moderate specificity and accuracy is dependent upon metabolic syndrome and diabetes
**SteatoTest (Panel)**	• Six Fibro Test elements (α2-macroglobulin, haptoglobin, apolipoprotein A1, GGT, total bilirubin and ALT) in addition to BMI, cholesterol, triglycerides, and glycemia • For the diagnosis of grade 2–4 steatosis, the sensitivity of SteatoTest at the 0.30 cutoff was 0.91, 0.98, 1.00, and 0.85 and the specificity at the 0.70 cutoffs was 0.89, 0.83, 0.92, 1.00, for the training and three validation groups, respectively [156]	**Utility:** A simple and noninvasive quantitative estimate of liver steatosis may reduce the need for liver biopsy, particularly in patients with metabolic risk factors **Limitations:** The test is adjusted for sex and age and demonstrates moderate accuracy in predicting hepatic steatosis. The test is widely not employed as it is not accurate in differentiating the steatosis stages and is expensive [150]
**Enhanced liver fibrosis (ELF) test** Discriminant score = -7.412 + [ (ln (HA) × 0.681) + (ln (P3NP) × 0.775) + (ln (TIMP1) × 0.494)] + 10	• It is derived from a simple algorithm which considers biomarkers such as HA, PIIINP, and TIMP-1 • Cutoff values for the prediction of “any” (> stage 1), moderate perisinusoidal (> stage 1b), moderate portal/periportal (> stage 1c), significant (> stage 2), or advanced (> stage 3) fibrosis were 0.92/9.28, 0.92/9.33, 0.90/9.54, 0.98/ 10.18 and 0.99/10.51, respectively [157]	**Utility:** A simple and noninvasive quantitative estimate of liver fibrosis can reduce the need for liver biopsy, particularly in patients with metabolic risk factors has been validated in 7 independent patient populations which ensures robustness across the groups **Limitations:** Biomarker variability, moderate precision, and specificity
**NAFLD Fibro-Meter (Panel)**	• It is a non-invasive hematological test used to evaluate fibrosis. It combines weight, the patient’s age, and six blood biomarkers to calculate a fibrosis score • The six blood-based biomarkers include Platelet count, AST, ALT, Ferritin, and Fasting glucose • The test scores range from 0–1 which are then correlated with METAVIR fibrosis scores F0-F4 [158–160] • The NAFLD Fibro Meter has shown good accuracy in predicting significant fibrosis, with AUROC values typically ranging from 0.80 to 0.85 in different studies	**Utility:** The fibro test can often be combined with transient electrography to increase its diagnostic accuracy. The study where transient electrography was combined with a fibro meter and incorporated additional parameter liver stiffness measurement has shown enhanced liver fibrosis prediction in the Chinese population [159]. Similarly, a comparative review of fibro test has revealed that the parameters utilized are already being tested in several countries and hence it states that it could be used as the first line of diagnosis instead of an invasive riskier biopsy [160] **Limitations:** Biomarker variability, population, specificity, and dependence on clinical variables
**Hepascore**	• It is a noninvasive hematological test used to evaluate fibrosis. It combines patient age, gender, and blood biomarkers to calculate a fibrosis score. Hepascore is calculated on the basis biomarkers including Bilirubin, GGT, Hyaluronic acid, and Alpha2-macroglobulin • Hepascore ranges from 0 to 1 with higher scores indicating the probability of advanced liver diseases • A study on comparative accuracy of Hepascore and Fibroscan in NAFLD patients has reported that Hepascore has greater accuracy than simple fibrosis tests for advanced fibrosis and greater accuracy than fibroscan in obese individuals [161]	**Utility:** A simple and noninvasive quantitative tool used to estimate liver fibrosis. It may reduce the need for liver biopsy, particularly in patients with metabolic risk factors. It helps in clinical decision making, patient monitoring, risk stratification, and patient monitoring cost effectively **Limitations:** Biomarker variability, population specificity, and moderate specificity which is not sufficient for clinical scenarios which require detailed fibrosis staging
**BARD Score**	• Noninvasive tool used to evaluate and predict advanced liver fibrosis in patients with MASLD disease. It basically includes 3 variables such as BMI, AST/ALT ratio, and presence of type 2 diabetes mellitus • Individuals with a BMI greater than 28 and an AST/ALT ratio above 0.8 are each assigned 1 point. Similarly, the presence of type 2 diabetes mellitus adds 2 points to the score • The BARD score ranges from 0 to 4 with higher scores indicating advanced fibrosis • Original study by Harrison et al. which described the BRAD score showed that a BRAD score of 2 or more had a higher negative predictive value of 96% in determining advanced fibrosis (F3-F4) [155] • The BARD score is also has been validated in Polish populations by subsequent study has shown the moderate accuracy of BARD in detecting advanced fibrosis. In Polish populations, it had a sensitivity of 86.7% and specificity of 72.7% using a cutoff of ≥ 2 points [162]	**Utility:** A simple and noninvasive, inexpensive tool used to evaluate liver fibrosis. It reduces the need for liver biopsy, particularly in patients with metabolic risk factors. It helps in clinical decision making, patient monitoring, and risk stratification particularly in ruling out fibrosis and patient monitoring cost effectively The enhanced version of BARD score called BARDI has included international normalized ratios have significantly improved the positive predictive value over BARD and have maintained a good negative predictive value [163] **Limitations:** Biomarker variability, population specificity, and moderate specificity which is not sufficient for clinical scenarios which require detailed fibrosis staging
**AST to platelet ratio** APRI = (Platelet count/AST​) × 100	• AST to platelet ratio is used to assess the fibrosis in MASLD patients. It is reported as the preferred index over AST to ALT ratio in determining hepatic fibrosis in MASLD [164, 165]	**Utility:** Noninvasive assessment and ease of computation make it an initial screening tool **Limitations:** APRI has modest diagnostic performance for advanced fibrosis in NAFLD, with AUROCs ≤ 0.70
**Visceral Adiposity Index (VAI)** Males VAI = (WC/36.68 + (1.88 × BMI)) × (TG/1.03) × 1.31/HDL) Females VAI = (WC/36.58 + (1.89 × BMI)) × (TG/0.81) × 1.52/HDL)	• Obesity is one of the key factors for MASLD and visceral obesity is often associated with increased insulin sensitivity, proinflammatory activity, and adipocytokine production • Visceral Adiposity Index is a gender-specific index used to determine visceral adiposity functionality that could be further correlated with steatosis [166]. It is calculated based on WC, BMI, TG, and HDL-C. VAI = 1 in healthy individuals • It has moderate accuracy in evaluating cardiometabolic risk [167]	**Utility:** Noninvasive assessment of Cardiometabolic risk, and intervention monitoring **Limitations:** The VAI has limited accuracy in distinguishing moderate and severe hepatic steatosis. It is not specific to hepatic fat quantification
**NASH Test**	• The NASH test is used to diagnose non-alcoholic steatohepatitis which evaluates MASH risk based on factors including age, sex, height, weight, and serum levels of TGs, cholesterol, a-macroglobulin, apolipoprotein A1, haptoglobin, GGT, ALT, AST, and total bilirubin • A study on the diagnostic value of biochemical markers for the prediction of NASH has shown 94, 33, 66, and 81%, specificity, sensitivity, PPV, and NPV, respectively [168]	**Utility:** The test is designed to provide a comprehensive assessment of liver function and disease activity Noninvasive assessment of MASH, and intervention monitoring
**NASH Diagnostic panel**	• The diagnostic panel developed by Younossi et al*.* to predict the NASH in patients with NAFLD proven through biopsy, is estimated based on variables including diabetes, gender, BMI, TGs, and (not routinely employed) cytokeratin markers such as M30 (apoptosis) and M65–M30 (necrosis) [169, 170] • Data analysis revealed that the levels of M30 antigen (cleaved CK-18) predicted histological NASH with 70% sensitivity and 83.7% specificity and area under the curve (AUC) = 0.711, *p* < 10 (-4), whereas the predictive value of the levels of intact CK-18 (M65) was higher (63.6% sensitivity and 89.4% specificity and AUC = 0.814, *p* < 10(-4) [171]	**Utility:** Noninvasive tool to predict NASH in patients with NAFLD. It helps in monitoring the progression of the disease and the interventions **Limitations:** It included uncommon variables such as CK-18 markers which limits its universal application for the usage
**NASH NIS4**	• The NASH NIS4 blood-based diagnostic test quantitatively measures independent four biomarkers such as microRNA 34a-5p, A2M, HbA1c, and CHI3L1 to calculate the NIS4 scores that range from 0 to 1 • The NIS4 test is designed to identify patients at risk of NASH with NAFLD activity score of more than 4 and significant liver fibrosis score of less than 2 [172]	**Utility:** The scoring system is used to decide the therapeutic decisions and monitor the disease progression in patients with NASH. It is also employed to identify the patients at risk for a physician to suggest a need for lifestyle modifications and to differentiate them from those who need aggressive treatments **Limitations:** Involves uncommon biomarkers which are not routinely employed for diagnosis such as microRNA 34a-5p, and CHI3L1 also known as YKL40 making its limited applicability

*FLI* Fatty Liver Index, *TyG* Triglyceride-Glucose, *BMI* Body mass index, *TG* Triglycerides, *GGT* Gamma-glutamyl transferase, *HIS* Hepatic steatosis index, *ALT* Alanine aminotransferase, *AST* Aspartate aminotransferase, *MASLD* Metabolic dysfunction-associated steatotic liver disease, *CI* Confidence interval, *AUROC* Area Under the Receiver Operating Characteristic Curve, *NLFS* NAFLD Liver Fat Score, *ELF* Enhanced liver fibrosis test, *HA* Hyaluronic Acid, *PIIINP* Amino-terminal Propeptide of Type III Collagen, *TIMP-1* Tissue Inhibitor of Metalloproteinase 1, *METAVIR fibrosis scores* Meta-analysis of Histological Data in Viral Hepatitis Fibrosis Scales, *NAFLD* Nonalcoholic fatty liver disease, *APRI* AST-to-platelet ratio, *VAI* Visceral Adiposity Index, *WC* Waist circumference, *HDL-C* High-density lipoprotein cholesterol, *PPV* Positive predictive value, *NPV* Negative predictive value, *CK-18* Cytokeratin (CK)-18, *NASH* Nonalcoholic steatohepatitis, *A2M* Alpha-2 macroglobulin; HbA1c

Despite the availability of noninvasive scoring systems to evaluate the risk of MASLD, including the various common panels and indices listed in Table 2, many such statistically derived scoring systems have several limitations that prevent their reliance solely on them for the assessment of MASLD. A major limitation associated with the scoring system is population heterogeneity, as these indices are generally calculated on the basis of small, clustered populations in a hospital setting, and actual disease prediction and risk evaluation become challenging. The lack of validation studies in different populations and the ability to specifically differentiate the risk of MASLD from other liver diseases pose significant hurdles in this domain. Specificity could be achieved by the addition of MASLD-specific biomarkers to the scoring systems or panels, which again limits the widespread utility of such panels or scoring systems.

##### Noninvasive diagnostic approach—imaging techniques

The evaluation of liver health extends beyond the basic assessment of liver enzymes. In the absence of hepatitis B, C, or other causes of chronic liver disease, elevated liver enzymes drive the clinician’s attention toward identifying underlying conditions such as hepatic steatosis. However, diagnosis of such conditions requires more than just clinical suspicion, as it demands an accurate and efficient way of evaluating the disease. In such a scenario, the evaluation is carried out with the help of imaging techniques or histological techniques. The imaging techniques primarily employed include ultrasound, computed tomography (CT), magnetic resonance imaging (MRI), magnetic resonance elastography (MRE), and transient elastography (fibro scan). The commonly employed imaging techniques for MASLD diagnosis are summarized in Table 3. The widespread availability of these noninvasive tools makes them go-to choices for initial screening and diagnosis.

**Table 3:** Imaging biomarkers/devices/techniques used for MASLD diagnosis

Imaging Device/Test/Biomarker	Stage	Description
Elastography	MASH and MASLD associated Fibrosis	• Elastography has been used in the assessment of liver fibrosis and stiffness. The technique is invariably employed for the identification of moderate to severe liver diseases • It is widely accepted and recommended by the World Health Organization (WHO) for assessing liver fibrosis by measuring liver stiffness through shear wave speed which is inversely proportional to liver stiffness [173] • Studies have shown that elastography can be combined with several biomarkers to improve the accuracy of results [159] • There are several types of elastography commonly employed for diagnosis of liver ailments including Transient elastography (TE), Magnetic Resonance Elastography (MRE), Shear wave elastography (SWE), and Strain elastography • Even though it is noninvasive and accurate in the identification of the disease; limited penetration of ultrasound waves deep into tissues, shadows of rib and lung, respiratory and cardiac motion, and vessel pulsation distorting ultrasound signal variation can cause an impact on accurate and reliable measure of liver stiffness. Furthermore, operator dependency of strain elastography and selection of site for detection and positioning brings key variability in results [174, 175]
Fibroscan or VTCE	MASH, and MASLD associated Fibrosis	• Fibroscan is a noninvasive diagnostic device used to evaluate liver scarring, which relies on elastography to measure liver stiffness • A study on diagnostic performances of various noninvasive methods in diagnosing liver fibrosis revealed that the Fibroscan probes M and XL showed AUROC of 0.88 and 0.85, respectively for advanced fibrosis [176] • Fibrosis assessment is generally carried out using CAP score or fibrosis scores • Fibroscan is error-prone in individuals with ascites, obesity, and larger chest fat volume. The failure or noninterpretable rates in Fibroscan is somewhere between 5–15% and the interpretation of fibroscan should be done with the medical history of individuals to comprehensively analyze the liver health which further limits its applicability [177, 178]
CAP (controlled attenuation parameter)	MASLD associated Fibrosis	• CAP is a method for detecting and quantifying liver steatosis. It works on the properties of radiofrequency signals that are backpropagated which are acquired by an ultrasonic sound-based vibration transient elastography system • The Fibroscan® (Echosens, Paris, France) is a well-known device used to characterize liver elasticity associated with liver fibrosis. The attenuation of ultrasound waves by the fat is well known and CAP is devised to utilize the degree of attenuation of ultrasound to determine steatosis [179] • The accuracy of CAP has been validated using simulation studies, tissue mimetic studies, and in vivo studies suggesting promising results in the detection of hepatic steatosis [179] • Ease measurement of steatosis, operator independence, and measurement of liver stiffness simultaneously aids in accurate diagnosis [180] • The existing data on CAP validation is largely from adult chronic liver disease patients, which might pose a significant hurdle for the clinical implication of CAP. Low sensibility for the low grade of steatosis limits its utilization in clinical evaluation of MASH [181]
Elastography conducted through the Acoustic Radiation Force Impulse (ARFI) method	Fibrosis	• ARFI elastography is used to quantitatively evaluate liver fibrosis and liver stiffness • This elastography uses high-frequency short duration ultrasonic pulses. The generated ultrasonic pulses cause displacement in the tissue, which leads to the generation of a shear wave which propagates transversely to the impulse delivered. The shear wave generated can be monitored spatially and temporally using ultrasonic monitoring units. The hepatic elasticity can be calculated which will be further represented as meters per second [182] • Study on the diagnostic accuracy of elastography with the ARFI technique has shown that the ARFI has a higher capability of discriminating severe fibrosis and cirrhosis (AUROC: 0.74–0.97 for F3; AUC: 0.78–0.89 for F4) rather than less severe stages (AUC: 0.70–0.83 for F2) [183] • However, the technical operation requires high-quality ultrasound systems and specialized training for optimal performance
2D shear wave elastography (2D-SWE)	Fibrosis	• 2D-SWE is utilized to evaluate liver stiffness, an important parameter assessed to diagnose fibrosis. It generally uses acoustic radiation to generate shear waves in liver tissue which are then captured in real-time and displayed as a color-coded image allowing qualitative and quantitative assessment of the structural status of the liver [183] • A recent study suggests that the 2D-SWE can be used as an alternative to fibro scan as it has similar diagnostic performance (86.7% agreement to the fibroscan results) and it surpasses fibro scan in obtaining adequate interpretable results in obesity and ascites [184] • 2D SWE also poses challenges as it may tend overestimation of fibrosis (11.7%) results [184]
Magnetic resonance electrography (MRE)	Fibrosis	• MRE, a combination of MRI and elastography used to analyze the stiffness of the organ particularly in liver tissue. An acoustic driver generates low-frequency vibrations which generate shear waves, that on propagation through the liver are captured by MRI pulse generating the image. The images are usually then analyzed for the generation of quantitative output indicating liver stiffness • A systematic review on the diagnostic performance of the MRE in staging liver fibrosis concluded that the MRE has a high accuracy in the diagnosis of advanced or significant fibrosis with the AUROC of 0.86–0.91 for each stage of fibrosis [185] • Recently the three-dimensional magnetic resonance elastography combining proton fat fraction was used to identify fibrosis associated with the MASLD [186] • Availability, operational cost and safety concerns in certain individuals with implanted devices limit the use of MRE as a diagnostic modality
Magnetic resonance imaging- Proton density fat fraction (MRI-PDFF)	MASLD associated Fibrosis	• The noninvasive MRI-based imaging technique is used for assessing liver fat content. It has emerged as an accurate (MRI-PDFF diagnosed S1–3 steatosis, with a sensitivity of 0.95 (95% CI, 0.92–0.97), specificity of 0.92 (95% CI, 0.77–0.98)) reproducible biomarker for hepatic steatosis [187] • MRI-PDFF utilizes the echo sequence gradient. By minimizing T1 bias (T1 bias is a strong confounder for MR-based fat quantification) and by increasing echoes to correct T2 effects (length of time it takes for the MR signal to decay in the transverse plane) for calculating the fat accumulation. The obtained signals are then modeled to accurately determine the fat and water proton densities. This accurately infers the fat content of the liver [187, 188] • MRI-PDFF is more precise than CAP in detecting several steatosis grades and in determining liver fat accumulation in MASLD patients. However, time-consuming nature of the diagnosis, availability, need for qualified personnel, and economic burden associated with the diagnosis limit its utility worldwide. Furthermore, even though MRI-PDFF can accurately measure the fat accumulation in liver, the standard ranges to evaluate the disease stage are not well defined
Multiparametric magnetic resonance imaging (mp MRI)	MASH Fibrosis	• The mp-MRI measures various aspects of liver functions and structure by combining multiple imaging modalities. Liver fibrosis is assessed through transient elastography or shear wave elastography, liver fat content is evaluated using MRI PDFF, and liver function through liver perfusion, blood flow, and oxygenation • It has an accurate capability of early detection of liver diseases thereby allowing for early interventions [189]
Computed Tomography (CT) scan	MASH	• CT is a combination of X-ray technology and specialized detectors that capture the X-rays and convert them into electrical signals. Multiple X-ray projections obtained by moving detector and X-ray are then analyzed using computer algorithms to process the data and reconstruct the image information in the form of cross-sectional images • CT scan can also be used for identification of the liver steatosis, however diagnostic accuracy of CT in MASLD is very limited compared to MRI and other imaging modalities, particularly in heavily fat-laden liver and obese individuals. A study on the use of CT in the diagnosis of fatty liver in the Saudi population concluded that the plain CT scan be used as a survey tool for fatty liver disease [190]

*MASH* Metabolic dysfunction-associated steatohepatitis, *MASLD* Metabolic dysfunction-associated steatotic liver disease, *TE* Transient elastography, *MRE* Magnetic Resonance Elastography, *SWE* Shear wave elastography, *VTCE* Vibration-controlled transient elastography, *AUROC* Area Under the Receiver Operating Characteristic Curve, *CAP* Controlled attenuation parameter, *ARFI* Acoustic Radiation Force Impulse, *2D-SWE* 2D shear wave elastography, *MRE* Magnetic resonance electrography, *MRI* Magnetic resonance imaging, *PDFF* Proton density fat fraction, *MRI* Multiparametric magnetic resonance imaging, *CT* Computed Tomography

However, despite their advantages, these methods often have drawbacks. Notable drawbacks of imaging techniques include operator dependency, noninterpretable results due to obesity, ascites, availability, operational cost, and sampling variability. Perhaps most crucially, they lack sufficient sensitivity to accurately identify MASH, which is characterized by inflammation and associated with risk factors such as fibrosis cirrhosis [191]. Therefore, for a comprehensive analysis of liver health, combining the morphological and functional status of the liver with the appropriate clinical history is important.

##### Histological techniques for MASLD

Histopathological studies involve invasive techniques such as liver biopsy which involves the excision of a piece of liver or liver tissue followed by microscopic examination by a pathologist for signs of MASLD. Liver biopsy, an essential diagnostic tool for MASLD and MASH reveals distinct histological characteristics. In MASLD, steatosis is characterized by the accumulation of fat droplets in the hepatocytic cytoplasm, which can be macro or microvesicular. MASLD is also defined by the presence of steatosis in at least 5% of hepatocytes. In addition to steatosis, MASH syndrome is also characterized by hepatocellular ballooning and lobular inflammation. Ballooned hepatocytes, which indicate hepatocellular injury, lack caspase 9 and are linked to the activation of the hedgehog signaling pathway. Furthermore, Mallory-Denk bodies (MDBs), which are cytoplasmic aggregates of keratins, ubiquitin, and p62, are not unique to MASLD and can also be found in other liver diseases. Lobular necroinflammation, which is primarily composed of mononuclear cells, is prominent in Zone 3 and tends to decrease in cirrhosis. Other histological findings include enlarged mitochondria (megamitochondria), glycogenotic nuclei, and occasionally portal inflammation. Fibrosis typically begins in Zone 3 and progresses to bridging fibrosis and cirrhosis, with pediatric patients often showing periportal fibrosis initially. These histological features are crucial for diagnosing and staging MASLD and MASH, providing valuable insights into disease progression and guiding treatment strategies [192]. It is considered the “gold standard” despite its demerits such as invasiveness, sampling error, patient discomfort and pain, and limited monitoring frequency [193]. Addressing the loop limitation, early detection of MASLD significantly contributes to effective management, supported by the accuracy of findings.

##### Artificial intelligence in MASLD diagnosis (prediction, diagnosis)

Recently, artificial intelligence (AI) has emerged as an effective tool for predicting and interpreting disease risk, the presence of disease, and patient prognosis. AI is a broad field of computer science consisting of various technologies aimed at performing tasks that require human intervention and intelligence. These technologies can be categorized into machine learning, deep learning, natural learning processing (NLP), robotics, and computer vision. Machine learning, a subset of AI enables the computer to make decisions based on the identification and data of the patterns rather than using technologies. This newly developed technique is widely used in radiological imaging, clinical diagnosis, medicine, risk stratification, etc.

AI can be directly or indirectly employed for the prediction or diagnosis of MASLD. AI plays a crucial role in developing machine learning models, followed by the utilization of such machine learning models to predict disease risk which facilitates the designing of appropriate interventions for overcoming the disease.

##### Machine learning models for the analysis of reports and results

AI can also be utilized to develop tools for improving interpretations, transparency, and generalizability to increase the efficiency of clinical decision-making. For example, deep learning algorithms can enhance the automated interpretation of elastography, MRI, and CT scan results. An elaborate review on the utilization of machine learning approaches as new tools for the histopathological diagnosis of MASH and MASLD is provided elsewhere with an emphasis on the algorithms and machine learning methods utilized for analysis of histopathological results and images [194].

##### Machine learning models for MASLD risk assessment and disease prediction

Machine learning models ease statistical analysis when trained well. Thus, machine learning models can accurately predict MASLD risk, providing preliminary insights towards detailed targeted liver examinations.

An investigation by Ma et al. involving 10508 patients, explored 11 machine learning algorithms to develop a diagnostic model for MASLD. They reported that Logistic Regression (LR) achieved 83.41% accuracy, whereas support vector machine (SVM) outperformed other methods in terms of specificity (0.946) and precision (0.725), and the AODE model exhibited the highest sensitivity (0.680). By utilizing the F-measure for analysis, the Bayesian Network (BN) model demonstrated the best performance, outperforming the Fatty Liver Index (FLI) by 9.17% in F-measure score, highlighting its potential for accurate MASLD diagnosis [195].

Docherty et al*.* (2021) attempted to develop a novel machine learning model to predict MASLD via data from the NIDDK and Optum databases which consist of training an extreme gradient boosting model (XGBoost). This model resulted in a sensitivity of 81%, and a precision of 81% in predicting MASH with high accuracy [196]. A recent similar study used an AI machine learning trained XGBoost model to predict high-risk MASH via NHANES 2017- March 2020 data which achieved high sensitivity (0.82), specificity (0.91), accuracy (0.90), and AUC (0.95), outperforming traditional biomarkers such as FIB-4, APRI, BARD, and MASLD fibrosis scores [197]. A recent approach to the identification of MASLD in patients with diabetes mellitus through machine learning approaches demonstrated high performance, with success rates of correctly identifying 82.24% (815/991) and 75.00%(586/744) of MASLD ( +) and MASLD (-) patients respectively [198].

Similarly, Hassoun et al*.* recently developed NAIF (NAFLD-AI-Fibrosis), a novel AI-based tool for accurate diagnosis of advanced liver fibrosis in the general adult population, which demonstrated superior sensitivity compared with traditional scoring methods such as the APRI and Fib4 (stage F3/F4). NAIF achieved 72% precision, 61% sensitivity, and 77% specificity using data from the NHANES database [199]. Machine learning models can be trained via XGBoost to detect the risk even in the absence of a few data sets. Specifically, applying explainable AI techniques in the medical field, such as sharply additive explanations, can improve the interpretability, transparency, and generalizability of machine learning models. Such models have enormous applications in medicine, as they facilitate clinical decision-making by converting clinical data into real-world applications. While hurdles and challenges remain, the use of AI in diagnosis is promising and warrants further exploration in the future for its potential application in the medical field. Given the lack of efficient therapies for MASLD, early and accurate diagnosis is essential. Therefore, the availability of various noninvasive biomarkers and imaging techniques plays a significant role in the diagnostic process worldwide.

#### Results of the Phase 3 Trial: Spotlighting the urgent need for surrogate Biomarkers in MASLD

Resmetirom liver-targeted thyroid hormone receptor-β selective drug was recently approved by the FDA as a new drug for MASH stage of noncirrhotic patients. The drug has shown its efficacy in reducing hepatic fat content, improving fibrosis and MASH resolution, and reducing liver damage [200]. The drug has shown acceptable safety, with mild to moderate common gastrointestinal adverse events. The long-term monitoring of the effect of drug in the MASLD population is essential to determine the effect of the drug on bone, thyroid and gonadal pathology. However, the clinical evaluation of the effect of a drug relies heavily on noninvasive liver fibrosis assessments, yet current imaging techniques have several limitations, making it challenging for clinicians to make informed decisions, and highlighting the urgent need for improved diagnostic markers [201]. Hence, establishing universally accepted parameters will help maintain consistency among healthcare workers and researchers, thereby improving the quality of patient care.

## Extracellular vesicles in MASLD

### Role of EVs in MASLD

The liver, the largest highly vascularized organ of the human body, is exposed to large amounts of circulating antigens and serves as frontline immune tissue [202]. It is the primary organ responsible for removing circulating EVs, which are mainly eliminated by liver macrophages called Kupffer cells [203]. The liver comprises a heterogeneous population of cell types, including hepatocytes, cholangiocytes, hepatic stellate cells (HSCs), liver sinusoidal endothelial cells (LSECs), Kupffer cells, and a range of other immune cell populations, all of which secrete EVs [83, 204–207]. The number of EVs and molecular cargo that the individual EVs carry greatly depends upon the physiological status of the cells and is altered under disease conditions [44–48].

Recent studies have shown that liver macrophages [206, 208], hepatocytes [209, 210], and HSCs [211] are involved in the disease pathology of MASLD. The pathophysiology of MASLD is a complex process that initiates with the primary manifestation of hepatocyte cell death, followed by a substantial accumulation of inflammatory cells in the affected area. MASLD is strongly associated with obesity, elevated triglyceride levels, elevated ROS generation, oxidative DNA damage, and impaired hepatic catalase activity reflecting the failure of the antioxidant mechanism ultimately leading to *Lipoapoptosis* [212]*, Necroptosis* [213] or *Pyroptosis* [214] of hepatocytes. Oxidative stress in MASLD is often associated with elevated activation of inflammatory pathways such as the c-Jun-N-terminal kinase (JNK)/NFκB pathway [215].

Liver cells are known to release EVs both in healthy individuals and in liver patients. The pathophysiological cascade initiated by liver damage significantly alters the nature, composition, and functional properties of the EVs produced by these cells. Since EVs are involved in intercellular communication, structural damage to parent cells alters EV communication. The EVs released from the damaged liver communicate with surrounding cells and influence the microenvironment within the liver. The inflammatory cells that accumulate at the affected pathological site also cause a significant change in the EV pool by producing many inflammatory EVs in the affected area.

The earliest evidence of EVs as disease indicators came from the study of an animal model fed a high-fat diet showing a significant increase in circulating EVs. This study further provided primary evidence for the severity of steatohepatitis in mice with increased circulating EV concentrations [216]. Furthermore, apoptotic bodies released by hepatocytes increase the expression of death receptor ligands in Kupffer cells inducing apoptosis of hepatocytes leading to inflammation and fibrosis [217]. Similarly, an in vitro study revealed that the application of membrane-bound microparticles derived from murine or human hepatocytes exposed to lipotoxic stress on endothelial cells demonstrated that these particles were proangiogenic, increasing the severity of the steatohepatitis [218].

Although the role of macrophages in exacerbating disease is well known, the precise role of EVs in disease remained unclear until a study by Kakazu et al. elevated this connection. They elucidated the role of EVs in bridging these gaps. The accumulation of saturated fatty acids such as palmitate, a precursor of ceramide, a lipotoxic lipid, leads to ER stress, a commonly observed condition in diseases such as MAFLD. These EVs are enriched in C16:0 ceramide and stimulate macrophage chemotaxis via sphingosine-1-phosphate (S1P) generation. Increased levels of C16:0 ceramide-enriched circulating EVs are observed in both mice and human NASH patents, suggesting their potential as bioactive biomarkers [219]. Several such studies have shown the role of lipotoxic hepatocyte-derived EVs in aggravating inflammation. For example, a study on hypoxia in a fat-laden hepatic cell in which hypoxia-inducible factor 1-alpha (HIF-1α), was stabilized revealed a significant increase in the number of EVs released. Hypoxia-induced promoted inflammatory signals and contributed to increased EV secretion. In addition, when EVs obtained from hypoxic fat-laden tissues were used to treat Kupffer cells, there were phenotypic occurrences of hypoxic conditions in Kupffer cells suggesting the impact of EVs on disease development through crosstalk [220]. The analysis of serum extracellular vesicles by Sakane et al., 2024 revealed that the presence of proteomic signature Fibulin 3 correlated with liver-related events including MASLD and fibrosis [49].

Furthermore, MASLD is associated with elevated levels of inflammatory cytokines including IL-6 and TNF-α [221]. Elevated inflammatory cytokines influence the release and composition of hepatocyte-derived EVs [222]. As mentioned previously, these EVs can exacerbate liver inflammation and promote apoptosis in surrounding cells. The dysregulation of autophagy followed by apoptosis increases lipotoxicity. The role of EVs in autophagy in MASH is elaborately explained elsewhere [223].

Muscles and the liver play crucial regulatory roles in metabolism, working together to perform key metabolic functions such as maintaining the energy balance and regulating of glucose and lipid levels. Dysfunction in any of these genes can aggravate the other, leading to a vicious cycle of muscle and liver deterioration. MASLD is also intricately interlinked with muscle pathology, particularly sarcopenia. MASLD and sarcopenia can coexist in both obese and nonobese individuals [224, 225]. Sarcopenia and obesity-associated MASLD share common pathological manifestations including muscle loss, metabolic dysregulation, inflammation, and insulin resistance. Lean MASLD patients experience muscle wasting due to altered inflammatory and metabolic signaling. Evidence suggests that EVs contribute significantly to the deterioration of skeletal muscles in sarcopenic conditions. The molecular payloads of such EVs can exacerbate key pathological events including inflammation, protein degradation, and mitochondrial functions leading to muscle atrophy and impaired muscle regeneration [226, 227]. Compared with obesity-associated MASLD, EVs are likely to contribute to muscle wasting in non-obese MASLD through different mechanisms. EVs in obesity-associated MASLD contribute to inflammation, insulin resistance, and lipid accumulation in muscles, whereas EVs in nonobese MASLD contribute to oxidative stress, impaired metabolism, and muscle regeneration. Similar to MASLD, a significant limitation associated with sarcopenia is the lack of diagnostic markers to support clinical investigations [228]. Recognition of these profound differences in the role in disease pathology could aid in understanding disease pathology and identification of new molecular signatures for this disease.

### EVs as potential biomarkers

The accumulated evidence in the past indicates that EVs are instrumental in the pathogenesis of MASLD, underscoring their importance as biomarkers for disease evaluation. Several studies have identified EVs as promising biomarkers for MASLD.

#### TRAIL-enriched EVs

A study on how lipid-induced signaling aggravates the inflammatory response through EVs enriched with tumor necrosis factor related apoptosis-inducing ligand (TRAIL) released from hepatocytes demonstrated that lipotoxic stress induced by lipids such as palmitate and lysophosphatidylcholine (LPC) enhances EV secretion which in turn activates inflammation by inducing macrophage activation. The study also demonstrated that the inhibition of EV release ameliorates NASH in murine models. This study highlights the importance of TRAIL-enriched EVs as potential biomarkers for identifying novel therapeutic targets (inhibition of ROCK1-dependent release of EVs by hepatocytes) for NASH [229].

#### ITGβ1-enriched EVs

Monocyte-derived macrophages infiltrate the liver contributing to the inflammatory response in NASH (MASH). In 2019, Guo et al. reported that EVs enriched with Integrin Beta-1 (ITGβ1), which are released from LPC-treated hepatocytes, mediate monocyte adhesion and promote liver inflammation in a murine model of NASH. They used hepatocytes treated with either vehicle control or LPC for EV isolation and proteomic analysis. The diet-induced NASH murine model was then treated with an anti-integrin β1 (ITGβ1) neutralizing antibody (ITGβ1Ab) or a control IgG isotype. These findings suggest the presence of a new biomarker for NASH. This study also revealed that EVs derived from hepatocytes enriched with ITGβ1, regulate NASH inflammation and that antibodies against ITGβ1 ameliorate NASH in diet-induced murine models of NASH, suggesting a potential anti-inflammatory therapeutic strategy for NASH [210].

#### S1P-enriched EVs

Hepatocyte lipotoxicity leads to inflammatory macrophage effector responses during NASH. A study on EVs released from palmitic acid-treated hepatocytes revealed that they are enriched with sphingosine-1-phosphate (S1P) and are involved in recruiting macrophages to the liver. The study also demonstrated that EV S1P enrichment is largely influenced by the activity of enzymes upon sphingosine kinases 1 and 2 and that the pharmacological inhibition of these enzymes alleviated EV cargo enrichment and concomitant macrophage recruitment inferring that the sphingosine-1-phosphate (S1P) enriched EVs could be potential biomarkers and therapeutic targets for NASH [230].

#### Hepatic stellate cell (HSC)-derived EVs

Hepatic fibrosis involves excessive accumulation of extracellular matrix leading to scar formation in the liver mediated by activated HSCs under lipotoxic stress. Lipotoxic stress increases the secretion of exosomes carrying microRNAs by hepatocytes, which upon internalization can activate the proliferation and migration of HSCs. The internalization of EVs not only increases the proliferation and migration of HSCs but also affects the expression of profibrotic factors transforming growth factor-beta (TGF-β), cellular communication network 2 (CCN2), collagen type 1, and alpha-smooth muscle actin (α-SMA) [231, 232]. The isolation and characterization of such exosomes provide a new diagnostic opportunity for monitoring the progression toward fibrosis in MASLD. Similarly, recent studies on EVs secreted by healthy individuals have revealed that these EVs can inhibit progression toward fibrosis largely by alleviating the activation of HSCs or by suppressing the inflammatory pathway [233].

#### Liver sinusoidal endothelial cell (LSEC)-derived EVs

Chronic liver diseases such as nonalcoholic steatohepatitis cause fenestrated linings of liver arteries, and veins lose their discontinuity due to dedifferentiation of LSECs. LSECs form fenestrated linings in the arteries and veins of the liver. A study involving transcriptomic analysis of LSECs demonstrated that the EVs secreted by the LSECs were potent angiocrine effectors and had a deactivating effect on HSCs. The study also revealed several stage-specific proteomic signatures of EVs in chronic liver diseases revealing new therapeutic targets and potential biomarkers [234].

The accumulating literature clearly identifies tiny EVs as promising vehicles that carry enormous amounts of cellular information that could be exploited as biomarkers for MASLD. The unique characteristics of EVs and their potential advantages over existing biomarkers highlight their importance in diagnostic methods. The diagnostic potential of EVs has been clearly illustrated by a recent investigation by Jiang et al. on plasma exosomal metabolites derived from MASLD patients with impaired fasting glucose. An investigation revealed that exosomes derived from patients presented elevated levels of fatty acids, including linoleic acid, palmitate, ceramide, and oleamide, in their exosomes and reduced phosphatidylethanolamine (PE) levels. The detailed pathway analysis revealed altered linoleic acid metabolism as a characteristic feature of MASLD with impaired fasting glucose. These findings suggest the alteration in specific lipid components of EVs, clearly reflects the early metabolic dysfunction, providing valuable biomarkers for diagnosing disease progression [235].

EVs offer many advantages over existing biomarkers as they can increase the sensitivity and specificity of noninvasive diagnostics, stability, and long-term storage of EVs, enabling the development of standardized protocols and procedures for analysis. The diversity of EV molecular cargo offers a new benefit as it can potentially be utilized for multiparametric diagnostic analysis. The recent development of EV research involving the standardization of EV analysis protocols provides hope for the development of new diagnostic strategies for MASLD using EVs. Despite limitations such as lack of technical advancements for thorough analysis of EV molecular cargo, rapid progress promises a promising future for diagnostic of EVs. The swift advancements of multi-omic approaches for analyzing EVs and continuous research outputs in this domain suggest that EVs could play an essential role in next-generation diagnostic techniques offering more precise early detection of MASLD.

### Challenges in utilizing EVs for MASLD diagnostics:

#### State of EV-based diagnostics for MASLD

Studies on EV-based diagnostics are largely in the proof-of-concept phase, with most studies identifying molecular signatures through omics approaches [236]. While these studies suggest certain molecular species are expressed differently in MASLD, translating this knowledge into clinically relevant methods remains challenging.

#### Selection and isolation of EV subtypes

A key challenge in EV diagnostics is the selection and isolation of EV subtypes. EVs display heterogeneity in size, composition and function. Isolating specific subtypes of EVs is crucial, especially for the evaluation of disease specific molecular markers. For example, isolating liver-specific EVs from biological fluids is essential for accurate disease profiling in MASLD. While liver-specific markers such as Asialoglycoprotein Receptor (ASGR) protein can assist in isolating liver-specific EV populations [50], incorporating this step would add further complexity to the overall EV isolation process. EV isolation techniques such as size exclusion chromatography (SEC) and ultracentrifugation (UC) are widely used, but they have notable drawbacks [237]. SEC separates EVs into different size fractions, so choosing the right fraction is crucial. Focusing on one fraction risks missing important molecular signatures in the others. Similarly, UC can cause EV rupture, leading to the loss of molecular cargo. Even though bulk precipitation methods are cost effective, they can often introduce several other contaminants further complicating their purity and accuracy.

#### Challenges in EV data and population studies

Existing databases such as *ExoCarta* and *Vesiclepedia* provide some basic information about EVs; however, extensive population-specific information, as seen in genetic databases, is lacking. Similarly, global data on EV cargo across different geographic and ethnic populations are lacking, necessitating extensive validation for EV-based markers.

#### Knowledge gaps in EV biodistribution and circulation

Factors such as the physiological state of patients, and time and day are poorly understood. Understanding these dynamics is essential for reliable diagnosis.

#### Need for clinical validation in large cohorts

Despite of the large amount of evidence from in vitro studies, there is a need for validation of these findings in patient-derived samples to understand complex human physiology across various diverse populations before moving into clinical settings.

## Exploration of EVs for MASLD therapy

The existing therapeutic modalities for MASLD is are discussed elaborately elsewhere [238], and all the developing therapeutic modalities that are being tested in clinical trials are summarized in Table 4. The clinical trial data clearly suggest that the drugs being tested are specific for a few stages of MASLD. While most small molecules (drugs) have been developed to reduce the inflammation, fat accumulation, and scarring caused by fibrosis other interventions developed have focused on lifestyle modifications, such as diet, exercise, and probiotics. There is a need for precise stage-specific therapeutic modalities for increasing the life expectancy of individuals with MASLD. These synthetic drugs and suggested lifestyle modifications can improve the patient’s condition by slowing inflammation and preventing further accumulation of fat in the liver. In contrast, synthetic drugs do pose challenges in attaining the target that affects non-targeted cells, tissues, or organs causing unwanted side effects. Several siRNA-based therapeutic modalities have been developed for MASLD, even though they show increased efficacy, and fail to translate into the clinic. This is in part due to the failure of the delivery of therapeutic cargo to a suitable site. Similarly, the re-establishment of cellular physiology in the affected liver is possible only when the cellular components of the damaged tissue microenvironment are regenerated. Although the liver is the organ with the highest regeneration capacity, the functional retardation of the cellular components of the liver during MASLD reduces regeneration. These key challenges highlight the importance of MASLD therapeutics.

**Table 4:** Clinical trials on existing therapeutic modalities

Register No	Title	Phases	Disease	Interventions
NCT06410924	A Study to Evaluate DD01 in Overweight/Obese Subjects With MASLD/MASH	PHASE 2	MASLD/MASH	Drug: DD01/ Placebo
NCT06334666	The Efficacy of Pedometer Motivated Physical Activity for the Management of Patients with MASLD	NA	MASLD/NAFLD Metabolic Syndrome Cardiovascular Disease (CVD)	Other: Encourage using a pedometer
NCT06108219	A Phase 2b, Study Evaluating Miricorilant in Adult Patients with Nonalcoholic Steatohepatitis/Metabolic Dysfunction-Associated Steatohepatitis (MONARCH)	PHASE 2	NASH/MASH	Drug: Miricorilant/Placebo
NCT06419374	Study to Evaluate the Efficacy and Safety of Pegozafermin in Participants with Compensated Cirrhosis Due to MASH	PHASE 3	MASH/(NASH) With Compensated Cirrhosis	Biological: Pegozafern/Placebo
NCT06138327	A Study of BMN 255 in Participants with Non-Alcoholic Fatty Liver Disease and Hyperoxaluria	PHASE 1	Hyperoxaluria NAFLD Kidney Stone	Drug: BMN 255 /Placebo
NCT06121999	Behavioral Lifestyle Intervention for MASLD in adults	NA	MASLD/MASH	Behavioral: Standard of care acceptance-based behavioral weight loss program/Occupational therapy dietary and lifestyle modifications
NCT04283942	Effect of Intermittent Calorie Restriction on MASLD Patients with Abnormal Glucose Metabolism	NA	Fatty Liver Disease Type 2 Diabetes	Behavioral: Intermittent calorie restriction (ICR) / Continuous calorie restriction (CCR)
NCT05519475	A Precision Medicine Approach Using Gene Silencing to Treat a Chronic Liver Disease Called NASH in Adult Participants at Increased Genetic Risk for This Condition	PHASE 2	NASH/MASH	Drug: ALN-HSD / Placebo
NCT06359444	Effect of High-Intensity Exercise Rehabilitation on Liver Function and Insulin Sensitivity in Patients with MASLD (CENSORIAL)	NA	MASLD	Other: Combined aerobic + strength training /Combined strength + HIIT training
NCT06318169	A Study Evaluating the Efficacy and Safety of Pegozafermin in Participants with MASH and Fibrosis (ENLIGHTEN-Fibrosis)	PHASE 3	MASH/NASH With Fibrosis	Biological: Pegozafermin OTHER: Placebo
NCT06220695	A Nutrigenetic Intervention in MASLD	NA	NAFLD Fatty liver	Behavioral: Dietary intervention
NCT06373523	MASLD in Primary Hypothyroidism and Efficacy of Dapagliflozin	EARLY PHASE 1	NAFLD Hepatic Steato-Fibrosis	Drug: Dapagliflozin 10 mg0mg Tab/ Placebo/ Levothyroxine Replacement daily
NCT06461208	A Prospective Fecal Microbiota Transplantation Trial to Improve Outcomes in Patients with Cirrhosis (PROMISE)	PHASE 3	Liver Cirrhosis	Drug: Encapsulated FMT/Placebo
NCT06352697	Probiotic Lysate (Postbiotic and Metabiotic) Supplementation for Adult MASLD Patients	NA	MASLD SLD Hepatic Steatosis	Dietary supplement: Probiotic lysate (postbiotic and metabiotic) / Placebo
NCT06161571	A Study Evaluating Efruxifermin in Subjects with Noninvasively Diagnosed NASH/Metabolic Dysfunction-Associated Steatohepatitis (MASH) and (NAFLD)/Metabolic Dysfunction-Associated Steatotic Liver Disease (MASLD)	PHASE 3	NASH/MASH NAFLD/MASLD	Drug: Efruxifermin/Placebo
NCT06352177	Digital Therapeutic Lifestyle Intervention Program for Patients With MASLD	NA	NASH Liver Diseases	Other: Weight Application
NCT06047847	Determination of Biological Activity of Enriched Serum Following TOTUM-448 Consumption	NA	MASLD	Dietary supplement: TOTUM-448

*MASH* Metabolic dysfunction-associated steatohepatitis, *MASLD* Metabolic dysfunction-associated steatotic liver disease, *NAFLD* Nonalcoholic fatty liver disease, *NASH* Nonalcoholic steatohepatitis

The use of EVs as a therapeutic modality is one of the most promising strategies that is gaining importance as an answer to conquering diseases by providing an effective way of specifically treating disease alone. These membrane-bound, naturally produced lipid nanoparticles can protect the molecular cargo from degradation in the biological environment. The inherent property of EVs as cargo carriers is that they can be utilized for delivering therapeutic cargo to the site of action. The diverse range of molecular payloads, including proteins, lipids, microRNAs, and nucleic acids, is selectively, actively, or passively encapsulated in EVs, influencing their functionality. Moreover, EVs derived from stem cells inherently possess the potential to activate cellular regeneration. EVs harbor unique protein barcodes on their surface and can acquire definite bimolecular coronas depending on their surroundings which enables them to interact with the specific organ of interest or receptor of interest. Nucleic acids, chemotherapeutic drugs, small molecules, and even viruses can be essentially packed inside EVs and delivered to a targeted site. EVs are becoming therapeutic vehicles of great importance because of their biocompatibility, reduced immunogenicity, ability to cross biological barriers, versatility in cargo loading, etc. Next-generation EV therapeutics aim to utilize EVs fortified with therapeutic cargo or drugs for delivery to the target of interest to eliminate off-target effects. The inherent characteristics of EVs, including their cargo-specific therapeutic effects and site-specific actions due to protein barcoding, make them suitable candidates for developing therapeutic modalities for any disease.

EVs as therapeutic modalities can be utilized in two different ways: as delivery vehicles and as therapeutic agents. On the basis of these functions, EVs can be briefly classified into two different classes namely naturally occurring EVs and engineered EVs. Naturally occurring EVs carry endogenously packed cargoes derived from parents such as immune cells, and mesenchymal stem cells, with inherent therapeutic potential due to their origin. Whereas artificial/ engineered EVs are EVs that are altered through surface modifications through biological or chemical methods and are subjected to physical or biological treatments for loading the cargo of interest. Although there are multiple ways to load materials onto EVs, selecting a method is crucial for ensuring efficient loading. It depends on the physicochemical properties of cargoes, the source of cargo, and the EV subtype. The effectiveness of EVs as “delivery vehicles” and “therapeutic agents” is diversely supported by studies of different diseases.

EVs naturally produced by cells harbor enormous amounts of cellular components that increase their therapeutic value. The majority of MSC-derived EVs are being therapeutically employed for their potential immunomodulation or immunoregulation and regeneration. As of 2024, there are a dozen clinical trials ongoing in which EVs derived from MSCs have been employed for immune regulation and regenerative medicine. The therapeutic utility of MSC-derived EVs has been reviewed in detail elsewhere with relevant ongoing clinical trials [239]. The role of MSC-derived EVs as therapeutic agents in treating liver diseases is being thoroughly investigated, and the clinical trials registered to employ EVs for liver-related disorders are listed in Table 5.

**Table 5:** The role of MSC-derived EVs as therapeutic agents in treating liver diseases

Register No	Title	Phase	Status	Condition	Intervention
NCT05940610	The Safety and Efficacy of MSC-EVs in Acute/​Acute-on-Chronic Liver Failure	Phase1/ Phase 2	Withdrawn	Acute-on-chronic liver failure	MSC-EVs
NCT05881668	MSC-EV in Acute-on-Chronic Liver Failure After Liver Transplantation	Phase1	Withdrawn	Acute-on-chronic liver failure after Liver Transplantation	MSC-EVs
NCT05871463	Effect of Mesenchymal Stem Cells-derived Exosomes in Decompensated Liver Cirrhosis	Phase 2	Recruiting	Decompensated liver cirrhosis	MSC-derived exosomes

*MSC-EVs* Mesenchymal stem cell derived extracellular vesicles

### EVs as potential therapeutic agents for MASLD

Although there is limited evidence for EVs as therapeutic modalities for treating MASLD, the accumulating evidence on the utilization of EVs for therapeutics and delivery systems in several liver diseases and in vitro models resembling MASLD suggests the scope of EV therapy for MASLD.

#### EVs in Hepatic steatosis and Inflammation therapy

##### MSC-derived EVs for Hepatic steatosis and Inflammation

Mesenchymal stem cell-derived EVs from various stem cell sources have shown anti-inflammatory and regenerative effects can be utilized as a therapeutic strategy for treating hepatic steatosis and inflammation. Hepatic steatosis and inflammation are the key pathological events occurring in the initial stages of MASLD, progressing toward severe stages such as fibrosis. Several studies have been conducted to assess the ability of MSC-derived EVs to ameliorate hepatic steatosis and inflammation.

As described in the previous sections, EVs derive their molecular cargo and functionality from parent cells. A study exploring the therapeutic utility of microRNA-136-5p in EVs derived from mice bone marrow-derived mesenchymal cells demonstrated that the inhibition of GNAS/STAT3 signaling pathway and lipopolysaccharide (LPS)-induced inflammation resulting in reduced liver inflammation and enhanced M2 macrophage polarization through the GNAS-mediated PI3K/ERK/STAT3 axis in an animal model of chronic liver damage induced by carbon tetrachloride [240].

A similar study on exosomes derived from human umbilical cord-mesenchymal stem cells (hUC-MSCs) demonstrated their efficacy in mitigating this disease. This study emphasized the influence of MSC-derived exosomal miR-24-3p on reducing lipid accumulation, oxidative stress, and inflammation, leading to improved hepatic function and decreased steatosis in both palmitate-treated mouse hepatocytes in vitro and a high-fat diet-induced NAFLD mouse model *invivo*. miR-24-3p exerts these protective effects by targeting Kelch-like ECH-associated protein 1 (KEAP-1) signaling, thereby attenuating hepatic lipid metabolism disturbances, inflammation, and oxidative stress [241].

A study on the direct use of human umbilical cord mesenchymal stem cell (hUC-MSC)-derived exosomes for their therapeutic potential in nonalcoholic steatohepatitis (NASH) using an MCD-induced mouse model revealed that the intravenous transplantation of hUC-MSC exosomes improved body weight loss and liver damage induced by MCD in mice. Furthermore, it also reduced inflammatory cytokines in liver tissue and induced anti-inflammatory phenotypes in macrophages. Macrophage polarization was evident in both in vitro and in vivo experimental models. Exosomes were also capable of reversing the downregulation of PPARα protein expression in ox-LDL-treated hepatocytes in vitro and in vivo in NASH mouse livers [242].

The potential application of human placenta-derived MSC extracellular vesicles (hPMSC-EVs) in liver regeneration following hepatectomy was investigated in 2022 by Li et al. Intravenously administered hPMSC-EVs before partial hepatectomy could potentially improve liver regeneration in vivo and hepatocyte proliferation in vitro. These findings suggest that hPMSCs-EVs have the potential to prevent hepatic dysfunction and improve liver regeneration, possibly through circ-RBM23 delivery [243]. Similarly, a study on the delivery of inherent RNF31 through EVs derived from mesenchymal stem cells revealed that RNF31 delivery significantly improved liver function by alleviating hepatic steatosis in high-fat diet-fed mice [244].

#### Non-MSC EVs for hepatic steatosis and inflammation

Literature evidence suggests that breastfeeding reduces the risk of MASLD/NAFLD. A study on the effects of EVs derived from mothers’ milk on NAFLD model mice and primary hepatocytes treated with free fatty acid showed that the breast milk-derived EVs alleviated hepatic steatosis and insulin resistance in NAFLD mice by inhibiting lipogenesis and promoting lipolysis. These effects are likely due to EV cargo (proteins and miRNAs) related to lipid metabolism, suggesting a new therapeutic strategy for NAFLD treatment [245]. Exosomes derived from stem cells of the apical papilla (SCAPs) have also shown significant therapeutic potential for treating nonalcoholic steatohepatitis (NASH) in a methionine-choline deficient (MCD) diet-induced mouse model. A study on these exosomes demonstrated that the administration of SCAP-derived exosomes led to reduced liver damage and hepatic fat accumulation and improved lipid metabolism through the upregulation of p-AMPK and mitochondrial biogenesis factors [246].

Immune cells play a key regulatory role in exhibiting the immune cascade. Macrophage polarization is important in inflammatory and anti-inflammatory reactions. M2 macrophage-derived exosomes loaded with siRNA targeting RIPK3 significantly reduced pro-inflammatory cytokines, improved liver pathology, and balanced Th17/Treg cell ratios in a mouse model of immune hepatitis. These findings suggest that EVs can effectively deliver therapeutic agents to liver cells, suggesting a potential strategy for treating NAFLD [247].

#### EVs in fibrosis therapy

##### MSC derived EVs for fibrosis

EVs, a paracrine effector of MSCs, can be employed to regenerate the hepatic cell population for recovery from fibrosis. MSCs have already been explored as a therapeutic modality for various fibrotic conditions, including pulmonary fibrosis, spinal cord injury, scarring, and organ transplantation. Recent studies on the use of MSC-derived EVs in treatment of liver fibrosis have revealed that MSC-derived EVs as are potential therapeutic agents for liver fibrosis. A study on the efficacy of human AMSCs amniotic mesenchymal stem cell-derived extracellular vesicles (AMSC-EVs) in treating hepatic fibrosis revealed that AMSC-EVs delivered miR-200a into hepatocytes suppressing ZEB1/PIK3R3 axis. Suppression of the ZEB1/PIK3R3 axis reduces hepatic fibrosis by inhibiting its antifibrotic- effect [248].

A similar study explored the utilization of Wharton's jelly mesenchymal stem cell (hWJMSC-Exo)—derived exosomes for improving liver function and regeneration during liver fibrosis. This study demonstrated that delivering miR-124 via exosomes from human Wharton's jelly mesenchymal stem cells (hWJMSC-Exos) improved liver fibrosis. Exosomes enriched with miR-124 could significantly reduce inflammation and collagen accumulation. The levels of the fibrotic inflammatory markers IL-6, IL-17, TGF-β, STAT3, α-SMA, and COL in a CCl4-induced mouse model, were significantly decreased the administration of miR-124-enriched exosomes (ExomiR-124). The study also demonstrated that ExomiR-124 also promoted the shift of splenic monocytes from inflammatory to restorative phenotypes, confirming that ExomiR-124 is a promising antiinflammatory and antifibrotic therapeutic option for liver fibrosis [249].

A study investigating the therapeutic efficacy of MSC-derived exosome and medical ozone in a CCL_4_-induced liver fibrosis rat model revealed superior efficacy of EVs over ozone in reducing liver enzyme levels, oxidative stress markers, and histological liver damage. The evidence given by the study highlights the therapeutic potential of MSC-MVs and the authors also conclude that future research is needed to optimize dosages and explore combined therapies for enhanced effectiveness [250].

The antifibrotic effect of human-derived EVs was supported by another study in which EV-derived human liver stem cells (HLSCs) were evaluated for their ability to treat NASH, a stage in the MASLD spectrum in immunocompromised mice. EV treatment significantly reduces signs of liver fibrosis and inflammation and downregulates 28 out of 29 fibrosis-associated genes upregulated in the NASH liver [251]. The use of human placental mesenchymal stem cell-derived exosomes (ExoMSC) employed for treating liver fibrosis showed an effective reduction of fibrosis and improved the liver microenvironment in a PSC mouse model and patient-derived organoids autoimmune diseases by inhibiting Th17 differentiation and reducing ER stress. ExoMSCs can downregulate IκBζ expression and PERK/CHOP signaling, suggesting potential therapeutic applications of ExoMSC for PSC and Th17-related liver diseases [252].

##### Non-MSC-derived EVs in fibrosis

Semaglutide, a GLP-1 receptor agonist, shows promise in treating MASLD associated with T2D by modulating the exosome composition. In a study with T2D patients, responders to semaglutide treatment exhibited significant improvements in liver fibrosis markers, as their exosomes reduced stellate cell activation and fibrosis-related protein expression. These findings suggest that semaglutide has therapeutic potential through indirect mechanisms involving exosome-mediated cell signaling, although the exact pathways involved further investigation. Although exosomes or EVs are not directly employed for therapy, the findings underscore the importance of exosome research in the development of MASLD therapies [253].

The therapeutic potential of curcumin is hampered by its hydrophobicity and low bioavailability. Owing to their favorable size, composition, and non-immunogenic properties, small Extracellular Vesicles (sEVs) offer a promising solution for curcumin delivery. This study investigates curcumin-loaded milk sEVs, via passive and active (saponin-assisted) loading methods, which maintain nanoparticle integrity and size. Active loading resulted in significantly increased curcumin encapsulation. In vitro tests revealed greater cytotoxicity in cancer cells versus primary hepatocytes, whereas in vivo studies demonstrated reduced liver damage and fibrosis in a liver fibrosis model, highlighting sEVs as effective curcumin delivery systems [254].

### Advances in EV therapy for MASLD

#### Targeted delivery for MASLD therapy

Surface decoration of EVs alters the EV uptake by cells, which can be utilized for targeting EVs at specific sites. EVs can be surface engineered to achieve specific targeting. A detailed review of how extracellular vesicles target nonparenchymal cells to address liver fibrosis is given elsewhere [255]. EVs decorated with ligands of interest or targeting moieties produced either by direct modification or parent cell modification are utilized for targeting specific receptors, cells, or tissues. Notably, several preclinical evidence experimentally demonstrated the importance of targeting extracellular vesicles for the precise delivery of therapeutic cargo, eliminating off-target effects and enhancing the efficacy of therapy.

A study on the attenuation of hepatic steatosis by Yu et al. employed a unique targeting strategy for delivering pirfenidone-laden EVs to HSCs. This study demonstrated that the HA-modified EVs carrying pirfenidone to HSCs showed superior efficacy in inhibiting HSC activation and reducing collagen synthesis in both the rat HSC-T6 and the BRL cell lines. Furthermore, a previous study also demonstrated that in a murine hepatic fibrosis model, therapeutic strategy could significantly improve hepatic cell morphology and ameliorate hepatic fibrosis [256]. Similarly, the HSTP1 peptide fused with exosomal membrane protein Lamp2b through genetic engineering was employed to target the HSCs for effective reversal of fibrosis via human umbilical cord mesenchymal stem cell (Huc-MSC)-derived exosomes [257].

In a similar study EVs from human adipose-derived stem cells (ADSCs) were exogenously modified to bear vitamin A on their surface and showed enhanced targeting towards HSCs. Compared with bare EVs, exogenously modified EVs can reverse the fibrotic cascade even at tenfold lower doses [258].

The screening of targeting peptides, ligands, or chemical compounds to evaluate their efficacy and specificity provides the ideal candidates for targeting a particular receptor. This ideal targeting moiety can be further employed to target specific hepatic cellular niches. The knowledge about MASLD pathology provides essential grounds for the identification of a suitable cellular population to be targeted for the effective reversal of the disease. Future EV therapeutics rely on the development of essential loading strategies for loading molecular cargo and targeting EVs to the suitable hepatic cellular niche. Advancements in the targeting and loading strategies essentially enhance the therapeutic potential of EVs for overcoming MASLD.

#### Hybrid EVs for MASLD therapy

The efficacy of EVs can also be enhanced by coupling EVs with other nanocarriers to increase their therapeutic potential. The internalization of EVs is generally driven by stereochemical factors of the cell membrane. Anionic components of cellular membranes often offer electrostatic repulsion toward exosomes, affecting EV internalization. Sato et al. developed a biological nano transporter hybrid via the fusion of liposomes and EVs. The hybrid biological nano transporter aided with PEG surface modifications improved interaction with the target cells facilitating efficient delivery of the therapeutic cargo [259]. Similarly, Piffoux et al. and Mukherjee et al*.* demonstrated enhanced drug delivery efficiency by mixing MSC-derived EVs with PEGylated liposomes, suggesting a promising strategy for improving drug delivery systems [260, 261]. Evers et al. compared the physicochemical properties and functionality of liposomes with those of hybrid nanoparticles. An effective delivery vehicle was created by fusing engineered EVs with liposomes, which exhibited greater efficacy than liposomes alone [262]. Similar studies on hybrid EVs revealed that the combination of EVs with other nanocarriers can increase the efficacy of carrier systems. However, EVs also pose several hurdles as efficient cargo carriers; stability issues and the natural heterogeneity of EVs, reproducibility, and consistency make accessing this carrier system in the clinic difficult. However, when these limitations are properly addressed, a nanocarrier system coupled with surface-engineered EVs for efficient targeting, when utilized, can effectively enhance therapeutic potential.

### Challenges in EV therapeutics

In conclusion, EVs are emerging therapeutic measures for MASLD both as cargo carriers and therapeutic agents. However, emerging EV therapeutics pose considerable challenges for translation into the clinic. The major challenge associated with EV therapeutics is maintaining the consistency of the therapeutic products, as they are biogenic in origin. The heterogeneity of the EV population also creates the need for efficient isolation of clinical-grade EV subpopulations. Producing high-quality EVs with consistent properties remains a significant hurdle, as scalable manufacturing and reproducible manufacturing processes are needed.

The significant strides in improving bioengineering EVs in the past 5 years and efforts to optimize the manufacturing of EVs for clinics are poised to overcome current challenges paving the way for a reliable EV-based therapy for MASLD. The current trend and rapidity in EV research ensure that these challenges will soon be addressed, resulting in the development of effective EV-based therapeutic measures for MASLD. This rapid progress holds promise for significantly improving patient outcomes by providing precise, targeted, and efficient therapeutic strategies for MASLD.

## Strengths and limitations

This review consolidates a substantial body of research carried out in the past on EVs and MASLD, making it a literature resource for clinicians and researchers. Key topics regarding EVs and MASLD are simplified with the help of tables and figures to easily grasp the interaction of EVs in MASLD. This study included multiple clinical trials and the concerning issues surrounding MASLD for validating the claims and ensuring their timely relevance. The comprehensive tables aid in the practical application of this knowledge in clinical settings. By presenting both the positive and negative aspects of EVs in diagnostics and therapeutics without bias, this review provides an objective overview of the current state of research, which will help researchers identify existing knowledge gaps and potential areas for future investigations.

This review provides a broad perspective on the disease pathology of MASLD, along with other liver diseases, addressing clinical trials and the role of EVs in disease manifestation. Owing to the limited availability of EV-related studies specific to MASLD in the current literature, other liver diseases with overlapping pathological features were also explored. This approach is based on the rationale that MASLD shares several similar pathological events with other liver diseases, allowing insights into the potential utility of EVs in MASLD by analogy. However, a more focused bioinformatic study would help synthesize precise evidence regarding EV-based diagnostic markers specifically for MASLD.

## Conclusion and future prospective

EVs are one such newly explored research field of science with continuous evolution where there has been significant research ongoing in the last 20 years, peaking in 2016 with 20.47% of the total articles from the entire period [263], which is growing rapidly with much wider and more practical applications for systematic problems of the population. The use of EVs as a therapeutic modality has also gained significant attention, as demonstrated by bibliometric data. This is evident from the notable increase in research, particularly the use of exosomes as delivery vehicles, which has grown at an impressive annual rate of 55.8% since 2013 [264]. A noteworthy number of patents were generated, and the number of grants sanctioned almost doubled in 2016 [263]. As the research digs the well of knowledge, the field delves deeper, and a vast area remains to explore. Earlier, the major focus was on EV-related biofluids and cell types. With the growth, the research has focused on the role of these EVs in disease diagnosis and therapy. Current research focuses on the exploration of disease through advanced technologies such as multiomics analysis with the assistance of bioinformatics analysis, thus providing accurate resolutions.

In the future, EV research should focus predominantly on two aspects. First, there is a need for the development of an EV-based rapid disease detection kit that can isolate and characterize organ-specific EVs from circulating biofluids to ascertain the presence of disease at a specific site in the body. Second, there is a need for a device that can accurately characterize the organ-specific EVs through multi-omics approaches, providing detailed insights into the incidence of the disease by analyzing risk factors, pathogenesis, progression of the disease, and severity of the disease along mortality predictions.

Therapeutically, the EV research field is concerned with the development of EVs as efficient therapeutic agents and cargo carriers. Research should focus soon on understanding the mechanism of the components of EVs that are effectively improving the disease, which can be implemented to improve the efficiency of EVs as therapeutic agents more precisely. The large-scale manufacturing of therapeutic EVs involving selectively enriching the effector EV subpopulation would be the immediate aim of EV therapeutics. Furthermore, EV-based research toward standardized platform development can enable accurate delivery of the cargo of therapeutic importance to the targeted site more effectively and efficiently, and improving the disease condition is needed to enhance and extend targeted delivery via EVs. Synergistic approaches to solve or select targeting moieties via omics and bioinformatics and efficient engineering techniques for expressing the targeting moiety along with an EV cargo loading strategy would efficiently pave the way for the use of EVs as therapeutic carriers in the clinic.

The paradigm is shifting toward translational applications in EV research, from identifying the existence of these lipid-layered nanoparticles, and the utilization of EVs as next-generation drug carriers. Among several lifestyle disorders, MASLD is one such silent disease prevailing worldwide due to metabolic syndrome in most of the population. As prominent particles of intercellular communication, through the development of EV-based devices, they can make one of the minimally invasive methods much more efficient in conveying the mechanism of disease progression along with the severity and efficient therapeutic utility. As explore trends in EV research is investigated, they can become “EV” erything for MASLD.

## Supplementary Information

Supplementary Material 1.

Supplementary Material 2.

Supplementary Material 3.

Supplementary Material 4.

Supplementary Material 5.

## Data Availability

No datasets were generated or analysed during the current study.

## References

[1] Sharma S, Matheson A, Lambrick D, Faulkner J, Lounsbury DW, Vaidya A, et al. Dietary practices, physical activity and social determinants of non-communicable diseases in Nepal: A systemic analysis. PLoS ONE. 2023;18(2):e0281355. 36745612 10.1371/journal.pone.0281355 PMC9901760

[2] Wu J, Fu Y, Chen D, Zhang H, Xue E, Shao J, et al. Sedentary behavior patterns and the risk of non-communicable diseases and all-cause mortality: A systematic review and meta-analysis. Int J Nurs Stud. 2023;1(146):104563. 10.1016/j.ijnurstu.2023.104563 37523952

[3] Nurwanti E, Uddin M, Chang JS, Hadi H, Syed-Abdul S, Su ECY, et al. Roles of Sedentary Behaviors and Unhealthy Foods in Increasing the Obesity Risk in Adult Men and Women: A Cross-Sectional National Study. Nutrients. 2018;10(6):704. 29857537 10.3390/nu10060704 PMC6024814

[4] Hamilton MT, Hamilton DG, Zderic TW. Sedentary behavior as a mediator of type 2 diabetes. Med Sport Sci. 2014;60:11–26. 25226797 10.1159/000357332 PMC4364419

[5] Kakamu T, Hidaka T, Kumagai T, Masuishi Y, Kasuga H, Endo S, et al. Unhealthy changes in eating habits cause acute onset hypertension in the normotensive community-dwelling elderly-3 years cohort study. Medicine (Baltimore). 2019;98(15):e15071. 30985658 10.1097/MD.0000000000015071 PMC6485880

[6] Enani S, Bahijri S, Malibary M, Jambi H, Eldakhakhny B, Al-Ahmadi J, et al. The Association between Dyslipidemia, Dietary Habits and Other Lifestyle Indicators among Non-Diabetic Attendees of Primary Health Care Centers in Jeddah, Saudi Arabia. Nutrients. 2020;12(8):2441. 32823801 10.3390/nu12082441 PMC7469008

[7] Wilmot EG, Edwardson CL, Achana FA, Davies MJ, Gorely T, Gray LJ, et al. Sedentary time in adults and the association with diabetes, cardiovascular disease and death: systematic review and meta-analysis. Diabetologia. 2012;55(11):2895–905. 22890825 10.1007/s00125-012-2677-z

[8] Vajdi M, Karimi A, Farhangi MA, Ardekani AM. The association between healthy lifestyle score and risk of metabolic syndrome in Iranian adults: a cross-sectional study. BMC Endocr Disord. 2023;23(1):16. 36647030 10.1186/s12902-023-01270-0 PMC9843981

[9] Park JH, Moon JH, Kim HJ, Kong MH, Oh YH. Sedentary Lifestyle: Overview of Updated Evidence of Potential Health Risks. Korean J Fam Med. 2020;41(6):365–73. 33242381 10.4082/kjfm.20.0165 PMC7700832

[10] Jeong SH, Jang BN, Kim SH, Kim GR, Park EC, Jang SI. Association between sedentary time and sleep quality based on the Pittsburgh Sleep Quality Index among South Korean adults. BMC Public Health. 2021;15(21):2290. 10.1186/s12889-021-12388-y PMC8675446 34911512

[11] Li Y, Pan A, Wang DD, Liu X, Dhana K, Franco OH, et al. The Impact of Healthy Lifestyle Factors on Life Expectancies in the US population. Circulation. 2018;138(4):345–55. 29712712 10.1161/CIRCULATIONAHA.117.032047 PMC6207481

[12] Chaker L, Falla A, van der Lee SJ, Muka T, Imo D, Jaspers L, et al. The global impact of non-communicable diseases on macro-economic productivity: a systematic review. Eur J Epidemiol. 2015;30(5):357–95. 25837965 10.1007/s10654-015-0026-5 PMC4457808

[13] Farhud DD. Impact of Lifestyle on Health. Iran J Public Health. 2015;44(11):1442–4. 26744700 PMC4703222

[14] Hydes T, Alam U, Cuthbertson DJ. The Impact of Macronutrient Intake on Non-alcoholic Fatty Liver Disease (NAFLD): Too Much Fat, Too Much Carbohydrate, or Just Too Many Calories? Front Nutr. 2021;8:640557. 33665203 10.3389/fnut.2021.640557 PMC7921724

[15] Rector RS, Thyfault JP. Does physical inactivity cause nonalcoholic fatty liver disease? J Appl Physiol Bethesda Md 1985. 2011;111(6):1828–35. 10.1152/japplphysiol.00384.2011 21565984

[16] Zhang X, Chen K, Yin S, Qian M, Liu C. Association of leisure sedentary behavior and physical activity with the risk of nonalcoholic fatty liver disease: a two-sample Mendelian randomization study. Front Nutr. 2023;9(10):1158810. 10.3389/fnut.2023.1158810 PMC10289033 37360298

[17] Gerges SH, Wahdan SA, Elsherbiny DA, El-Demerdash E. Non-alcoholic fatty liver disease: An overview of risk factors, pathophysiological mechanisms, diagnostic procedures, and therapeutic interventions. Life Sci. 2021;15(271):119220. 10.1016/j.lfs.2021.119220 33592199

[18] El-Aziz MKA, Dawoud A, Kiriacos CJ, Fahmy SA, Hamdy NM, Youness RA. Decoding hepatocarcinogenesis from a noncoding RNAs perspective. J Cell Physiol. 2023;238(9):1982–2009. 37450612 10.1002/jcp.31076

[19] Rinella ME, Lazarus JV, Ratziu V, Francque SM, Sanyal AJ, Kanwal F, et al. A multisociety Delphi consensus statement on new fatty liver disease nomenclature. J Hepatol. 2023;79(6):1542–56. 37364790 10.1016/j.jhep.2023.06.003

[20] Riazi K, Azhari H, Charette JH, Underwood FE, King JA, Afshar EE, et al. The prevalence and incidence of NAFLD worldwide: a systematic review and meta-analysis. Lancet Gastroenterol Hepatol. 2022;7(9):851–61. 35798021 10.1016/S2468-1253(22)00165-0

[21] Simon TG, Roelstraete B, Khalili H, Hagström H, Ludvigsson JF. Mortality in Biopsy-Confirmed Nonalcoholic Fatty Liver Disease. Gut. 2021;70(7):1375–82. 33037056 10.1136/gutjnl-2020-322786 PMC8185553

[22] Lazarus JV, Colombo M, Cortez-Pinto H, Huang TTK, Miller V, Ninburg M, et al. NAFLD - sounding the alarm on a silent epidemic. Nat Rev Gastroenterol Hepatol. 2020;17(7):377–9. 32514153 10.1038/s41575-020-0315-7

[23] Thampanitchawong P, Piratvisuth T. Liver biopsy:complications and risk factors. World J Gastroenterol. 1999;5(4):301–4. 11819452 10.3748/wjg.v5.i4.301 PMC4695539

[24] Machado MV, Cortez-Pinto H. Non-invasive diagnosis of non-alcoholic fatty liver disease. A critical appraisal. J Hepatol. 2013;58(5):1007–19. 23183525 10.1016/j.jhep.2012.11.021

[25] Wan Y, Wang D, Li H, Xu Y. The imaging techniques and diagnostic performance of ultrasound, CT, and MRI in detecting liver steatosis and fat quantification: A systematic review. J Radiat Res Appl Sci. 2023;16(4):100658.

[26] Shang M, Ji JS, Song C, Gao BJ, Jin JG, Kuo WP, et al. Extracellular Vesicles: A Brief Overview and Its Role in Precision Medicine. Methods Mol Biol Clifton NJ. 2017;1660:1–14. 10.1007/978-1-4939-7253-1_1 28828643

[27] Mahmoud MM, Sanad EF, Elshimy RAA, Hamdy NM. Competitive Endogenous Role of the LINC00511/miR-185-3p Axis and miR-301a-3p From Liquid Biopsy as Molecular Markers for Breast Cancer Diagnosis. Front Oncol. 2021;20(11):749753. 10.3389/fonc.2021.749753 PMC8567754 34745973

[28] Ferguson S, Yang KS, Weissleder R. Single extracellular vesicle analysis for early cancer detection. Trends Mol Med. 2022;28(8):681–92. 35624008 10.1016/j.molmed.2022.05.003 PMC9339504

[29] Hinestrosa JP, Kurzrock R, Lewis JM, Schork NJ, Schroeder G, Kamat AM, et al. Early-stage multi-cancer detection using an extracellular vesicle protein-based blood test. Commun Med. 2022;2(1):1–9. 35603292 10.1038/s43856-022-00088-6 PMC9053211

[30] Liu SY, Liao Y, Hosseinifard H, Imani S, Wen QL. Diagnostic Role of Extracellular Vesicles in Cancer: A Comprehensive Systematic Review and Meta-Analysis. Front Cell Dev Biol. 2021;9:705791. 34722499 10.3389/fcell.2021.705791 PMC8555429

[31] Lawrence R, Watters M, Davies CR, Pantel K, Lu YJ. Circulating tumour cells for early detection of clinically relevant cancer. Nat Rev Clin Oncol. 2023;20(7):487–500. 37268719 10.1038/s41571-023-00781-y PMC10237083

[32] Kowal-Wisniewska E, Jaskiewicz K, Bartochowska A, Kiwerska K, Ustaszewski A, Gorecki T, et al. Towards effectiveness of cell free DNA based liquid biopsy in head and neck squamous cell carcinoma. Sci Rep. 2024;14(1):2251. 38278927 10.1038/s41598-024-52031-5 PMC10817923

[33] Lone SN, Nisar S, Masoodi T, Singh M, Rizwan A, Hashem S, et al. Liquid biopsy: a step closer to transform diagnosis, prognosis and future of cancer treatments. Mol Cancer. 2022;21(1):79. 35303879 10.1186/s12943-022-01543-7 PMC8932066

[34] Yu J, Sane S, Kim JE, Yun S, Kim HJ, Jo KB, et al. Biogenesis and delivery of extracellular vesicles: harnessing the power of EVs for diagnostics and therapeutics. Front Mol Biosci. 2023;10:1330400. 38234582 10.3389/fmolb.2023.1330400 PMC10791869

[35] Arteaga-Blanco LA, Evans AE, Dixon DA. Plasma-Derived Extracellular Vesicles and Non-Extracellular Vesicle Components from APCMin/+ Mice Promote Pro-Tumorigenic Activities and Activate Human Colonic Fibroblasts via the NF-κB Signaling Pathway. Cells. 2024;13(14):1195. 39056778 10.3390/cells13141195 PMC11274984

[36] Welsh JA, Goberdhan DCI, O’Driscoll L, Buzas EI, Blenkiron C, Bussolati B, et al. Minimal information for studies of extracellular vesicles (MISEV2023): From basic to advanced approaches. J Extracell Vesicles. 2024;13(2):e12404. 38326288 10.1002/jev2.12404 PMC10850029

[37] Berumen Sánchez G, Bunn KE, Pua HH, Rafat M. Extracellular vesicles: mediators of intercellular communication in tissue injury and disease. Cell Commun Signal. 2021;19(1):104. 34656117 10.1186/s12964-021-00787-y PMC8520651

[38] van Niel G, D’Angelo G, Raposo G. Shedding light on the cell biology of extracellular vesicles. Nat Rev Mol Cell Biol. 2018;19(4):213–28. 29339798 10.1038/nrm.2017.125

[39] Hallal S, Tűzesi Á, Grau GE, Buckland ME, Alexander KL. Understanding the extracellular vesicle surface for clinical molecular biology. J Extracell Vesicles. 2022;11(10):e12260. 36239734 10.1002/jev2.12260 PMC9563386

[40] Chatterjee M, Özdemir S, Fritz C, Möbius W, Kleineidam L, Mandelkow E, et al. Plasma extracellular vesicle tau and TDP-43 as diagnostic biomarkers in FTD and ALS. Nat Med. 2024;30(6):1771–83. 38890531 10.1038/s41591-024-02937-4 PMC11186765

[41] Du S, Zhao Y, Lv C, Wei M, Gao Z, Meng X. Applying Serum Proteins and MicroRNA as Novel Biomarkers for Early-Stage Cervical Cancer Detection. Sci Rep. 2020;10(1):9033. 32493989 10.1038/s41598-020-65850-z PMC7271168

[42] Gámez-Valero A, Lozano-Ramos SI, Bancu I, Lauzurica-Valdemoros R, Borràs FE. Urinary extracellular vesicles as source of biomarkers in kidney diseases. Front Immunol. 2015;6:6. 25688242 10.3389/fimmu.2015.00006 PMC4311634

[43] Roy S, Kashyap NN, Anchan AS, Punja D, Jasti DB, Upadhya D. Urinary extracellular vesicle dynamics in Parkinson’s disease patients with urinary dysfunction. Front Neurol. 2023;14:1250832. 38046591 10.3389/fneur.2023.1250832 PMC10691254

[44] Sjoqvist S, Otake K. A pilot study using proximity extension assay of cerebrospinal fluid and its extracellular vesicles identifies novel amyotrophic lateral sclerosis biomarker candidates. Biochem Biophys Res Commun. 2022;12(613):166–73. 10.1016/j.bbrc.2022.04.127 35567903

[45] Sjoqvist S, Otake K. Saliva and Saliva Extracellular Vesicles for Biomarker Candidate Identification—Assay Development and Pilot Study in Amyotrophic Lateral Sclerosis. Int J Mol Sci. 2023;24(6):5237. 36982312 10.3390/ijms24065237 PMC10049503

[46] Cui L, Zheng J, Lu Y, Lin P, Lin Y, Zheng Y, et al. New frontiers in salivary extracellular vesicles: transforming diagnostics, monitoring, and therapeutics in oral and systemic diseases. J Nanobiotechnology. 2024;22(1):171. 38610017 10.1186/s12951-024-02443-2 PMC11015696

[47] Mir B, Goettsch C. Extracellular Vesicles as Delivery Vehicles of Specific Cellular Cargo. Cells. 2020;9(7):1601. 32630649 10.3390/cells9071601 PMC7407641

[48] Fabbiano F, Corsi J, Gurrieri E, Trevisan C, Notarangelo M, D’Agostino VG. RNA packaging into extracellular vesicles: An orchestra of RNA-binding proteins? J Extracell Vesicles. 2020;10(2):e12043. 33391635 10.1002/jev2.12043 PMC7769857

[49] Sakane S, Hikita H, Shirai K, Sakamoto T, Narumi R, Adachi J, et al. Proteomic analysis of serum extracellular vesicles reveals Fibulin-3 as a new marker predicting liver-related events in MASLD. Hepatol Commun. 2024;8(6):e0448. 38829196 10.1097/HC9.0000000000000448 PMC11150025

[50] Muñoz-Hernández R, Rojas Á, Gato S, Gallego J, Gil-Gómez A, Castro MJ, et al. Extracellular Vesicles as Biomarkers in Liver Disease. Int J Mol Sci. 2022;23(24):16217. 36555854 10.3390/ijms232416217 PMC9786586

[51] Cho YE, Im EJ, Moon PG, Mezey E, Song BJ, Baek MC. Increased liver-specific proteins in circulating extracellular vesicles as potential biomarkers for drug- and alcohol-induced liver injury. PLoS ONE. 2017;12(2):e0172463. 28225807 10.1371/journal.pone.0172463 PMC5321292

[52] Hu M, You Z, Li Y, Huang B, Cui N, Wang R, et al. Serum Biomarkers for Autoimmune Hepatitis Type 1: the Case for CD48 and a Review of the Literature. Clin Rev Allergy Immunol. 2022;63(3):342–56. 35657576 10.1007/s12016-022-08935-z

[53] Juratli MA, Pollmann NS, Oppermann E, Mohr A, Roy D, Schnitzbauer A, et al. Extracellular vesicles as potential biomarkers for diagnosis and recurrence detection of hepatocellular carcinoma. Sci Rep. 2024;14(1):5322. 38438456 10.1038/s41598-024-55888-8 PMC10912302

[54] Lim HK, Jeffrey GP, Ramm GA, Soekmadji C. Pathogenesis of Viral Hepatitis-Induced Chronic Liver Disease: Role of Extracellular Vesicles. Front Cell Infect Microbiol. 2020;10(10):587628. 33240824 10.3389/fcimb.2020.587628 PMC7683521

[55] Chargaff E, West R. THE BIOLOGICAL SIGNIFICANCE OF THE THROMBOPLASTIC PROTEIN OF BLOOD. J Biol Chem. 1946;166(1):189–97. 20273687

[56] Di Bella MA. Overview and Update on Extracellular Vesicles: Considerations on Exosomes and Their Application in Modern Medicine. Biology. 2022;11(6):804. 35741325 10.3390/biology11060804 PMC9220244

[57] Minciacchi VR, Freeman MR, Di Vizio D. Extracellular Vesicles in Cancer: Exosomes, Microvesicles and the Emerging Role of Large Oncosomes. Semin Cell Dev Biol. 2015;40:41–51. 25721812 10.1016/j.semcdb.2015.02.010 PMC4747631

[58] Théry C, Ostrowski M, Segura E. Membrane vesicles as conveyors of immune responses. Nat Rev Immunol. 2009;9(8):581–93. 19498381 10.1038/nri2567

[59] Kalluri R, LeBleu VS. The biology, function, and biomedical applications of exosomes. Science. 2020;367(6478):eaau6977. 32029601 10.1126/science.aau6977 PMC7717626

[60] Than UTT, Guanzon D, Leavesley D, Parker T. Association of Extracellular Membrane Vesicles with Cutaneous Wound Healing. Int J Mol Sci. 2017;18(5):956. 28468315 10.3390/ijms18050956 PMC5454869

[61] Yang JS, Gad H, Lee SY, Mironov A, Zhang L, Beznoussenko GV, et al. A role for phosphatidic acid in COPI vesicle fission yields insights into Golgi maintenance. Nat Cell Biol. 2008;10(10):1146–53. 18776900 10.1038/ncb1774 PMC2756218

[62] Hayashida K, Bartlett AH, Chen Y, Park PW. Molecular and Cellular Mechanisms of Ectodomain Shedding. Anat Rec Hoboken NJ 2007. 2010;293(6):925–37. 10.1002/ar.20757 PMC4621804 20503387

[63] Akers JC, Gonda D, Kim R, Carter BS, Chen CC. Biogenesis of extracellular vesicles (EV): exosomes, microvesicles, retrovirus-like vesicles, and apoptotic bodies. J Neurooncol. 2013;113(1):1–11. 23456661 10.1007/s11060-013-1084-8 PMC5533094

[64] D’Souza-Schorey C, Clancy JW. Tumor-derived microvesicles: shedding light on novel microenvironment modulators and prospective cancer biomarkers. Genes Dev. 2012;26(12):1287–99. 22713869 10.1101/gad.192351.112 PMC3387656

[65] Piccin A, Murphy WG, Smith OP. Circulating microparticles: pathophysiology and clinical implications. Blood Rev. 2007;21(3):157–71. 17118501 10.1016/j.blre.2006.09.001

[66] Connor J, Pak CH, Zwaal RF, Schroit AJ. Bidirectional transbilayer movement of phospholipid analogs in human red blood cells. Evidence for an ATP-dependent and protein-mediated process. J Biol Chem. 1992;267(27):19412–7. 1527061

[67] Leventis PA, Grinstein S. The distribution and function of phosphatidylserine in cellular membranes. Annu Rev Biophys. 2010;39:407–27. 20192774 10.1146/annurev.biophys.093008.131234

[68] Bevers EM, Comfurius P, Dekkers DW, Zwaal RF. Lipid translocation across the plasma membrane of mammalian cells. Biochim Biophys Acta. 1999;1439(3):317–30. 10446420 10.1016/s1388-1981(99)00110-9

[69] Hugel B, Martínez MC, Kunzelmann C, Freyssinet JM. Membrane microparticles: two sides of the coin. Physiol Bethesda Md. 2005;20:22–7. 10.1152/physiol.00029.2004 15653836

[70] Muralidharan-Chari V, Clancy J, Plou C, Romao M, Chavrier P, Raposo G, et al. ARF6-regulated shedding of tumor-cell derived plasma membrane microvesicles. Curr Biol CB. 2009;19(22):1875–85. 19896381 10.1016/j.cub.2009.09.059 PMC3150487

[71] Wilkinson S, Paterson HF, Marshall CJ. Cdc42-MRCK and Rho-ROCK signalling cooperate in myosin phosphorylation and cell invasion. Nat Cell Biol. 2005;7(3):255–61. 15723050 10.1038/ncb1230

[72] Schlienger S, Campbell S, Claing A. ARF1 regulates the Rho/MLC pathway to control EGF-dependent breast cancer cell invasion. Mol Biol Cell. 2014;25(1):17–29. 24196838 10.1091/mbc.E13-06-0335 PMC3873888

[73] Nabhan JF, Hu R, Oh RS, Cohen SN, Lu Q. Formation and release of arrestin domain-containing protein 1-mediated microvesicles (ARMMs) at plasma membrane by recruitment of TSG101 protein. Proc Natl Acad Sci U S A. 2012;109(11):4146–51. 22315426 10.1073/pnas.1200448109 PMC3306724

[74] Wang T, Gilkes DM, Takano N, Xiang L, Luo W, Bishop CJ, et al. Hypoxia-inducible factors and RAB22A mediate formation of microvesicles that stimulate breast cancer invasion and metastasis. Proc Natl Acad Sci U S A. 2014;111(31):E3234–42. 24938788 10.1073/pnas.1410041111 PMC4128139

[75] Rilla K, Pasonen-Seppänen S, Deen AJ, Koistinen VVT, Wojciechowski S, Oikari S, et al. Hyaluronan production enhances shedding of plasma membrane-derived microvesicles. Exp Cell Res. 2013;319(13):2006–18. 23732660 10.1016/j.yexcr.2013.05.021

[76] Sedgwick AE, Clancy JW, Olivia Balmert M, D’Souza-Schorey C. Extracellular microvesicles and invadopodia mediate non-overlapping modes of tumor cell invasion. Sci Rep. 2015;13(5):14748. 10.1038/srep14748 PMC4602187 26458510

[77] McMahon HT, Boucrot E. Molecular mechanism and physiological functions of clathrin-mediated endocytosis. Nat Rev Mol Cell Biol. 2011;12(8):517–33. 21779028 10.1038/nrm3151

[78] Mayor S, Pagano RE. Pathways of clathrin-independent endocytosis. Nat Rev Mol Cell Biol. 2007;8(8):603–12. 17609668 10.1038/nrm2216 PMC7617177

[79] Lau NCH, Yam JWP. From Exosome Biogenesis to Absorption: Key Takeaways for Cancer Research. Cancers. 2023;15(7):1992. 37046653 10.3390/cancers15071992 PMC10093369

[80] Gruenberg J. Life in the lumen: The multivesicular endosome. Traffic Cph Den. 2020;21(1):76–93. 10.1111/tra.12715 PMC7004041 31854087

[81] Peng X, Yang L, Ma Y, Li Y, Li H. Focus on the morphogenesis, fate and the role in tumor progression of multivesicular bodies. Cell Commun Signal CCS. 2020;18(1):122. 32771015 10.1186/s12964-020-00619-5 PMC7414566

[82] Bobrie A, Colombo M, Raposo G, Théry C. Exosome secretion: molecular mechanisms and roles in immune responses. Traffic Cph Den. 2011;12(12):1659–68. 10.1111/j.1600-0854.2011.01225.x 21645191

[83] Zhou L, Shen M, Fan X, Liu Y, Yang L. Pathogenic and Potential Therapeutic Roles of Exosomes Derived From Immune Cells in Liver Diseases. Front Immunol. 2022;4(13):810300. 10.3389/fimmu.2022.810300 PMC8854144 35185900

[84] Gurunathan S, Kang MH, Kim JH. A Comprehensive Review on Factors Influences Biogenesis, Functions, Therapeutic and Clinical Implications of Exosomes. Int J Nanomedicine. 2021;17(16):1281–312. 10.2147/IJN.S291956 PMC7898217 33628021

[85] Oeste CL, Pinar M, Schink KO, Martínez-Turrión J, Stenmark H, Peñalva MA, et al. An Isoprenylation and Palmitoylation Motif Promotes Intraluminal Vesicle Delivery of Proteins in Cells from Distant Species. PLoS ONE. 2014;9(9):e107190. 25207810 10.1371/journal.pone.0107190 PMC4160200

[86] Schmidt O, Teis D. The ESCRT machinery. Curr Biol. 2012;22(4):R116–20. 22361144 10.1016/j.cub.2012.01.028 PMC3314914

[87] Larios J, Mercier V, Roux A, Gruenberg J. ALIX- and ESCRT-III–dependent sorting of tetraspanins to exosomes. J Cell Biol. 2020;219(3):e201904113. 32049272 10.1083/jcb.201904113 PMC7054990

[88] Williams RL, Urbé S. The emerging shape of the ESCRT machinery. Nat Rev Mol Cell Biol. 2007;8(5):355–68. 17450176 10.1038/nrm2162

[89] Groot M, Lee H. Sorting Mechanisms for MicroRNAs into Extracellular Vesicles and Their Associated Diseases. Cells. 2020;9(4):1044. 32331346 10.3390/cells9041044 PMC7226101

[90] Xu D, Di K, Fan B, Wu J, Gu X, Sun Y, et al. MicroRNAs in extracellular vesicles: Sorting mechanisms, diagnostic value, isolation, and detection technology. Front Bioeng Biotechnol. 2022;17(10):948959. 10.3389/fbioe.2022.948959 PMC9618890 36324901

[91] Moreno-Gonzalo O, Fernandez-Delgado I, Sanchez-Madrid F. Post-translational add-ons mark the path in exosomal protein sorting. Cell Mol Life Sci CMLS. 2018;75(1):1–19. 29080091 10.1007/s00018-017-2690-y PMC11105655

[92] Yokoi A, Villar-Prados A, Oliphint PA, Zhang J, Song X, De Hoff P, et al. Mechanisms of nuclear content loading to exosomes. Sci Adv. 2019;5(11):eaax8849. 31799396 10.1126/sciadv.aax8849 PMC6867874

[93] Han QF, Li WJ, Hu KS, Gao J, Zhai WL, Yang JH, et al. Exosome biogenesis: machinery, regulation, and therapeutic implications in cancer. Mol Cancer. 2022;21(1):207. 36320056 10.1186/s12943-022-01671-0 PMC9623991

[94] Kou X, Xu X, Chen C, Sanmillan ML, Cai T, Zhou Y, et al. The Fas/Fap-1/Cav-1 complex regulates IL-1RA secretion in mesenchymal stem cells to accelerate wound healing. Sci Transl Med. 2018;10(432):eaai8524. 29540618 10.1126/scitranslmed.aai8524 PMC6310133

[95] Yang L, Peng X, Li Y, Zhang X, Ma Y, Wu C, et al. Long non-coding RNA HOTAIR promotes exosome secretion by regulating RAB35 and SNAP23 in hepatocellular carcinoma. Mol Cancer. 2019;3(18):78. 10.1186/s12943-019-0990-6 PMC6446409 30943982

[96] Gurung S, Perocheau D, Touramanidou L, Baruteau J. The exosome journey: from biogenesis to uptake and intracellular signalling. Cell Commun Signal CCS. 2021;23(19):47. 10.1186/s12964-021-00730-1 PMC8063428 33892745

[97] Higgins CB, Adams JA, Ward MH, Greenberg ZJ, Milewska M, Sun J, et al. The tetraspanin transmembrane protein CD53 mediates dyslipidemia and integrates inflammatory and metabolic signaling in hepatocytes. J Biol Chem. 2022;299(2):102835. 36581203 10.1016/j.jbc.2022.102835 PMC9900517

[98] Colombo M, Moita C, van Niel G, Kowal J, Vigneron J, Benaroch P, et al. Analysis of ESCRT functions in exosome biogenesis, composition and secretion highlights the heterogeneity of extracellular vesicles. J Cell Sci. 2013;126(24):5553–65. 24105262 10.1242/jcs.128868

[99] Wu SH, Chang MH, Chen YH, Wu HL, Chua HH, Chien CS, et al. The ESCRT-III molecules regulate the apical targeting of bile salt export pump. J Biomed Sci. 2021;28(1):19. 33750401 10.1186/s12929-020-00706-2 PMC7941988

[100] Hurwitz SN, Conlon MM, Rider MA, Brownstein NC, Meckes DG. Nanoparticle analysis sheds budding insights into genetic drivers of extracellular vesicle biogenesis. J Extracell Vesicles. 2016;5:10.3402/jev.v5.31295. 10.3402/jev.v5.31295 PMC4947197 27421995

[101] Peng Y, Zeng Q, Wan L, Ma E, Li H, Yang X, et al. GP73 is a TBC-domain Rab GTPase-activating protein contributing to the pathogenesis of non-alcoholic fatty liver disease without obesity. Nat Commun. 2021;12(1):7004. 34853313 10.1038/s41467-021-27309-1 PMC8636488

[102] Liu C, Liu D, Wang S, Gan L, Yang X, Ma C. Identification of the SNARE complex that mediates the fusion of multivesicular bodies with the plasma membrane in exosome secretion. J Extracell Vesicles. 2023;12(9):12356. 37700095 10.1002/jev2.12356 PMC10497535

[103] Li L, Wang K, Jia R, Xie J, Ma L, Hao Z, et al. Ferroportin-dependent ferroptosis induced by ellagic acid retards liver fibrosis by impairing the SNARE complexes formation. Redox Biol. 2022;56:102435. 36029649 10.1016/j.redox.2022.102435 PMC9425030

[104] Razi M, Futter CE. Distinct Roles for Tsg101 and Hrs in Multivesicular Body Formation and Inward Vesiculation. Mol Biol Cell. 2006;17(8):3469–83. 16707569 10.1091/mbc.E05-11-1054 PMC1525239

[105] Liu Z, Tian Z, Cao K, Zhang B, Wen Q, Zhou X, et al. TSG101 promotes the proliferation, migration and invasion of hepatocellular carcinoma cells by regulating the PEG10. J Cell Mol Med. 2019;23(1):70–82. 30450735 10.1111/jcmm.13878 PMC6307771

[106] Coulter ME, Dorobantu CM, Lodewijk GA, Delalande F, Cianferani S, Ganesh VS, et al. The ESCRT-III Protein CHMP1A Mediates Secretion of Sonic Hedgehog on a Distinctive Subtype of Extracellular Vesicles. Cell Rep. 2018;24(4):973–986.e8. 30044992 10.1016/j.celrep.2018.06.100 PMC6178983

[107] Guo Y, Shang A, Wang S, Wang M. Multidimensional Analysis of CHMP Family Members in Hepatocellular Carcinoma. Int J Gen Med. 2022;10(15):2877–94. 10.2147/IJGM.S350228 PMC8923641 35300135

[108] Wang X, Han S, Liang J, Xu C, Cao R, Liu S, et al. Essential role of Alix in regulating cardiomyocyte exosome biogenesis under physiological and stress conditions. J Mol Cell Cardiol. 2024;1(190):35–47. 10.1016/j.yjmcc.2024.04.001 38593639

[109] Bardens A, Döring T, Stieler J, Prange R. Alix regulates egress of hepatitis B virus naked capsid particles in an ESCRT-independent manner. Cell Microbiol. 2011;13(4):602–19. 21129143 10.1111/j.1462-5822.2010.01557.x PMC7162389

[110] Vallejo MC, Nakayasu ES, Longo LVG, Ganiko L, Lopes FG, Matsuo AL, et al. Lipidomic Analysis of Extracellular Vesicles from the Pathogenic Phase of Paracoccidioides brasiliensis. PLoS ONE. 2012;7(6):e39463. 22745761 10.1371/journal.pone.0039463 PMC3382159

[111] Shama S, Jang H, Wang X, Zhang Y, Shahin NN, Motawi TK, et al. Phosphatidylethanolamines Are Associated with Nonalcoholic Fatty Liver Disease (NAFLD) in Obese Adults and Induce Liver Cell Metabolic Perturbations and Hepatic Stellate Cell Activation. Int J Mol Sci. 2023;24(2):1034. 36674549 10.3390/ijms24021034 PMC9861886

[112] Geier A, Tiniakos D, Denk H, Trauner M. From the origin of NASH to the future of metabolic fatty liver disease. Gut. 2021;70:1570–9. 33632710 10.1136/gutjnl-2020-323202 PMC8292567

[113] Jensen T, Abdelmalek MF, Sullivan S, Nadeau KJ, Green M, Roncal C, et al. Fructose and Sugar: A Major Mediator of Nonalcoholic Fatty Liver Disease. J Hepatol. 2018;68(5):1063–75. 29408694 10.1016/j.jhep.2018.01.019 PMC5893377

[114] Ayonrinde O. Historical narrative from fatty liver in the nineteenth century to contemporary NAFLD – Reconciling the present with the past. JHEP Rep. 2021;3:null. 10.1016/j.jhepr.2021.100261 PMC8135048 34036255

[115] Ludwig J, Viggiano TR, McGill DB, Oh BJ. Nonalcoholic steatohepatitis: Mayo Clinic experiences with a hitherto unnamed disease. Mayo Clin Proc. 1980;55(7):434–8. 7382552

[116] Schaffner F, Thaler H. Nonalcoholic fatty liver disease. Prog Liver Dis. 1986;8:283–98. 3086934

[117] Lee RG. Nonalcoholic steatohepatitis: a study of 49 patients. Hum Pathol. 1989;20(6):594–8. 2656500 10.1016/0046-8177(89)90249-9

[118] Rinella ME, Sookoian S. From NAFLD to MASLD: updated naming and diagnosis criteria for fatty liver disease. J Lipid Res. 2024;65(1):100485. 38103785 10.1016/j.jlr.2023.100485 PMC10824973

[119] Rinella ME, Lazarus JV, Ratziu V, Francque SM, Sanyal AJ, Kanwal F, et al. A multisociety Delphi consensus statement on new fatty liver disease nomenclature. Ann Hepatol. 2024;29(1):101133. 37364816 10.1016/j.aohep.2023.101133

[120] Brunt E, Janney C, Bisceglie A, Neuschwander-Tetri B, Bacon B. Nonalcoholic steatohepatitis: a proposal for grading and staging the histological lesions. Am J Gastroenterol. 1999;94:2467–74. 10484010 10.1111/j.1572-0241.1999.01377.x

[121] Kleiner DE, Brunt EM, Van Natta M, Behling C, Contos MJ, Cummings OW, et al. Design and validation of a histological scoring system for nonalcoholic fatty liver disease. Hepatol Baltim Md. 2005;41(6):1313–21. 10.1002/hep.20701 15915461

[122] Caon E, Martins M, Hodgetts H, Blanken L, Vilia MG, Levi A, et al. Exploring the impact of the PNPLA3 I148M variant on primary human hepatic stellate cells using 3D extracellular matrix models. J Hepatol. 2024;80(6):941–56. 38365182 10.1016/j.jhep.2024.01.032

[123] Romeo S, Kozlitina J, Xing C, Pertsemlidis A, Cox D, Pennacchio LA, et al. Genetic variation in PNPLA3 confers susceptibility to nonalcoholic fatty liver disease. Nat Genet. 2008;40(12):1461–5. 18820647 10.1038/ng.257 PMC2597056

[124] Petta S, Miele L, Bugianesi E, Cammà C, Rosso C, Boccia S, et al. Glucokinase Regulatory Protein Gene Polymorphism Affects Liver Fibrosis in Non-Alcoholic Fatty Liver Disease. PLoS ONE. 2014;9(2):e87523. 24498332 10.1371/journal.pone.0087523 PMC3911959

[125] Chen LZ, Xia HHX, Xin YN, Lin ZH, Xuan SY. TM6SF2 E167K Variant, a Novel Genetic Susceptibility Variant, Contributing to Nonalcoholic Fatty Liver Disease. J Clin Transl Hepatol. 2015;3(4):265–70. 26807382 10.14218/JCTH.2015.00023 PMC4721894

[126] Luo F, Oldoni F, Das A. TM6SF2: A Novel Genetic Player in Nonalcoholic Fatty Liver and Cardiovascular Disease. Hepatol Commun. 2021;6(3):448–60. 34532996 10.1002/hep4.1822 PMC8870032

[127] Shen J, Chan HLY, Wong GLH, Choi PCL, Chan AWH, Chan HY, et al. Non-invasive diagnosis of non-alcoholic steatohepatitis by combined serum biomarkers. J Hepatol. 2012;56(6):1363–70. 22314419 10.1016/j.jhep.2011.12.025

[128] Castera L. Non-invasive tests for liver fibrosis in NAFLD: Creating pathways between primary healthcare and liver clinics. Liver Int Off J Int Assoc Study Liver. 2020;40(Suppl 1):77–81. 10.1111/liv.14347 32077617

[129] El-Kader SMA, Ashmawy EMSED. Non-alcoholic fatty liver disease: The diagnosis and management. World J Hepatol. 2015;7(6):846–58. 10.4254/wjh.v7.i6.846 PMC4411527 25937862

[130] Huh Y, Cho YJ, Nam GE. Recent Epidemiology and Risk Factors of Nonalcoholic Fatty Liver Disease. J Obes Metab Syndr. 2022;31(1):17–27. 35332111 10.7570/jomes22021 PMC8987457

[131] Bass NM, Brunt EM, Clark JM, Diehl AM, Hoofnagle JH, Kleiner DE, et al. Clinical, laboratory and histological associations in adults with nonalcoholic fatty liver disease. Hepatol Baltim Md. 2010;52(3):913–24. 10.1002/hep.23784 PMC3070295 20648476

[132] Uslusoy HS, Nak SG, Gülten M, Biyikli Z. Non-alcoholic steatohepatitis with normal aminotransferase values. World J Gastroenterol. 2009;15(15):1863–8. 19370784 10.3748/wjg.15.1863 PMC2670414

[133] Khella MS, Hamdy NM, Amin AI, El-Mesallamy HO. The (FTO) gene polymorphism is associated with metabolic syndrome risk in Egyptian females: a case- control study. BMC Med Genet. 2017;16(18):101. 10.1186/s12881-017-0461-0 PMC5603034 28915859

[134] Fitzpatrick E, Mitry RR, Quaglia A, Hussain MJ, DeBruyne R, Dhawan A. Serum levels of CK18 M30 and leptin are useful predictors of steatohepatitis and fibrosis in paediatric NAFLD. J Pediatr Gastroenterol Nutr. 2010;51(4):500–6. 20808246 10.1097/MPG.0b013e3181e376be

[135] Papatheodoridis GV, Hadziyannis E, Tsochatzis E, Georgiou A, Kafiri G, Tiniakos DG, et al. Serum apoptotic caspase activity in chronic hepatitis C and nonalcoholic Fatty liver disease. J Clin Gastroenterol. 2010;44(4):e87–95. 19881359 10.1097/MCG.0b013e3181c0945a

[136] Lebensztejn DM, Wierzbicka A, Socha P, Pronicki M, Skiba E, Werpachowska I, et al. Cytokeratin-18 and hyaluronic acid levels predict liver fibrosis in children with non-alcoholic fatty liver disease. Acta Biochim Pol. 2011;58(4):563–6. 22140659

[137] Tamimi TIAR, Elgouhari HM, Alkhouri N, Yerian LM, Berk MP, Lopez R, et al. An Apoptosis Panel for Nonalcoholic Steatohepatitis Diagnosis. J Hepatol. 2011;54(6):1224–9. 21145805 10.1016/j.jhep.2010.08.023 PMC3098936

[138] Joka D, Wahl K, Moeller S, Schlue J, Vaske B, Bahr MJ, et al. Prospective biopsy-controlled evaluation of cell death biomarkers for prediction of liver fibrosis and nonalcoholic steatohepatitis. Hepatology. 2012;55(2):455–64. 21993925 10.1002/hep.24734

[139] Shen J, Chan HLY, Wong GLH, Chan AWH, Choi PCL, Chan HY, et al. Assessment of non-alcoholic fatty liver disease using serum total cell death and apoptosis markers. Aliment Pharmacol Ther. 2012;36(11–12):1057–66. 23066946 10.1111/apt.12091

[140] Feldstein AE, Alkhouri N, De Vito R, Alisi A, Lopez R, Nobili V. Serum cytokeratin-18 fragment levels are useful biomarkers for nonalcoholic steatohepatitis in children. Am J Gastroenterol. 2013;108(9):1526–31. 23752877 10.1038/ajg.2013.168

[141] Kim YS, Jung ES, Hur W, Bae SH, Choi JY, Song MJ, et al. Noninvasive predictors of nonalcoholic steatohepatitis in Korean patients with histologically proven nonalcoholic fatty liver disease. Clin Mol Hepatol. 2013;19(2):120–30. 23837136 10.3350/cmh.2013.19.2.120 PMC3701844

[142] Cusi K, Chang Z, Harrison S, Lomonaco R, Bril F, Orsak B, et al. Limited value of plasma cytokeratin-18 as a biomarker for NASH and fibrosis in patients with non-alcoholic fatty liver disease. J Hepatol. 2014;60(1):167–74. 23973932 10.1016/j.jhep.2013.07.042

[143] Grigorescu M, Crisan D, Radu C, Grigorescu MD, Sparchez Z, Serban A. A novel pathophysiological-based panel of biomarkers for the diagnosis of nonalcoholic steatohepatitis. J Physiol Pharmacol Off J Pol Physiol Soc. 2012;63(4):347–53. 23070083

[144] Pirvulescu I, Gheorghe L, Csiki I, Becheanu G, Dumbravă M, Fica S, et al. Noninvasive clinical model for the diagnosis of nonalcoholic steatohepatitis in overweight and morbidly obese patients undergoing bariatric surgery. Chir Buchar Rom 1990. 2012;107(6):772–9. 23294957

[145] Koehler E, Swain J, Sanderson S, Krishnan A, Watt K, Charlton M. Growth hormone, dehydroepiandrosterone and adiponectin levels in non-alcoholic steatohepatitis: an endocrine signature for advanced fibrosis in obese patients. Liver Int Off J Int Assoc Study Liver. 2012;32(2):279–86. 10.1111/j.1478-3231.2011.02637.x 22098614

[146] Suzuki A, Angulo P, Lymp J, Li D, Satomura S, Lindor K. Hyaluronic acid, an accurate serum marker for severe hepatic fibrosis in patients with non-alcoholic fatty liver disease. Liver Int Off J Int Assoc Study Liver. 2005;25(4):779–86. 10.1111/j.1478-3231.2005.01064.x 15998429

[147] Elhoseeny MM, Abdulaziz BA, Mohamed MA, Elsharaby RM, Rashad GM, Othman AAA. Fetuin-A: a relevant novel serum biomarker for non-invasive diagnosis of metabolic dysfunction-associated steatotic liver disease (MASLD): a retrospective case-control study. BMC Gastroenterol. 2024;24(1):226. 39026172 10.1186/s12876-024-03310-y PMC11264617

[148] Swain M, Nath P, Parida PK, Narayan J, Padhi PK, Pati GK, et al. Biochemical Profile of Nonalcoholic Fatty Liver Disease Patients in Eastern India with Histopathological Correlation. Indian J Clin Biochem. 2017;32(3):306–14. 28811690 10.1007/s12291-016-0612-7 PMC5539007

[149] Bedogni G, Bellentani S, Miglioli L, Masutti F, Passalacqua M, Castiglione A, et al. The Fatty Liver Index: a simple and accurate predictor of hepatic steatosis in the general population. BMC Gastroenterol. 2006;2(6):33. 10.1186/1471-230X-6-33 PMC1636651 17081293

[150] Di Mauro S, Scamporrino A, Filippello A, Di Pino A, Scicali R, Malaguarnera R, et al. Clinical and Molecular Biomarkers for Diagnosis and Staging of NAFLD. Int J Mol Sci. 2021;22(21):11905. 34769333 10.3390/ijms222111905 PMC8585051

[151] Simental-Mendía LE, Rodríguez-Morán M, Guerrero-Romero F. The Product of Fasting Glucose and Triglycerides As Surrogate for Identifying Insulin Resistance in Apparently Healthy Subjects. Metab Syndr Relat Disord. 2008;6(4):299–304. 19067533 10.1089/met.2008.0034

[152] Zhang S, Du T, Zhang J, Lu H, Lin X, Xie J, et al. The triglyceride and glucose index (TyG) is an effective biomarker to identify nonalcoholic fatty liver disease. Lipids Health Dis. 2017;19(16):15. 10.1186/s12944-017-0409-6 PMC5248473 28103934

[153] Du T, Yuan G, Zhang M, Zhou X, Sun X, Yu X. Clinical usefulness of lipid ratios, visceral adiposity indicators, and the triglycerides and glucose index as risk markers of insulin resistance. Cardiovasc Diabetol. 2014;20(13):146. 10.1186/s12933-014-0146-3 PMC4209231 25326814

[154] Lee JH, Kim D, Kim HJ, Lee CH, Yang JI, Kim W, et al. Hepatic steatosis index: a simple screening tool reflecting nonalcoholic fatty liver disease. Dig Liver Dis Off J Ital Soc Gastroenterol Ital Assoc Study Liver. 2010;42(7):503–8. 10.1016/j.dld.2009.08.002 19766548

[155] Harrison SA, Oliver D, Arnold HL, Gogia S, Neuschwander-Tetri BA. Development and validation of a simple NAFLD clinical scoring system for identifying patients without advanced disease. Gut. 2008;57(10):1441–7. 18390575 10.1136/gut.2007.146019

[156] Poynard T, Ratziu V, Naveau S, Thabut D, Charlotte F, Messous D, et al. The diagnostic value of biomarkers (SteatoTest) for the prediction of liver steatosis. Comp Hepatol. 2005;4:10. 16375767 10.1186/1476-5926-4-10 PMC1327680

[157] Nobili V, Parkes J, Bottazzo G, Marcellini M, Cross R, Newman D, et al. Performance of ELF serum markers in predicting fibrosis stage in pediatric non-alcoholic fatty liver disease. Gastroenterology. 2009;136(1):160–7. 18992746 10.1053/j.gastro.2008.09.013

[158] Boursier J, de Ledinghen V, Leroy V, Anty R, Francque S, Salmon D, et al. A stepwise algorithm using an at-a-glance first-line test for the non-invasive diagnosis of advanced liver fibrosis and cirrhosis. J Hepatol. 2017;66(6):1158–65. 28088581 10.1016/j.jhep.2017.01.003

[159] Loong TCW, Wei JL, Leung JCF, Wong GLH, Shu SST, Chim AML, et al. Application of the combined FibroMeter vibration-controlled transient elastography algorithm in Chinese patients with non-alcoholic fatty liver disease. J Gastroenterol Hepatol. 2017;32(7):1363–9. 27936280 10.1111/jgh.13671

[160] Poynard T, Imbert-Bismut F, Munteanu M, Ratziu V. FibroTest-FibroSURE: towards a universal biomarker of liver fibrosis? Expert Rev Mol Diagn. 2005;5(1):15–21. 15723588 10.1586/14737159.5.1.15

[161] Bertot LC, Jeffrey GP, de Boer B, Wang Z, Huang Y, Garas G, et al. Comparative Accuracy of Clinical Fibrosis Markers, Hepascore and Fibroscan® to Detect Advanced Fibrosis in Patients with Nonalcoholic Fatty Liver Disease. Dig Dis Sci. 2023;68(6):2757–67. 36947289 10.1007/s10620-023-07896-3 PMC10188580

[162] Raszeja-Wyszomirska J, Szymanik B, Ławniczak M, Kajor M, Chwist A, Milkiewicz P, et al. Validation of the BARD scoring system in Polish patients with nonalcoholic fatty liver disease (NAFLD). BMC Gastroenterol. 2010;28(10):67. 10.1186/1471-230X-10-67 PMC2905324 20584330

[163] Lee TH, Han SH, Yang JD, Kim D, Ahmed M. Prediction of Advanced Fibrosis in Nonalcoholic Fatty Liver Disease: An Enhanced Model of BARD Score. Gut Liver. 2013;7(3):323–8. 23710314 10.5009/gnl.2013.7.3.323 PMC3661965

[164] Hadizadeh F, Faghihimani E, Adibi P. Nonalcoholic fatty liver disease: Diagnostic biomarkers. World J Gastrointest Pathophysiol. 2017;8(2):11–26. 28573064 10.4291/wjgp.v8.i2.11 PMC5437499

[165] Kruger FC, Daniels CR, Kidd M, Swart G, Brundyn K, van Rensburg C, et al. APRI: a simple bedside marker for advanced fibrosis that can avoid liver biopsy in patients with NAFLD/NASH. South Afr Med J Suid-Afr Tydskr Vir Geneeskd. 2011;101(7):477–80. 21920102

[166] Amato MC, Giordano C, Pitrone M, Galluzzo A. Cut-off points of the visceral adiposity index (VAI) identifying a visceral adipose dysfunction associated with cardiometabolic risk in a Caucasian Sicilian population. Lipids Health Dis. 2011;19(10):183. 10.1186/1476-511X-10-183 PMC3224548 22011564

[167] Amato MC, Giordano C, Galia M, Criscimanna A, Vitabile S, Midiri M, et al. Visceral Adiposity Index. Diabetes Care. 2010;33(4):920–2. 20067971 10.2337/dc09-1825 PMC2845052

[168] Poynard T, Ratziu V, Charlotte F, Messous D, Munteanu M, Imbert-Bismut F, et al. Diagnostic value of biochemical markers (NashTest) for the prediction of non alcoholo steato hepatitis in patients with non-alcoholic fatty liver disease. BMC Gastroenterol. 2006;10(6):34. 10.1186/1471-230X-6-34 PMC1657015 17096854

[169] Younossi Z, Diehl A, Ong J. Nonalcoholic fatty liver disease: An agenda for clinical research. Hepatology. 2002;35:null. 10.1053/jhep.2002.32483 11915019

[170] Younossi ZM, Loomba R, Anstee QM, Rinella ME, Bugianesi E, Marchesini G, et al. Diagnostic Modalities for Nonalcoholic Fatty Liver Disease, Nonalcoholic Steatohepatitis, and Associated Fibrosis. Hepatol Baltim Md. 2018;68(1):349–60. 10.1002/hep.29721 PMC6511364 29222917

[171] Younossi ZM, Jarrar M, Nugent C, Randhawa M, Afendy M, Stepanova M, et al. A novel diagnostic biomarker panel for obesity-related nonalcoholic steatohepatitis (NASH). Obes Surg. 2008;18(11):1430–7. 18500507 10.1007/s11695-008-9506-y

[172] Boyle M, Tiniakos D, Schattenberg JM, Ratziu V, Bugianessi E, Petta S, et al. Performance of the PRO-C3 collagen neo-epitope biomarker in non-alcoholic fatty liver disease. JHEP Rep Innov Hepatol. 2019;1(3):188–98. 10.1016/j.jhepr.2019.06.004 PMC7001575 32039369

[173] Hou J, Wang G, Wang F, Cheng J, Ren H, Zhuang H, et al. Guideline of Prevention and Treatment for Chronic Hepatitis B (2015 Update). J Clin Transl Hepatol. 2017;5(4):297–318. 29226097 10.14218/JCTH.2016.00019 PMC5719188

[174] Bruce M, Kolokythas O, Ferraioli G, Filice C, O’Donnell M. Limitations and artifacts in shear-wave elastography of the liver. Biomed Eng Lett. 2017;7(2):81–9. 30603154 10.1007/s13534-017-0028-1 PMC6208474

[175] Sigrist RMS, Liau J, Kaffas AE, Chammas MC, Willmann JK. Ultrasound Elastography: Review of Techniques and Clinical Applications. Theranostics. 2017;7(5):1303–29. 28435467 10.7150/thno.18650 PMC5399595

[176] Xiao G, Zhu S, Xiao X, Yan L, Yang J, Wu G. Comparison of laboratory tests, ultrasound, or magnetic resonance elastography to detect fibrosis in patients with nonalcoholic fatty liver disease: A meta-analysis. Hepatol Baltim Md. 2017;66(5):1486–501. 10.1002/hep.29302 28586172

[177] Afdhal NH. Fibroscan (Transient Elastography) for the Measurement of Liver Fibrosis. Gastroenterol Hepatol. 2012;8(9):605–7. PMC3594956 23483859

[178] Castéra L, Foucher J, Bernard PH, Carvalho F, Allaix D, Merrouche W, et al. Pitfalls of liver stiffness measurement: a 5-year prospective study of 13,369 examinations. Hepatol Baltim Md. 2010;51(3):828–35. 10.1002/hep.23425 20063276

[179] Controlled Attenuation Parameter (CAP): A Novel VCTE^TM^ Guided Ultrasonic Attenuation Measurement for the Evaluation of Hepatic Steatosis: Preliminary Study and Validation in a Cohort of Patients with Chronic Liver Disease from Various Causes. Ultrasound Med Biol. 2010;36(11):1825–35. 10.1016/j.ultrasmedbio.2010.07.005 20870345

[180] Myers RP, Pollett A, Kirsch R, Pomier-Layrargues G, Beaton M, Levstik M, et al. Controlled Attenuation Parameter (CAP): a noninvasive method for the detection of hepatic steatosis based on transient elastography. Liver Int Off J Int Assoc Study Liver. 2012;32(6):902–10. 10.1111/j.1478-3231.2012.02781.x 22435761

[181] Eddowes PJ, Sasso M, Allison M, Tsochatzis E, Anstee QM, Sheridan D, et al. Accuracy of FibroScan Controlled Attenuation Parameter and Liver Stiffness Measurement in Assessing Steatosis and Fibrosis in Patients With Nonalcoholic Fatty Liver Disease. Gastroenterology. 2019;156(6):1717–30. 30689971 10.1053/j.gastro.2019.01.042

[182] Frulio N, Trillaud H, Perez P, Asselineau J, Vandenhende M, Hessamfar M, et al. Acoustic Radiation Force Impulse (ARFI) and Transient Elastography (TE) for evaluation of liver fibrosis in HIV-HCV co-infected patients. BMC Infect Dis. 2014;21(14):405. 10.1186/1471-2334-14-405 PMC4223715 25041708

[183] Cassinotto C, Boursier J, de Lédinghen V, Lebigot J, Lapuyade B, Cales P, et al. Liver stiffness in nonalcoholic fatty liver disease: A comparison of supersonic shear imaging, FibroScan, and ARFI with liver biopsy. Hepatol Baltim Md. 2016;63(6):1817–27. 10.1002/hep.28394 26659452

[184] Osman AM, El Shimy A, Abd El Aziz MM. 2D shear wave elastography (SWE) performance versus vibration-controlled transient elastography (VCTE/fibroscan) in the assessment of liver stiffness in chronic hepatitis. Insights Imaging. 2020;11:38. 10.1186/s13244-020-0839-y PMC7062958 32152802

[185] Singh S, Venkatesh SK, Wang Z, Miller FH, Motosugi U, Low RN, et al. Diagnostic performance of magnetic resonance elastography in staging liver fibrosis: a systematic review and meta-analysis of individual participant data. Clin Gastroenterol Hepatol Off Clin Pract J Am Gastroenterol Assoc. 2015;13(3):440–451.e6. 10.1016/j.cgh.2014.09.046 PMC4333001 25305349

[186] Lin H, Qiu S, Yang Y, Yang C, Shen Z, Chen Y, et al. Three-dimensional magnetic resonance elastography combining proton-density fat fraction precisely identifies metabolic dysfunction-associated steatohepatitis with significant fibrosis. Magn Reson Imaging. 2023;104:1–8. 37553044 10.1016/j.mri.2023.07.017

[187] Jia S, Zhao Y, Liu J, Guo X, Chen M, Zhou S, et al. Magnetic Resonance Imaging-Proton Density Fat Fraction vs. Transient Elastography-Controlled Attenuation Parameter in Diagnosing Non-alcoholic Fatty Liver Disease in Children and Adolescents: A Meta-Analysis of Diagnostic Accuracy. Front Pediatr. 2021;9:784221. 35087774 10.3389/fped.2021.784221 PMC8787332

[188] Ajmera V, Park CC, Caussy C, Singh S, Hernandez C, Bettencourt R, et al. Magnetic Resonance Imaging Proton Density Fat Fraction Associates With Progression of Fibrosis in Patients With Nonalcoholic Fatty Liver Disease. Gastroenterology. 2018;155(2):307–310.e2. 29660324 10.1053/j.gastro.2018.04.014 PMC6090543

[189] Eddowes PJ, McDonald N, Davies N, Semple SIK, Kendall TJ, Hodson J, et al. Utility and cost evaluation of multiparametric magnetic resonance imaging for the assessment of non-alcoholic fatty liver disease. Aliment Pharmacol Ther. 2018;47(5):631–44. 29271504 10.1111/apt.14469

[190] Gasim GI, Elshehri FM, Kheidr M, Alshubaily FK, ElZaki EM, Musa IR. The Use of Computed Tomography in the Diagnosis of Fatty Liver and Abdominal Fat Distribution among a Saudi Population. Open Access Maced J Med Sci. 2017;5(6):762–5. 29104685 10.3889/oamjms.2017.187 PMC5661714

[191] Li Q, Dhyani M, Grajo JR, Sirlin C, Samir AE. Current status of imaging in nonalcoholic fatty liver disease. World J Hepatol. 2018;10(8):530–42. 30190781 10.4254/wjh.v10.i8.530 PMC6120999

[192] Leow WQ, Chan AWH, Mendoza PGL, Lo R, Yap K, Kim H. Non-alcoholic fatty liver disease: the pathologist’s perspective. Clin Mol Hepatol. 2023;29(Suppl):S302–18. 36384146 10.3350/cmh.2022.0329 PMC10029955

[193] Sumida Y, Nakajima A, Itoh Y. Limitations of liver biopsy and non-invasive diagnostic tests for the diagnosis of nonalcoholic fatty liver disease/nonalcoholic steatohepatitis. World J Gastroenterol WJG. 2014;20(2):475–85. 24574716 10.3748/wjg.v20.i2.475 PMC3923022

[194] Takahashi Y, Dungubat E, Kusano H, Fukusato T. Artificial intelligence and deep learning: New tools for histopathological diagnosis of nonalcoholic fatty liver disease/nonalcoholic steatohepatitis. Comput Struct Biotechnol J. 2023;30(21):2495–501. 10.1016/j.csbj.2023.03.048 PMC10113753 37090431

[195] Ma H, Xu C fu, Shen Z, Yu C hui, Li Y ming. Application of Machine Learning Techniques for Clinical Predictive Modeling: A Cross-Sectional Study on Nonalcoholic Fatty Liver Disease in China. BioMed Res Int. 2018;2018:e4304376. 10.1155/2018/4304376 PMC6192080 30402478

[196] Docherty M, Regnier SA, Capkun G, Balp MM, Ye Q, Janssens N, et al. Development of a novel machine learning model to predict presence of nonalcoholic steatohepatitis. J Am Med Inform Assoc. 2021;28(6):1235–41. 33684933 10.1093/jamia/ocab003 PMC8200272

[197] Njei B, Osta E, Njei N, Al-Ajlouni YA, Lim JK. An explainable machine learning model for prediction of high-risk nonalcoholic steatohepatitis. Sci Rep. 2024;13(14):8589. 10.1038/s41598-024-59183-4 PMC11016071 38615137

[198] Nabrdalik K, Kwiendacz H, Irlik K, Hendel M, Drożdż K, Wijata AM, et al. Machine learning identifies metabolic dysfunction associated steatotic liver disease in patients with diabetes mellitus. J Clin Endocrinol Metab. 2024;dgae060. 10.1210/clinem/dgae060 PMC11244212 38330228

[199] Hassoun S, Bruckmann C, Ciardullo S, Perseghin G, Marra F, Curto A, et al. NAIF: A novel artificial intelligence-based tool for accurate diagnosis of stage F3/F4 liver fibrosis in the general adult population, validated with three external datasets. Int J Med Inf. 2024;185:105373. 10.1016/j.ijmedinf.2024.105373 38395017

[200] Harrison SA, Bedossa P, Guy CD, Schattenberg JM, Loomba R, Taub R, et al. A Phase 3, Randomized, Controlled Trial of Resmetirom in NASH with Liver Fibrosis. N Engl J Med. 2024;390(6):497–509. 38324483 10.1056/NEJMoa2309000

[201] Petta S, Targher G, Romeo S, Pajvani UB, Zheng MH, Aghemo A, et al. The first MASH drug therapy on the horizon: Current perspectives of resmetirom. Liver Int Off J Int Assoc Study Liver. 2024;44(7):1526–36. 10.1111/liv.15930 38578141

[202] Kakizaki M, Yamamoto Y, Nakayama S, Kameda K, Nagashima E, Ito M, et al. Human hepatocyte-derived extracellular vesicles attenuate the carbon tetrachloride-induced acute liver injury in mice. Cell Death Dis. 2021;12(11):1–12. 34707093 10.1038/s41419-021-04204-7 PMC8551237

[203] Németh K, Varga Z, Lenzinger D, Visnovitz T, Koncz A, Hegedűs N, et al. Extracellular vesicle release and uptake by the liver under normo- and hyperlipidemia. Cell Mol Life Sci. 2021;78(23):7589–604. 34665280 10.1007/s00018-021-03969-6 PMC8629784

[204] Jiao Y, Xu P, Shi H, Chen D, Shi H. Advances on liver cell-derived exosomes in liver diseases. J Cell Mol Med. 2021;25(1):15–26. 33247543 10.1111/jcmm.16123 PMC7810930

[205] Royo F, Schlangen K, Palomo L, Gonzalez E, Conde-Vancells J, Berisa A, et al. Transcriptome of extracellular vesicles released by hepatocytes. PLoS ONE. 2013;8(7): e68693. 23874726 10.1371/journal.pone.0068693 PMC3708910

[206] Godakumara K, Dissanayake K, Hasan MM, Kodithuwakku SurangaP, Fazeli A. Role of extracellular vesicles in intercellular communication during reproduction. Reprod Domest Anim Zuchthyg. 2022;57(Suppl 5):14–21. 10.1111/rda.14205 PMC9796405 35837748

[207] Liu Y, Zheng Y, Yang Y, Liu K, Wu J, Gao P, et al. Exosomes in liver fibrosis: The role of modulating hepatic stellate cells and immune cells, and prospects for clinical applications. Front Immunol. 2023;20(14):1133297. 10.3389/fimmu.2023.1133297 PMC10067730 37020547

[208] Kazankov K, Jørgensen SMD, Thomsen KL, Møller HJ, Vilstrup H, George J, et al. The role of macrophages in nonalcoholic fatty liver disease and nonalcoholic steatohepatitis. Nat Rev Gastroenterol Hepatol. 2019;16(3):145–59. 30482910 10.1038/s41575-018-0082-x

[209] Hirsova P, Gores GJ. Death Receptor-Mediated Cell Death and Proinflammatory Signaling in Nonalcoholic Steatohepatitis. Cell Mol Gastroenterol Hepatol. 2014;1(1):17–27. 10.1016/j.jcmgh.2014.11.005 PMC4340657 25729762

[210] Guo Q, Furuta K, Lucien F, Gutierrez Sanchez LH, Hirsova P, Krishnan A, et al. Integrin β1-enriched extracellular vesicles mediate monocyte adhesion and promote liver inflammation in murine NASH. J Hepatol. 2019;71(6):1193–205. 31433301 10.1016/j.jhep.2019.07.019 PMC6864271

[211] Matsuda M, Seki E. Hepatic Stellate Cell-Macrophage Crosstalk in Liver Fibrosis and Carcinogenesis. Semin Liver Dis. 2020;40(3):307–20. 32242330 10.1055/s-0040-1708876 PMC7484001

[212] Alkhouri N, Carter-Kent C, Feldstein AE. Apoptosis in nonalcoholic fatty liver disease: diagnostic and therapeutic implications. Expert Rev Gastroenterol Hepatol. 2011;5(2):201–12. 21476915 10.1586/egh.11.6 PMC3119461

[213] Volkmann X, Fischer U, Bahr MJ, Ott M, Lehner F, Macfarlane M, et al. Increased hepatotoxicity of tumor necrosis factor-related apoptosis-inducing ligand in diseased human liver. Hepatol Baltim Md. 2007;46(5):1498–508. 10.1002/hep.21846 17705261

[214] Feldstein AE, Canbay A, Guicciardi ME, Higuchi H, Bronk SF, Gores GJ. Diet associated hepatic steatosis sensitizes to Fas mediated liver injury in mice. J Hepatol. 2003;39(6):978–83. 14642615 10.1016/s0168-8278(03)00460-4

[215] Koliaki C, Szendroedi J, Kaul K, Jelenik T, Nowotny P, Jankowiak F, et al. Adaptation of hepatic mitochondrial function in humans with non-alcoholic fatty liver is lost in steatohepatitis. Cell Metab. 2015;21(5):739–46. 25955209 10.1016/j.cmet.2015.04.004

[216] Povero D, Eguchi A, Li H, Johnson CD, Papouchado BG, Wree A, et al. Circulating extracellular vesicles with specific proteome and liver microRNAs are potential biomarkers for liver injury in experimental fatty liver disease. PLoS ONE. 2014;9(12):e113651. 25470250 10.1371/journal.pone.0113651 PMC4254757

[217] Canbay A, Feldstein AE, Higuchi H, Werneburg N, Grambihler A, Bronk SF, et al. Kupffer cell engulfment of apoptotic bodies stimulates death ligand and cytokine expression. Hepatol Baltim Md. 2003;38(5):1188–98. 10.1053/jhep.2003.50472 14578857

[218] Povero D, Eguchi A, Niesman IR, Andronikou N, de Mollerat du Jeu X, Mulya A, et al. Lipid-induced toxicity stimulates hepatocytes to release angiogenic microparticles that require Vanin-1 for uptake by endothelial cells. Sci Signal. 2013 Oct 8;6(296):ra88. 10.1126/scisignal.2004512 PMC4016801 24106341

[219] Kakazu E, Mauer AS, Yin M, Malhi H. Hepatocytes release ceramide-enriched pro-inflammatory extracellular vesicles in an IRE1α-dependent manner. J Lipid Res. 2016;57(2):233–45. 26621917 10.1194/jlr.M063412 PMC4727419

[220] Hernández A, Geng Y, Sepúlveda R, Solís N, Torres J, Arab JP, et al. Chemical hypoxia induces pro-inflammatory signals in fat-laden hepatocytes and contributes to cellular crosstalk with Kupffer cells through extracellular vesicles. Biochim Biophys Acta BBA - Mol Basis Dis. 2020;1866(6):165753. 10.1016/j.bbadis.2020.165753 32126269

[221] Duan Y, Pan X, Luo J, Xiao X, Li J, Bestman PL, et al. Association of Inflammatory Cytokines With Non-Alcoholic Fatty Liver Disease. Front Immunol. 2022;6(13):880298. 10.3389/fimmu.2022.880298 PMC9122097 35603224

[222] Liu G, Yin XM. The Role of Extracellular Vesicles in Liver Pathogenesis. Am J Pathol. 2022;192(10):1358–67. 35752228 10.1016/j.ajpath.2022.06.007 PMC9552020

[223] Trifylli EM, Kriebardis AG, Koustas E, Papadopoulos N, Deutsch M, Aloizos G, et al. The Emerging Role of Extracellular Vesicles and Autophagy Machinery in NASH—Future Horizons in NASH Management. Int J Mol Sci. 2022;23(20):12185. 36293042 10.3390/ijms232012185 PMC9603426

[224] Yuan J, Zhang J, Luo Q, Peng L. Effects of nonalcoholic fatty liver disease on sarcopenia: evidence from genetic methods. Sci Rep. 2024;14(1):2709. 38302636 10.1038/s41598-024-53112-1 PMC10834579

[225] Chen M, Cao Y, Ji G, Zhang L. Lean nonalcoholic fatty liver disease and sarcopenia. Front Endocrinol. 2023;23(14):1217249. 10.3389/fendo.2023.1217249 PMC10327437 37424859

[226] Qin L, Wu J, Sun X, Huang X, Huang W, Weng C, et al. The regulatory role of metabolic organ-secreted factors in the nonalcoholic fatty liver disease and cardiovascular disease. Front Cardiovasc Med. 2023;10:1119005. 37180779 10.3389/fcvm.2023.1119005 PMC10169694

[227] Zhang X, Zhao Y, Yan W. The role of extracellular vesicles in skeletal muscle wasting. J Cachexia Sarcopenia Muscle. 2023;14(6):2462–72. 37867162 10.1002/jcsm.13364 PMC10751420

[228] Tarantino G, Sinatti G, Citro V, Santini SJ, Balsano C. Sarcopenia, a condition shared by various diseases: can we alleviate or delay the progression? Intern Emerg Med. 2023;18(7):1887–95. 37490203 10.1007/s11739-023-03339-z PMC10543607

[229] Hirsova P, Ibrahim SH, Krishnan A, Verma VK, Bronk SF, Werneburg NW, et al. Lipid-Induced Signaling Causes Release of Inflammatory Extracellular Vesicles From Hepatocytes. Gastroenterology. 2016;150(4):956–67. 26764184 10.1053/j.gastro.2015.12.037 PMC4808464

[230] Liao CY, Song MJ, Gao Y, Mauer AS, Revzin A, Malhi H. Hepatocyte-Derived Lipotoxic Extracellular Vesicle Sphingosine 1-Phosphate Induces Macrophage Chemotaxis. Front Immunol. 2018;9:2980. 30619336 10.3389/fimmu.2018.02980 PMC6305739

[231] Devhare PB, Sasaki R, Shrivastava S, Di Bisceglie AM, Ray R, Ray RB. Exosome-Mediated Intercellular Communication between Hepatitis C Virus-Infected Hepatocytes and Hepatic Stellate Cells. J Virol. 2017;91(6):e02225–e2316. 28077652 10.1128/JVI.02225-16 PMC5331806

[232] Lee YS, Kim SY, Ko E, Lee JH, Yi HS, Yoo YJ, et al. Exosomes derived from palmitic acid-treated hepatocytes induce fibrotic activation of hepatic stellate cells. Sci Rep. 2017;7(1):3710. 28623272 10.1038/s41598-017-03389-2 PMC5473841

[233] Li X, Chen R, Kemper S, Brigstock DR. Dynamic Changes in Function and Proteomic Composition of Extracellular Vesicles from Hepatic Stellate Cells during Cellular Activation. Cells. 2020;9(2):290. 31991791 10.3390/cells9020290 PMC7072607

[234] Manicardi N, Fernández-Iglesias A, Abad-Jordà L, Royo F, Azkargorta M, Ortega-Ribera M, et al. Transcriptomic Profiling of the Liver Sinusoidal Endothelium during Cirrhosis Reveals Stage-Specific Secretory Signature. Cancers. 2021;13(11):2688. 34072510 10.3390/cancers13112688 PMC8198220

[235] Jiang W, Jin Q, Li C, Xun Y. A Plasma Exosomal Metabolic Profiling of Nonalcoholic Fatty Liver Disease Patients Complicated with Impaired Fasting Glucose. Turk J Gastroenterol. 2024;35(2):125. 38454244 10.5152/tjg.2024.22739 PMC10895878

[236] Shaba E, Vantaggiato L, Governini L, Haxhiu A, Sebastiani G, Fignani D, et al. Multi-Omics Integrative Approach of Extracellular Vesicles: A Future Challenging Milestone. Proteomes. 2022;10(2):12. 35645370 10.3390/proteomes10020012 PMC9149947

[237] Clos-Sansalvador M, Monguió-Tortajada M, Roura S, Franquesa M, Borràs FE. Commonly used methods for extracellular vesicles’ enrichment: Implications in downstream analyses and use. Eur J Cell Biol. 2022;101(3):151227. 35460958 10.1016/j.ejcb.2022.151227

[238] Branković M, Dukić M, Gmizić T, Popadić V, Nikolić N, Sekulić A, et al. New Therapeutic Approaches for the Treatment of Patients with Metabolic Dysfunction-Associated Steatotic Liver Disease (MASLD) and Increased Cardiovascular Risk. Diagnostics. 2024;14(2):229. 38275476 10.3390/diagnostics14020229 PMC10814440

[239] Kou M, Huang L, Yang J, Chiang Z, Chen S, Liu J, et al. Mesenchymal stem cell-derived extracellular vesicles for immunomodulation and regeneration: a next generation therapeutic tool? Cell Death Dis. 2022;13(7):580. 35787632 10.1038/s41419-022-05034-x PMC9252569

[240] Jiang X, Liu Z, You H, Tang Z, Ma Y, Nie R, et al. Extracellular vesicles derived from bone marrow mesenchymal stem cells ameliorate chronic liver damage via microRNA-136–5p. Mol Cell Biochem. 2024. 10.1007/s11010-024-04993-3 38652214

[241] Du X, Li H, Han X, Ma W. Mesenchymal stem cells-derived exosomal miR-24-3p ameliorates non-alcohol fatty liver disease by targeting Keap-1. Biochem Biophys Res Commun. 2022;31(637):331–40. 10.1016/j.bbrc.2022.11.012 36423379

[242] Shi Y, Yang X, Wang S, Wu Y, Zheng L, Tang Y, et al. Human umbilical cord mesenchymal stromal cell-derived exosomes protect against MCD-induced NASH in a mouse model. Stem Cell Res Ther. 2022;13(1):517. 36371344 10.1186/s13287-022-03201-7 PMC9652856

[243] Li T, Fu Y, Guo Z, Zhu H, Liao H, Niu X, et al. A new cell-free therapeutic strategy for liver regeneration: Human placental mesenchymal stem cell-derived extracellular vesicles. J Tissue Eng. 2022;13:20417314221132092. 36313857 10.1177/20417314221132093 PMC9597011

[244] Chen Y, Yang F, Shi Y, Sheng J, Wang Y, Zhang L, et al. RNF31 alleviates liver steatosis by promoting p53/BNIP3-related mitophagy in hepatocytes. Free Radic Biol Med. 2024;219:163–79. 38615890 10.1016/j.freeradbiomed.2024.04.214

[245] Jiang X, Wu Y, Zhong H, Zhang X, Sun X, Liu L, et al. Human milk-derived extracellular vesicles alleviate high fat diet-induced non-alcoholic fatty liver disease in mice. Mol Biol Rep. 2023;50(3):2257–68. 36575319 10.1007/s11033-022-08206-2

[246] Nie YF, Shang JM, Liu DQ, Meng WQ, Ren HP, Li CH, et al. Apical papilla stem cell-derived exosomes regulate lipid metabolism and alleviate inflammation in the MCD-induced mouse NASH model. Biochem Pharmacol. 2024;222:116073. 38395263 10.1016/j.bcp.2024.116073

[247] Zhang L, Liu M, Sun Q, Cheng S, Chi Y, Zhang J, et al. Engineering M2 type macrophage-derived exosomes for autoimmune hepatitis immunotherapy via loading siRIPK3. Biomed Pharmacother. 2024;1(171):116161. 10.1016/j.biopha.2024.116161 38244330

[248] Xu AL, Han L, Yan J, Liu D, Wang W. Effects of Mesenchymal Stem Cells-Derived Extracellular Vesicles on Inhibition of Hepatic Fibrosis by Delivering miR-200a. Tissue Eng Regen Med. 2024. 10.1007/s13770-024-00631-7 PMC11087440 38568409

[249] Niknam B, Baghaei K, Mahmoud Hashemi S, Hatami B, Reza Zali M, Amani D. Human Wharton’s jelly mesenchymal stem cells derived-exosomes enriched by miR-124 promote an anti-fibrotic response in an experimental model of liver fibrosis. Int Immunopharmacol. 2023;119:110294. 37167639 10.1016/j.intimp.2023.110294

[250] Mohamed SR, El-Mahroky SM, Abdel Aal SM. Comparative study between the effect of mesenchymal stem cells microvesicles versus ozone on induced liver injury in adult male albino rats (Histological & Immunohistochemical study). Ultrastruct Pathol. 2024;48(1):16–28. 37997442 10.1080/01913123.2023.2278627

[251] Bruno S, Pasquino C, Herrera Sanchez MB, Tapparo M, Figliolini F, Grange C, et al. HLSC-Derived Extracellular Vesicles Attenuate Liver Fibrosis and Inflammation in a Murine Model of Non-alcoholic Steatohepatitis. Mol Ther. 2020;28(2):479–89. 31757759 10.1016/j.ymthe.2019.10.016 PMC7001005

[252] Chen W, Lin F, Feng X, Yao Q, Yu Y, Gao F, et al. MSC-derived exosomes attenuate hepatic fibrosis in primary sclerosing cholangitis through inhibition of Th17 differentiation. Asian J Pharm Sci. 2024;19(1):100889. 38419761 10.1016/j.ajps.2024.100889 PMC10900800

[253] Scavo MP, Lisco G, Depalo N, Rizzi F, Volpe S, Arrè V, et al. Semaglutide Modulates Extracellular Matrix Production of LX-2 Cells via Exosomes and Improves Metabolic Dysfunction-Associated Steatotic Liver Disease (MASLD). Int J Mol Sci. 2024;25(3):1493. 38338770 10.3390/ijms25031493 PMC10855465

[254] Albaladejo-García V, Morán L, Santos-Coquillat A, González MI, Ye H, Vázquez Ogando E, et al. Curcumin encapsulated in milk small extracellular vesicles as a nanotherapeutic alternative in experimental chronic liver disease. Biomed Pharmacother. 2024;1(173):116381. 10.1016/j.biopha.2024.116381 38452655

[255] Liu Y, Wang L. Extracellular vesicles targeting non-parenchymal cells: the therapeutical effect on liver fibrosis. eGastroenterology. 2024;2. 10.1136/egastro-2023-100040 PMC11770438 39944750

[256] Yu F, Liu Z, Feng J, Man Y, Zhang H, Shi J, et al. Hyaluronic acid modified extracellular vesicles targeting hepatic stellate cells to attenuate hepatic fibrosis. Eur J Pharm Sci. 2024;3:106783. 10.1016/j.ejps.2024.106783 38703918

[257] Lin Y, Yan M, Bai Z, Xie Y, Ren L, Wei J, et al. Huc-MSC-derived exosomes modified with the targeting peptide of aHSCs for liver fibrosis therapy. J Nanobiotechnology. 2022;1(20):432. 10.1186/s12951-022-01636-x PMC9526331 36183106

[258] You DG, Oh BH, Nguyen VQ, Lim GT, Um W, Jung JM, et al. Vitamin A-coupled stem cell-derived extracellular vesicles regulate the fibrotic cascade by targeting activated hepatic stellate cells in vivo. J Control Release Off J Control Release Soc. 2021;10(336):285–95. 10.1016/j.jconrel.2021.06.031 34174353

[259] Sato YT, Umezaki K, Sawada S, Mukai S atsu, Sasaki Y, Harada N, et al. Engineering hybrid exosomes by membrane fusion with liposomes. Sci Rep. 2016;6(1):21933. 10.1038/srep21933 PMC4766490 26911358

[260] Mukherjee D, Paul D, Sarker S, Hasan MN, Ghosh R, Prasad SE, et al. Polyethylene Glycol-Mediated Fusion of Extracellular Vesicles with Cationic Liposomes for the Design of Hybrid Delivery Systems. ACS Appl Bio Mater. 2021;4(12):8259–66. 35005950 10.1021/acsabm.1c00804

[261] Piffoux M, Silva AKA, Wilhelm C, Gazeau F, Tareste D. Modification of Extracellular Vesicles by Fusion with Liposomes for the Design of Personalized Biogenic Drug Delivery Systems. ACS Nano. 2018;12(7):6830–42. 29975503 10.1021/acsnano.8b02053

[262] Evers MJW, van de Wakker SI, de Groot EM, de Jong OG, Gitz-François JJJ, Seinen CS, et al. Functional siRNA Delivery by Extracellular Vesicle-Liposome Hybrid Nanoparticles. Adv Healthc Mater. 2022;11(5):2101202. 34382360 10.1002/adhm.202101202 PMC11468224

[263] Roy S, Hochberg FH, Jones PS. Extracellular vesicles: the growth as diagnostics and therapeutics; a survey. J Extracell Vesicles. 2018;7(1):1438720. 29511461 10.1080/20013078.2018.1438720 PMC5827771

[264] Wei B, Huang H, Cao Q, Song X, Zhang Z. Bibliometric and visualized analysis of the applications of exosomes based drug delivery. Biomed Pharmacother Biomedecine Pharmacother. 2024;176:116803. 10.1016/j.biopha.2024.116803 38788602

