# Supplementary material for: Extracellular vesicle-mediated approaches for the diagnosis and therapy of MASLD: current advances and future prospective
Source: Lipids Health Dis. 2025 Jan 7;24:5. doi: 10.1186/s12944-024-02396-3 (PMC11705780; doi:10.1186/s12944-024-02396-3)
Supplement: Supplementary file 1 — Supplementary Material 1. [file 12944_2024_2396_MOESM1_ESM.docx]

**Supplementary Table 1: Noninvasive Biomarkers of MASLD**

| Serial Number | Biomarker | Population | Stage | Technique | Expression Pattern: Downregulation regulation/  Upregulation regulation | Sensitivity (%) | Specificity (%) | Cut Off | References |
| --- | --- | --- | --- | --- | --- | --- | --- | --- | --- |
| 1 | CK18 | Children | Fibrosis | M30 ELISA | Upregulation | 83 | 40 | 200 IU/L | (134) |
| 2 | CK18 | Children | NASH | M30 ELISA | Upregulation | 84 | 88 | 207 IU/L | (134) |
| 3 | CK18 | Adults | NASH | M30 ELISA | Upregulation | 70 | 82 | ≥225 U/L | (135) |
| 4 | CK18 | Adults | NASH | M30 ELISA | Upregulation | 60 | 93 | ≥250 U/L | (135) |
| 5 | CK18 | Adults | NASH | M30 ELISA | Upregulation | 53 | 100 | ≥300 U/L | (135) |
| 6 | CK18 | Children | Fibrosis | M30 ELISA | Upregulation | 79 | 60 | 210 U/L | (136) |
| 7 | CK-18 + sFas | Adults | NASH | M30 ELISA + ELISA | Upregulation | 88 | 89 | −0.5509 (Risk Score Model) | (137) |
| 8 | CK18 | Adults | Fibrosis | M30 ELISA | Upregulation | 64 | 61 | 157.5 U/L | (138) |
| 9 | CK18 | Adults | NASH | M30 ELISA | Upregulation | 75 | 70 | 149.5 U/L | (138) |
| 10 | CK18 | Adults | Fibrosis | M65 ELISA | Upregulation | 71 | 67 | 479.5 U/L | (138) |
| 11 | CK18 | Adults | NASH | M65 ELISA | Upregulation | 100 | 80 | 386.0 U/L | (138) |
| 12 | CK18 | Adults | Fibrosis | M65ED ELISA | Upregulation | 74 | 68 | 353.0 U/L | (138) |
| 13 | CK18 | Adults | NASH | M65ED ELISA | Upregulation | 100 | 80 | 237.0 U/L | (138) |
| 14 | CK18 | Adults | NAFLD | M30 ELISA | Upregulation | 84.2 | 91.9 | 180 U/L | (127) |
| 15 | CK18 | Adults | NAFLD | M30 ELISA | Upregulation | 66.2 | 65.6 | 338 U/L | (127) |
| 16 | CK18 | Adults | NAFLD | M30 ELISA | Upregulation | 84.4 | 90.4 | 180 U/L | (139) |
| 17 | CK18 | Adults | NAFLD | M65 ELISA | Upregulation | 76.9 | 95.9 | 523 U/L | (139) |
| 18 | CK18 | Adults | NAFLD | M65ED ELISA | Upregulation | 93.2 | 79.5 | 105 U/L | (139) |
| 19 | CK18 | Adults | NASH | M30 ELISA | Upregulation | 66.7 | 60.3 | 338 U/L | (139) |
| 20 | CK18 | Adults | NASH | M65 ELISA | Upregulation | 62.3 | 70.5 | 790 U/L | (139) |
| 21 | CK18 | Adults | NASH | M65ED ELISA | Upregulation | 79.7 | 57.7 | 309 U/L | (139) |
| 22 | CK18 | Children | NASH | M30 ELISA | Upregulation | 85 | 86.9 | 233 U/L | (140) |
| 23 | CK18 | Adults | NASH | M30 ELISA | Upregulation | 69 | 64.9 | 235.5 U/L | (141) |
| 24 | CK18 | Adults | NAFLD | M30 ELISA | Upregulation | 63 | 83 | 165 U/L | (142) |
| 25 | CK18 | Adults | NASH | M30 ELISA | Upregulation | 58 | 68 | 212 U/L | (142) |
| 26 | CK18 | Adults | Fibrosis | M30 ELISA | Upregulation | 54 | 85 | 262 U/L | (142) |
| 27 | CK18 | Adults | NASH | M65 ELISA | Upregulation | 79.3 | 66.7 | ≥340 U/L | (143) |
| 28 | CK18 | Adults | NASH | M30 ELISA | Upregulation | 69.2 | 65.2 | 136 U/L | (144) |
| 29 | CK18 | Adults | NASH | M65 ELISA | Upregulation | 69.2 | 71.7 | 389 U/L | (144) |
| 30 | Adiponectin | Adults | NASH | ELISA | Downregulation | 86.2 | 61.9 | ≥6.0 µg/mL | (143) |
| 31 | Adiponectin | Adults | NASH | ELISA | Downregulation | 92.3 | 86.7 | 13.5 µg/mL | (144) |
| 32 | Leptin | Adults | NASH | ELISA | Upregulation | 61.5 | 65.9 | 40 ng/mL | (144) |
| 33 | Adiponectin | Adults | NASH | ELISA | Downregulation | 63 | 94 | 4.080 µg/mL | (145) |
| 34 | Hyaluronic Acid | Adults | Fibrosis | Liquid Phase Binding Assay | Upregulation | 85 | 80 | 46.1 µg/l | (146) |
| 35 | Hyaluronic Acid | Children | Fibrosis | ELISA | Upregulation | 84 | 55 | 19.1 ng/mL | (136) |
| 36 | Fetuin-A | Adults | MASLD | ELISA | Upregulation | 82 | 90 | > 702.5 ng/mL | (147) |

NAFLD, nonalcoholic fatty liver disease; NASH, nonalcoholic steatohepatitis; ELISA, enzyme-linked immunosorbent assay; CK18, Cytokeratin 18; MASLD, metabolic-associated steatohepatitis; IU/L, international units per liter; U/L, units per liter, ng, nanograms, mL, millions; µg, micrograms; L, liters.
