# Supplementary material for: Extracellular vesicle-mediated approaches for the diagnosis and therapy of MASLD: current advances and future prospective
Source: Lipids Health Dis. 2025 Jan 7;24:5. doi: 10.1186/s12944-024-02396-3 (PMC11705780; doi:10.1186/s12944-024-02396-3)
Supplement: Supplementary file 5 — Supplementary Material 5. [file 12944_2024_2396_MOESM5_ESM.pdf]

Extracellular Vesicle Mediated Approaches for D...

By: Swasthika Gurjar

As of: Nov 30, 2024 5:06:36 AM
13,543 words - 139 matches - 98 sources

Similarity Index

10%

Mode: Similarity Report

paper text:

Extracellular Vesicle-Mediated Approaches for Diagnosis and Therapy in MASLD: Current Advances and Future Prospective

Swasthika Gurjar 1*, Ramanarayana Bhat A2*, Raghavendra Upadhya2#, Revathi P Shenoy1# Affiliation: 1Department

of Biochemistry, Kasturba Medical College, Manipal, Manipal Academy of Higher Education, Karnataka – 576104 , Manipal, **India** 58

. 2Manipal Centre for Biotherapeutics Research,

Manipal, Manipal Academy of Higher Education, Karnataka – 576104 , Manipal, **India** . * Equal Contribution # **Corresponding Author** 42

Abstract: Metabolic dysfunction Associated Steatotic Liver Disease (MASLD) is 34

an asymptomatic multifaceted condition often associated with various

risk factors, including fatigue, obesity, **insulin resistance, metabolic syndrome, and** 38

sleep apnea. The escalating burden of MASLD underscores the critical need for early diagnosis and effective therapies. The lack of efficient therapies for MASLD makes early diagnosis crucial. Consequently, non-invasive biomarkers and imaging techniques are essential in analyzing disease risk

and play a pivotal role in the 97

global diagnostic process.

**Extracellular Vesicles have emerged as a** hope **for**	57

early diagnosis and therapy of various liver ailments. Therefore,

**this article** aims **to provide a comprehensive** summary **of the**	15

current diagnostic modalities for MASLD, highlighting their advantages and limitations, while exploring the potential of EVs as a novel diagnostic and therapeutic modality for MASLD. With this aim, the review emphasizes an in-depth understanding of the origin of EVs and pathophysiological alterations of these ectosomes and exosomes in various liver diseases. The review also explores the therapeutic potential of EVs as a key component in the future management of liver disease. The dual role of EVs as biomarkers and therapeutic utility in MASLD essentially highlights their clinical integration to improve MASLD diagnosis and treatment. While EV-based therapies are still in their early stage of development and require substantial research to enhance their therapeutic values before reaching clinical use, the diagnostic application of EVs has been extensively explored. Developing diagnostic devices leveraging EVs will be crucial in advancing MASLD diagnosis. Thus, the literature summarized provides suitable grounds for clinicians and researchers to explore the EVs for devising the diagnosis and treatment strategies for the

**MASLD. Keywords: Metabolic dysfunction Associated Steatotic Liver Disease, Liver**	43

diseases, Extracellular vesicles, Biomarker, Targeted therapy. 1. Introduction: Lifestyle has been known to determine the quality of an individual's life by influencing various factors, including physical and mental well-being.

**Metabolic dysfunction Associated Steatotic Liver Disease (MASLD) is** one such **hepatic**	34

pathology in developed countries, affecting about one-fourth of the population at the global level. In the rapidly growing era of the urban lifestyle, the adoption of a sedentary lifestyle accompanied by unhealthy dietary patterns has significantly contributed to health-related diseases, especially non-communicable diseases. (1,2)

**including obesity (3), diabetes mellitus (4), hypertension (5**), dyslipidemia (**6**	29

), cardiovascular diseases (7) as well as metabolic syndrome (8), and musculoskeletal diseases (9). Furthermore, it has also been shown to affect the quality of sleep (10), life expectancy (11), and productivity (12), social interaction (13). The liver, being the metabolic center of the human body, is strongly affected by the combined effect of an inappropriate diet and a state of physical inactivity. It is reported that overnutrition causes energy imbalance in the metabolic process and accumulation of fatty acids in the liver. Besides, reduced fatty oxidation in the liver as a result of physical inactivity exacerbates liver health (14,15). MASLD is one such outcome of hepatic pathology (16), which was earlier

known as Non-Alcoholic Fatty Liver Disease . MASLD is **a liver** pathology **characterized by** 69

the sequential progression from simple benign to more severe form and mainly consists of simple steatosis or

Metabolic dysfunction Associated Steatotic Liver and Metabolic dysfunction associated Steatohepatitis 34

. MASLD harbors additional

complications such as fibrosis, **cirrhosis, and** end-stage **Hepatocellular Carcinoma (HCC** 90

) (17,18). It is a condition of hepatic steatosis that is primarily associated with

cardiometabolic risk factors such as obesity, insulin resistance, dyslipidemia, and hypertension 3

with the exclusion of other identifiable causes (19). According to a systematic review conducted in the year 2022, the worldwide prevalence of MASLD (NAFLD) is 32.4 % with an increase from 25.5 % between 1990 to 2022. Though there is an increase in its prevalence in women, it is lower than in men (20). In the case of mortality, there is a 1.93-fold higher risk of death in the MASLD population compared to the general population according to a study conducted in 2020 (21). The pathology of this disease during the initial stage manifests as a silent killer with no potential symptoms but later results in severe health complications in the absence of an early diagnosis (22). In such circumstances, histopathological analysis stands as the sole diagnostic method for most hepatic diseases and MASLD follows suit without exception. The conventional diagnostic method for MASLD involves a cell-penetrating method of reaping the tissue sample, these are often associated with the risk of death (23). Even though the biochemical parameters can elucidate some of the metabolic variations associated with hepato-pathophysiology, understating the hepatic ailment by employing these parameters alone would be misleading ending up with misinterpretations. Delving into these noninvasive techniques, there is no single

biomarker that accurately assists in diagnosis and staging whereas imaging techniques lack sensitivity in detecting the disease progression (24,25). Early diagnosis remains the only effective strategy for addressing the disease before it progresses too far. Liquid biopsy is an emerging convenient alternative way of diagnosis and monitoring molecular changes associated with various diseases including cancer (26,27). This minimally invasive technology simplifies the task of sampling and reduces the risk associated with diagnosis. Presently, the technique finds prominent utilization in cancer screening particularly screening of genetic aberrations originating from any combinations of components like Extracellular Vesicles (EVs)(28–30), circulating tumor cells (31), as well as cell-free DNA (32). These circulating components with higher accessibility can be utilized in real-time monitoring of disease progression enabling the selection of appropriate personalized therapy (33). EVs present in circulating blood

can provide valuable information about the physiological status **of the** 52

parent cells (34,35). EVs are tiny nano-sized heterogenous sized entities, delimited by a lipid bilayer. These tiny vesicles irrespective of their size carry a variety of molecular cargos. They are incapable of replicating themselves, compelling these particles to information about the parent cells (36). EVs are primarily engaged in cellular communications. Additionally, they can also influence cell signaling cascades leading to activation of multiple pathways thereby participating in both normal physiological and pathophysiological functions (37). The EVs are a heterogenous class of particles encompassing ectosomes produced

by outward budding whereas **plasma membrane fusion of** 98

parent cell, exosomes originate in

the endosomal network which is **released upon fusion of** the **multivesicular** body to **the plasma membrane; and apoptotic bodies** that **are released as blebs of cells undergoing apoptosis** 26

(36,38,39). A variety of body fluids such as plasma (29,40), serum (41), urine (42,43), cerebrospinal fluid (44), and saliva (45,46) are known to serve as abundant sources of biomarker-containing EVs. Enclosed within them are many molecular cargos

such as proteins, **nucleic acids, and lipids** (47) which **can** attain **signature** 26

confirmation upon reaching certain physiological states or disease stages. Even highly fragile molecules such as RNA can remain intact and protected from degradation by the action of RNases when they are sorted and packed carefully within the EVs (48). Multiple studies have experimentally curated EVs as a source of biomarkers for liver-related diseases. EVs have been extensively explored in the past and continue to be investigated for a variety of liver diseases, including MASLD (49),

Alcoholic Fatty Liver Disease (AFLD) (50), **Drug-Induced Liver Injury (DILI**) (51), **Autoimmune Hepatitis (AIH**) (52), **HCC** (53), **and viral hepatitis** 12

(54). Review Aim:

The aim of this study is to explore and summarize **the** 22

recent advancements in the diagnosis and treatment of MASLD, with a particular focus on the role of EVs in clinical applications. This review expounds on commonly employed diagnostic biomarkers in clinical settings for MASLD diagnosis. Additionally, it highlights recent discoveries of blood-based biomarkers with promising diagnostic potential for MASLD. With EV-centered approaches, this review aims to understand the effective utilization of these cellular vehicles in MASLD diagnosis and therapy, covering the current advances and prospects. In line with this aim, the relevant literature was primarily selected from the PubMed database, focusing on clinically significant data. The publications from 1999 to July 2024, with a particular emphasis on research from the past decade were included. This approach aligns with trends shown in Figure 2, which demonstrate a substantial rise in studies related to MASLD in the last ten years, reflecting the growing public health focus on its diagnosis and treatment. Similarly, research on EVs has expanded rapidly over the same period, especially in the fields of diagnostics and therapy. The overlap in these research timelines provided a strong rationale for concentrating literature selection within this timeframe. 2. Extracellular Vesicles- A Comprehensive Overview: 2.1 Extracellular vesicles: EVs which were previously disregarded as cellular debris (55) are tiny heterogeneous classes of naturally occurring nanoparticles delimited by the plasma membrane. These tiny particles lack the ability of self-replication and are produced by the parental cells to the extracellular spaces to exhibit a plethora of physiological functions (56). The classification of EVs is still debatable however based on the biogenetic pathway, the EVs can be either ectosomes or exosomes (57). The

ectosomes are formed by the outward membrane blebbing **of the** 87

cells whereas

exosomes are formed by inward blebbing of endosomal membrane followed by the 25

formation of multivesicular bodies, which releases exosomes by exocytosis

upon fusion with the plasma membrane 56

(36,58). The biogenetic mechanisms of ectosomes and exosomes are intricate complex cellular mechanisms involving sorting mechanisms that specifically load specific molecular cargo such as RNA, lipids, and proteins into the vesicles making them distinct from parental cells (59). Interestingly, exosome biogenesis is eukaryote -specific as it requires endosomes, while ectosomes are produced by both prokaryotic and eukaryotic cells (60,61). 2.2 Biogenesis of circulating EVs: 2.2.1 Biogenesis of Ectosomes: The ectosomes

were first described as subcellular particles derived **from platelets in normal** serum **and** 70

plasma, and they were often termed “platelet dust”(55). Later ectocytosis was described using stimulated neutrophils. Several studies based on ectocytosis termed them as shedding bodies or shedding particles and oncosomes ascertaining their functions and roles played in cellular communication. They are produced by a biogenetic process which involves the vertical transfer of molecular cargo to the plasma membrane which is subsequently packed in lipid bilayer particles via a distinct pathway. The parent cells utilize a distinct contractile machinery that enables cells to pinch off these vesicles at the cell surface (62). The complexity of this biogenetic pathway is intermediate which is neither as complicated as the biogenesis of exosomes nor as simple as apoptotic bodies which are produced due to indiscriminate plasma membrane blebbing (63). Ectosomes originate due to membrane blebbing which is usually associated with specific changes in

the lipid and protein components at specific sites of the plasma membrane 23

changing its properties such as its rigidity and curvature (64). The formation of ectosomes is achieved due to the dynamic interplay of phospholipid redistribution and contraction of cytoskeletal proteins (65). A diverse range of eukaryotic cells produce ectosomes under normal physiological conditions as well as during disease conditions. Under the disease condition, the highly regulated biogenetic pathway can have abrupt changes leading to aberrant shedding of ectosomes (64). The biogenetic pathway and the factors influencing the biogenesis of ectosomes under normal physiological conditions and in altered physiological conditions are summarised below. 2.2.1.1 Mechanism of Ectosomes Biogenesis

under normal physiological condition: 2.2.1.1.1 Change in lipid composition: The structural properties and shapes of the lipids depend upon their hydrophilic head groups, hydrophobic acyl chain length, and saturation.

The composition of the inner and outer leaflets 11

of the plasma membrane is distinct from each other, wherein the inner leaflet predominantly harbors the amino phospholipids such as Phosphatidyl Serine (PS) and the external leaflet is enriched with sphingomyelin and phosphatidylcholine. In general, vesicle formation is associated with the change in lipid composition assisted by the PS and local recruitment of the lipid-modifying enzymes such as aminophospholipid translocases, flippases, floppases, gelsolin, scramblase, and calpain (66,67). The lipid composition is greatly influenced by these enzymes wherein flippases translocate specifically PS into the inner leaflet and floppases translocate lipids outwards. However, the enzyme scramblase promotes the unspecific bidirectional distribution of lipids across the plasma membrane (67–70). The membrane asymmetry collapses during ectosome biogenesis, with an increase of cytosolic Ca2+ concentration activating floppases and scramblases while simultaneously inhibiting flippases. The biodistribution of PS induces the signals which release the ectosomes. The induction of budding/ vesicle formation signal occurs due to surface exposure of phosphatidylserine wherein translocation of PS occurs

from the inner leaflet **to** the **outer leaflet of the plasma membrane** 25

(71). 2.2.1.1.2 Activation of contractile machinery: The formation of ectosomes is a well- orchestrated cellular event wherein phospholipid redistribution coincides with contractile machinery majorly governed by cytoskeletal proteins. Cytoskeleton contractile machinery relies on a set of enzymes such as

ADP-ribosylation factor 6 (ARF6) and **Myosin light chain kinase (MLCK** 25

).

ADP-ribosylation factor 6 (ARF6) is a small GTPase protein **that** 48

activates Phospholipase D and activated

Phospholipase D recruits extracellular signal-regulated kinase (ERK). ERK recruited at **the plasma membrane** activates MLCK by **phosphorylation** (72). The biogenesis **of** 14

ectosomes is completed through cytoskeletal contractions regulated by enzymes that govern the actin and myosin interaction (65). The phosphorylation of MLCK on Thr- 18/Ser-19 induces the actin – myosin-based cytoskeletal contraction by generating the necessary force required for ectosomes budding/shedding (72). This enhances the activity of Myosin II and the enhanced activity of Myosin II enables it to engage in highly efficient interaction with actin filaments increasing the cellular contraction (73). The study on the regulation of Rho/MLC pathway by ADP-ribosylation factor 1 (ARF1) for controlling breast cancer cell invasion demonstrated that ARF1 also functions like ARF6 and plays a crucial role in cytoskeletal contractile machinery (74). 2.2.1.2 Ectosomes Biogenesis in Disease and Altered Physiological Conditions: The ectosomes biogenesis can be abruptly altered in pathological and altered physiological conditions. Under the altered physiological conditions, the biogenesis can be affected by several factors. Some of the factors affecting the biogenesis of ectosomes and the way of biogenetic mechanism are discussed below. 2.2.1.2.1 ARRDC1-Mediated Ectosomes Biogenesis: The ectosome biogenesis can invariably exploit Tumor Suppressing Gene 101 (

TSG101) protein and Endosomal Sorting Complex Required for Transport (ESCRT) machinery 22

to produce ectosomes. A study on

Arrestin domain-containing protein 1-mediated ectosomes (**ARMMs** 57

) demonstrated that Arrestin Domain Containing 1 (ARRDC1) recruits TSG101 to the surface of the cells to produce ectosomes. The ectosomes produced are distinct from exosomes as they are devoid of late endosomal

markers such as CD63 and **Lysosomal Associated Membrane Protein 1 (LAMP1** 1

) indicating that these vesicles are released by direct plasma membrane budding (75). 2.2.1.2.2 Hypoxia Ectosomes Biogenesis: Investigation on Hypoxia Inducible Factors (HIFs) in breast cancer

invasion and metastasis demonstrated **that the** hypoxia in **breast cancer** 95

cells induces an increase in Ras-related protein Rab-22A (RAB22A) gene expression which co- localizes with increased expression of ectosomes formation. It is also evident from the study that RAB22A had limited influence on ectosomes formation under non-hypoxic conditions. The study suggested selective recruitment of RAB proteins under hypoxia conditions for the shedding of ectosomes (76). Hypoxia can exacerbate liver inflammation and fibrosis through the activation of hypoxia-inducible factors in MASLD. 2.2.1.2.3 Hyaluronan Production and Ectosomes Biogenesis: Hyaluronan synthesis coincides with various physiological events involving rapid tissue remodeling phases such as embryonic development, inflammation, wound healing, and malignant tumor formation. Rilla et al. (2013) revealed that hyaluronan synthesis induces enhanced secretion of ectosomes. It is hypothesized that the ectosomes are shed either from tips of Hyaluronan Synthase (HAS) induced microvilli or through the

budding of the plasma membrane. It is believed **that** 88

cells synthesizing Hyaluronan in high quantity generally harbor microvilli which can be a platform for ectosomes formation. It was observed that HAS activity is also influenced by cholesterol, and cellular cholesterol influences the secretion of microvesicles, thus this study hypothesizes that the microvesicle secretion occurs at the plasma membrane due to confirmational changes caused in lipid rafts due to HAS-induced hyaluronan synthesis (77). 2.2.1.2.4 RhoA mediated ectosomes formation:

Ras-related C3 botulinum toxin substrate 1 (RAC1) and Ras homolog gene family, member A (RhoA) 51
signaling **is**

important for promoting invadopodia or ectosomes in tumor cells. The Rho family proteins Rac 1 and Rho A act against each other and the action of these determines the switching of tumor cell phenotype between ameboid and mesenchymal which are distinct from each other, the former being involved in the shedding of ectosomes and later in utilizing invadopodium. The tumor cell-derived ectosome formation is primarily driven by the Rho-ROCK pathway which involves ARF6 activation in its downstream (78).The RhoA-mediated ectosome formation may actively be involved in HCC in the production of cancer ectosomes. 2.2.2 Biogenesis of Exosomes: Exosomes originate from the endosomes on exocytosis of multivesicular bodies. The biogenesis of exosomes is the most complex and well-coordinated cellular event. The complex cellular event of exosome biogenesis can be enumerated into several key events such as Endocytosis, Early endosome formation, formation of Multivesicular Bodies (MVB), Intraluminal Vesicle (ILV) formation with molecular cargo sorting, Multivesicular body maturation, and exosome release. Early endosome formation is the first step of exosome biogenesis which begins with the endocytosis which can be

clathrin- mediated or **caveolin mediated or clathrin** or **caveolin independent endocytosis** 28

(59). 2.2.2.1 Endocytosis and Early endosome formation: 2.

2.2 .1. **1 Clathrin-mediated endocytosis** 39

: Cellular uptake was first visualized using glutaraldehyde fixation under electron microscopy in 1960, leading to the discovery of vesicles coated with proteinaceous substances. Clathrin was then identified as a major protein of proteinaceous coating around the vesicles being taken up. Clathrin-mediated endocytosis is explained in detail in previous literature. It involves a clathrin-coated vesicle cycle with

five stages including **nucleation, cargo selection, clathrin coat assembly, vesicle scission** , vesicle formation, **and** budding. Briefly, **nucleation** 66

begins with membrane invagination driven by

F- BAR (Fes/CIP4 Homology-Bin/Amphiphysin/Rvs) domain-containing 45

proteins (FCHO proteins), Epidermal Growth Factor Receptor Pathway Substrate 15 (EPS15) and intersectins 47

. The nucleation model then recruits clathrin for budding and Adaptor Protein Complex 2 (AP2) for cargo selection. Clathrin then stabilizes the vesicle, while Dynamin enables scission. The Heat Shock Cognate 70 (HSC70) disassembles the coat allowing clathrin recycling (79). 2.

2.2.1 .2 **Clathrin** -Independent **endocytosis: Clathrin** -independent **endocytosis** 39

poses challenges due to membrane flexibility and restrictions for capturing molecular cargo in small areas. Caveolae-mediated endocytosis is one of the major Clathrin-independent endocytosis machinery. Small pits on the plasma membrane characterized by proteins like caveolin and Cavins called Caveolae can dynamically detach from the membrane to form endocytic carriers(80). In addition to Caveolae-mediated

endocytosis there are several Clathrin- Independent endocytosis **pathways** 94

including

clathrin-independent carriers/GPI-AP-enriched early endosomal compartments (CLIC/GEEC) pathway and the ARF6-associated pathway. The detailed mechanisms **of these pathways are not well** understood; however, **reorganization of the actin cytoskeleton** is **a** common key factor **in** 9

all of these pathways (80,81). 2.2.2.1.3 Early endosome formation: Endocytosis results in the formation of pleomorphic structures known as early endosomes. They

play a central **role in** regulating **the** recycling **and** breakdown **of membrane** elements. Few **components** 48

of early endosomes are recycled while others are transported into trans-Golgi networks. Molecular cargo predetermined for late endosomes or EVs is sorted into Intra Luminal Vesicles (ILVs) which results in the formation of Multi Vesicular Endosomes (MVEs) (82). Multivesicular bodies were initially considered important components of the endosomal lysosomal degradation pathway (83). These multivesicular bodies have multiple fates; they can be sorted towards late endosomes followed by delivery to lysosomes or plasma membrane. The molecular cargo destined for degradation follows the former path whereas the cargo involved in cellular communication through exosomes ends up with the later path. 2.2.2.2 Cargo Sorting, Multivesicular Body Formation, and Its maturation: Even though the exosomes are tiny they

carry a wide **variety of** molecular cargo **including proteins, lipids** , metabolites, **and** 85

various forms of RNA such as

messenger RNA (mRNA), microRNA (miRNA), long non-coding RNA (lncRNA), circular RNA (circRNA), and PIWI-interacting RNA (piRNA 9

) (59,81,84,85). The physiological state of parent cells from which exosomes are produced greatly influences the molecular profile of cargo in them. And interestingly exosomes attain definitive cellular functions based on the molecular cargo carried

by them (86). Hence precise sorting of these cargoes is a crucial aspect of exosome biogenesis and its function. Protein cargo sorting: Ubiquitylation and Farnesylation are the two important post- translational protein modifications that

play a prominent role in the segregation of certain 46

proteins into ILVs (87). The sorting of molecular cargo occurs through

ESCRT-dependent and ESCRT-independent pathways 46

. ESCRT

plays a key role in the formation **of** ILVs **by** 53

incorporating specific protein cargo. The key components of the pathway

include Hepatocyte Growth Factor-Regulated Tyrosine Kinase Substrate (HRS 78

/ESCRT0), ESCRT (I, II, III),

ALG- 2-Interacting Protein X (ALIX), and Syntennin- 1 46

(88). They play critical roles in membrane scission during ILV formation, and cargo selection, incorporate syndecans and other cargos into ILVs, and help in the formation or secretion of exosomes. The ESCRT associated pathways can also be involved in the formation of ILVs. Syndecan- synthenin and

Alix pathway and His-domain protein tyrosine phosphatase pathways **also** allocate **ESCRT III** 9

to form ILVs. Syndecan-synthenin and Alix can sort the proteins

**such as CD63, CD81, CD82, and CD9, and**	83

Fibroblast Growth Factor Receptor (FGFR) (89,90). The ESCRT- independent Pathway for the formation of ILVs involves components of lipid rafts such as ceramides. Ceramides actively participate in ILVs formation by playing an important role in membrane budding and curvature. Tetraspanins like CD63 and TSPN6 can also contribute to ILV formation independent of ESCRT. Chaperones like Heat Shock Protein 70 (HSP70) and HSC70, GPI-anchored proteins can co-sort cytosolic proteins into ILVs and help in the incorporation of lipid domains into ILVs respectively. Nucleic Acid Cargo Sorting: RNA cargo sorting: RNA cargo can be sorted through multiple pathways; wherein RNAs can be directly incorporated into exosomes due to the presence of particular sequence motifs (91), or can be incorporated with the assistance of RNA binding proteins such as

**RNA-Induced Silencing Complex (RISC) and Argonaute 2 (AGO2**	6

) (92), or through ESCRT-assisted RNA sorting, or with the help of RNA binding proteins sequestered within Tetraspanins enriched microdomains, or with the help of other proteins such as

**major vault protein and Y-Box- Binding Protein 1 (YBX1**	74

) (93). DNA cargo sorting: Even though protein cargo sorting and RNA cargo sorting have been extensively studied the knowledge about DNA cargo sorting into EVs is limited. Few recent studies have provided some insights into the potential mechanisms that might be involved in DNA cargo sorting. According to Yokoi et al. (2019) have reported that in ovarian cancerous cells, genomic DNA gets sorted into exosomes through the Tetraspanins into multivesicular bodies where micronuclei formed during cancer collapse releasing genomic DNA, which is then shuttled to Multivesicular bodies. Similarly, mitochondria also serve as a precursor for DNA cargo for exosomes. The PTEN-Induced Putative Kinase 1 (PINK1) protein released during mitochondrial damage helps in the interaction of mitochondria and multivesicular bodies leading to the sorting of mitochondrial cargo into MVBs (94). Knowledge about the involvement of ESCRT mechanism in DNA cargo sorting is lacking. However, some contradictory findings suggest that extracellular secretion of DNA is histones mediated and is exosome independent in nature (95). Future studies in this domain are

**essential for** enhancing **the understanding of** the mechanisms **involved in**	93

DNA cargo sorting into EVs. 2.2.2.3 Exosome release: Multivesicular bodies can attain secretory or degradative fate, MVBs destined to secretory face translocate towards

the plasma membrane and fuse **with the plasma membrane** marking **the** 56

end of exosome biogenesis. The exosome release involves the

well- known action **of Soluble N-ethylmaleimide-sensitive factor Attachment protein Receptors (SNARE) proteins** 40

mediating

membrane fusion events. **Fas/Fap-1/caveolin-1** 96

cascade,

long non-coding RNA HOX Transcript Antisense Intergenic RNA (lncRNA HOTAIR 68

) regulates the SNARE formation in stem cells and hepatocellular carcinoma cells respectively (96,97). The fate of MVBs is also greatly influenced by cytoskeletal elements -actin and microtubules which play crucial roles in transport, docking, and membrane fusion. Proteins like Rab27a, Rab 7, and Rab 31 play essential roles in stabilizing the docking sites, promoting exosome secretion, and interacting with motor cytoskeletal proteins. Additionally, divalent cations such as calcium ions

play an important role in the regulation of 7

Rab 11-mediated exosome secretion pathways (59,81,86). The biogenesis of Ectosomes and exosomes

is summarized in figure 1. Figure 1 : Extracellular vesicle biogenesis. **A** 89

. Ectosomes Biogenesis: Ectosomes are released upon membrane blebbing from the cells, which typically involves specific changes in

the lipid and protein components at certain sites **of the** plasma **membrane** 23

. During Ectosome formation, horizontal cargo sorting is followed by ectocytosis. B. Exosome Biogenesis: Exosomes originate from the endosomes on exocytosis of multivesicular bodies. The exosome biogenesis involves several key events such as Endocytosis, Early endosome formation, formation of MVB, ILV formation with molecular cargo sorting, Multivesicular body maturation, and exosome release. Endocytosis can be

clathrin-mediated or **caveolin-mediated or clathrin** or **caveolin- independent endocytosis. The clathrin-mediated** 28

endocytosis involves

nucleation, cargo selection, clathrin coat assembly, vesicle scission , vesicle formation, **and** 79

budding. Caveolae-mediated endocytosis begins with

the invagination of the plasma membrane 47

, rich in proteins like caveolins and cavins. The caveolae then pinch off from the membrane to form vesicles that transport cargo into the cell. Cargo Sorting, MVB Formation, and Exosome release: The molecular cargo within early endosomes gets selectively sorted into intraluminal vesicles forming

MVBs, which eventually **fuse with the plasma membrane to release** their contents as **exosomes into the extracellular space** 25

. Exosomes: The exosomes harbor diverse molecular cargo

including nucleic acids, proteins and lipids 26

. 2.3 EV Biogenetic Pathways: Bridging MASLD Pathogenesis: Careful observation of EV biogenesis pathways indicates that various molecular signatures overlap MASLD. For instance, CD53, a tetraspanin membrane protein involved in both EV biogenesis and immune function, has been shown

to be upregulated in hepatocytes following a **high-fat diet and** 8

inflammatory triggers. Inhibition of CD53 was found to prevent diet-induced fat accumulation and liver inflammation, highlighting its role in integrating metabolic and inflammatory signals in hepatocytes

and its potential as a therapeutic target for 10

conditions like MASLD and type 2 diabetes(98,99). Similarly, other components of EV biogenesis are implicated in disease pathogenesis and are summarized in Table 1. Table 1 3.1 MASLD - A historical Preview: 3.1.1 NAFLD-MASLD: Obesity, a major physical and physiological change, has been recognized as an important physiological event since prehistoric times. The prehistoric recognition of obesity is evident from the "Venus figurines" from the upper Paleolithic era, such as the Venus of Willendorf. While ancient civilizations, including those in Egypt and China, viewed obesity as a symbol of prosperity and fertility, the medical recognition of obesity began with the Indian physician Sushruta in the sixth century BC, who linked it to overindulgence and inactivity. European physicians and philosophers Hippocrates and Galens' views on obesity were highly influential in medieval and renaissance Europe. They emphasized diet and exercise as primary ways to manage obesity which continued to be practiced in medicine for centuries. Fatty liver disease was not identified as a distinct condition until the early 19th century (100). From 1975 to 2018, global obesity rates tripled coinciding with the introduction of food rich in high fructose corn syrup (101). Initially, the effect of these diet forms was directly linked with obesity however, it took a long time to confirm the role of such dietary regimens in metabolic syndrome-related diseases such as MASLD. Although there is historical evidence for fatty liver disease, recent understandings define the impact of obesity on fatty liver disease providing a crucial connection. MASLD is a condition identified recently that is significantly driven by obesity. While the ancients recognized and recorded obesity, its related conditions like MASLD reveal the long-standing consequences of obesity that can be traced back to historical observations and practices concerning weight and health. Obesity has been prevalent since prehistoric ages however, MASLD as a disease is quite overlooked by the medical community. Historical records suggest that the autopsy studies carried out during the 19th century revealed that hepatic steatosis was a common ailment affecting one-third of French and German populations, predominantly affecting women and tuberculosis patients (100). The earliest use of the term "fatty liver" dates to 1825, in Louis's textbook of anatomy and pathology. It was then Thomas Addison in 1836 who introduced the term "fatty liver," relating it to the presence of tuberculosis and alcohol consumption through histological differences (102). Much more emphasis was placed on understanding the mechanism of cirrhosis, as the initial liver manifestations leading to cirrhosis were not known at the time. Most diagnoses occurred at this advanced stage by 19th-century researchers, which ultimately led to the discovery that fatty infiltration in the liver, due to metabolic disorders or

alcoholism, causes cirrhosis. In the 1960s, "fatty liver hepatitis" emerged in German literature where the histopathological description of the liver with necroinflammation in obese individuals, distinguishing it from alcoholic steatohepatitis was made.

In 1980, Ludwig et al. used **the term non-alcoholic steatohepatitis (NASH** 72

) for the first time after inspecting liver biopsies of 20 patients showing similar traits like alcoholic steatohepatitis, including significant fat accumulation in the liver with signs of lobular hepatitis, focal necrosis, mixed inflammation, and often Mallory bodies, mostly in obese women with mild liver functional abnormalities and common fibrosis (103). The term NAFLD was introduced to hepatology by Fenton Schaffner in 1986 and the NASH progression to fibrosis and

cirrhosis was reported by Randall Lee (American pathologist) in 1989 (104,105). Recently **the term** 62

NAFLD was changed to MASLD and the NASH, now replaced with the term MASH) in early 2020s to better reflect the root cause for the disease, include cardiometabolic risk factors, and to reduce stigmatizing language associated with the words “nonalcoholic" and "fatty.". This change from NAFLD to MASLD was driven by global collaborative efforts led by

American Association for the Study of Liver Diseases (AASLD), European **Association for the Study of the Liver** (EASL), and **Asociación Latinoamericana para el Estudio del Hígado** (Latin American **Association for the Study of the Liver**) (ALEH) with **the** 17

Delphi proceedings to achieve the consensus among the experts from various fields (106,107). 3.1.2 MASLD diagnosis: The understanding and diagnosis of MASLD have significantly evolved over the past 5 decades. The earliest milestone in diagnosing this condition dates to the post-World War II era when it was observed that nonalcoholic individuals exhibit similar symptoms to those caused by alcohol. The drastic shift of research focus towards understanding MASLD progression and establishing diagnostic criteria happened in the 1990s leading to the development of histological grading and staging systems for MASH and to assess steatosis, ballooning, inflammation, and fibrosis (108). MASLD scoring systems were introduced in 1999 by the NASH Clinical Research Network with standardized methods and protocols to quantify the disease activity, stage, and fibrosis which essentially guided clinical trials and research (109). The genomic components of MASLD began to be elucidated in the early 2000s, with significant advancement in 2008 with the identification of the PNPLA3 gene as a key factor in increased hepatic fat content (110,110,111). This discovery highlighted genetic predisposition to NAFLD along with the subsequent discovery of influential genes such as TM6SF and GCKR (112–

114). The rising prevalence of MASLD has prompted the scientific community to focus on its diagnosis, bringing up several noninvasive diagnostic methods to the limelight between 2007 to 2015. Noninvasive diagnostic methods such as

NAFLD Fibrosis Score (NFS), Fibrosis-4 (FIB-4) index , and **vibration-controlled transient elastography** were developed **and** 37

became popular during this period (24,115,116). These noninvasive tools enhanced the ability to accurately diagnose advanced fibrosis without a liver biopsy. By 2015, fibrosis was identified as a crucial prognostic and diagnostic indicator in MASLD and was utilized for predicting overall and liver-specific mortality. Following 2020, the focus remains on refining and sensitizing the non-invasive diagnostic tools and developing advanced technologies aided with machine learning algorithms and accurate histological assessments. Several research consortia like

Liver Investigation Testing Marker Utility in Steatohepatitis (LITMUS) and Non-Invasive Biomarkers of Metabolic Liver Disease (NIMBLE 18

) are aimed towards identifying new biomarkers and validating these biomarkers for diagnosis of early MASLD stages like MASH to reduce the reliance of the whole diagnostic sector for MASLD on invasive liver biopsy procedures. However, despite all these recent developments in research of MASLD diagnostics,

liver biopsy continues to be the gold standard 86

for diagnosing MASH and early- stage fibrosis which emphasizes the need for further advancement in diagnostic methodologies. Historic preview of MASLD from prehistoric times to the present day is represented in figure 2. Figure 2: Historic preview of MASLD-

The understanding of MASLD pathology **and the development of therapeutic** 24

modalities for MASLD have evolved from prehistoric times to the present day. The historical overview of MASLD highlights the key milestones in understanding of the disease. 3.2 Guidelines for Diagnosis of MASLD: The diagnosis of MASLD is guided by protocols established by several prominent liver disease associations. These guidelines are produced by the EASL, the Asia-Pacific Working Party on NAFLD (APWP-NAFLD), the American

Association for the Study of Liver Diseases (AASLD), **the National Institute for Health and Care Excellence (NICE**), and **the Italian Association for the Study of the Liver (AISF** 16

). Each of these organizations provides comprehensive criteria and diagnostic tools, which are summarized in figure 3, offering a consolidated reference for clinicians to accurately diagnose MASLD. Figure 3: a.) Guidelines for MASLD diagnosis produced by

the European Association for the Study of the Liver (EASL), b.) **Asia-Pacific** 16

Working Party on NAFLD (APWP-NAFLD), c.)

American Association for the Study of Liver Diseases (AASLD), d.) **National Institute for Health and Care Excellence (NICE**), and e.) Italian **Association for the Study of the Liver** 16

(AISF). 3.3 MASLD diagnosis and challenges: MASLD is a silent non-symptomatic disease generally diagnosed through unintentional clinical or imaging tests. Though it is a slowly progressive disease with no symptoms during the early stage, fatigue, abdominal discomfort, and jaundice are the initial common indicators for suspecting the presence of disease (117). Besides, some of the

risk factors such as dyslipidemia, **obesity, insulin resistance** and **type 2 diabetes, metabolic syndrome** 63

, improper diet, physical inactivity, and sleep apnea make individuals more prone to the disease than the general healthy population (118). 3.3.1 Existing Diagnostic tools: Current diagnostic approaches for MASLD include various invasive and non-invasive techniques. 3.3.1.1 Non-invasive diagnostic approach- MASLD scoring systems using blood-based biomarkers: The diagnosis of MASLD usually starts with elevated liver enzyme levels, typically elevated

alanine aminotransferase (ALT) levels compared to aspartate aminotransferase (AST 52

). Using these enzyme levels as markers, along with others, like

gamma- glutamyl transferase (GGT) and alkaline phosphatase (ALP), provides **a** comprehensive 5 understanding **of**

the liver condition. Though ALT is liver-specific, it is altered in non-MASLD conditions too. Furthermore, due to low specificity, MASLD cannot be solely dependent on liver enzymes (119,120). Even though ALT as a biomarker is frequently used due to affordability and availability, its results depend upon overall liver functioning, and it may not exclusively indicate MASLD. For instance ALT has also been utilized in determining the metabolic syndrome(121). Similarly, several stage-specific and disease-specific molecular signatures for MASLD have been identified in the past decade. The noninvasive blood-based molecular signatures for MASLD with the sensitivity and specificity and their expression pattern in the disease with relevant studies are summarized in figure 4 and supplementary Table S1. Figure 4a: Biomarkers for MASLD: Sensitivity and specificity with the cutoff value for noninvasive blood-based biomarkers of MASLD Fibrosis- Adult population. Figure 4b: Biomarkers for MASLD: Sensitivity and specificity with the cutoff value for noninvasive blood-based biomarkers of MASLD Fibrosis- Pediatric Population. Figure 4c: Biomarkers for MASLD: Sensitivity and specificity with the cutoff value for noninvasive blood-based biomarkers of MASLD spectrum specific- Adult population. Figure 4d: Biomarkers for MASLD: Sensitivity and specificity with the cutoff value for noninvasive blood-based biomarkers of MASLD- MASH- Adult population. Figure 4e: Biomarkers for MASLD: Sensitivity and specificity with the cutoff value for noninvasive blood-based biomarkers of MASLD MASH- Pediatric population. However, the limitation associated with these biomarkers diminishes their reliability when used alone for disease identification. Therefore, either diagnostic scores or indices are usually used to diagnose the risk and degree of the disease, calculated by estimating the synergistic outcomes of clinical parameters and hematological parameters (122). The diagnostic indices are generally calculated based on the combination of specific panels pertaining to the organ of interest. The panel can be defined as a group of medical diagnostics tests generally recommended by physicians which can provide comprehensive information about a particular organ system, disease state, or function. The appropriate index for a disease state or condition is the one that considers a comprehensive range of diagnostic factors along with clinical parameters to assign a score. It ensures a more accurate and holistic assessment of the disease, considering various aspects of the patient's health and specific characteristics of the condition being evaluated. Indices and panels used to evaluate the MASLD risk are listed in Table 2. Table 2 Despite the availability of non-invasive scoring systems to evaluate the risk of MASLD, including the various common panels and indices listed in Table 2, many such statistically derived scoring systems have several limitations that prevent sole reliance on them for the assessment of MASLD. A major limitation associated with the scoring system is population heterogeneity, as these indices are generally calculated based on small, clustered populations in a hospital setting, and the actual disease prediction and risk evaluation become challenging. The lack of validation studies in different populations and the capability to specifically differentiate the risk of MASLD from other liver diseases pose a significant hurdle in this domain. Specificity could be achieved by the addition of MASLD-specific biomarkers to the scoring systems or panel which again limits the widespread utility of such panels or scoring systems. 3.3.1.2 Non-invasive diagnostic approach-Imaging techniques: The evaluation of liver health extends beyond the basic assessment of liver enzymes. In the absence of Hepatitis B, C, or other causes of chronic liver disease, elevated liver enzymes drive the clinician’s attention towards identifying the underlying conditions such as hepatic steatosis.

However, diagnosis of such conditions requires more than just clinical suspicion, as it demands an accurate and efficient way of evaluating the disease. In such a scenario, the evaluation is carried out with the help of imaging techniques or histological techniques. Imaging techniques primarily employed

include Ultrasound, Computed Tomography (CT), Magnetic Resonance Imaging (MRI), Magnetic Resonance Elastography (MRE), and 59

Transient Elastography (Fibro scan). The commonly employed imaging techniques for MASLD diagnosis are summarized in Table 3. The widespread availability of these noninvasive tools makes them go-to choices for initial screening and diagnosis. However, despite the advantages, they often have drawbacks. Notable drawbacks of imaging techniques involve operator dependency, non-interpretable results due to obesity, ascites, availability, operational cost, and sampling variability. Perhaps most crucially, they lack sufficient sensitivity to accurately identify MASH, which is characterized by inflammation and associated with risk factors such as fibrosis cirrhosis (123). Therefore, for a comprehensive analysis of liver health, it is important to combine the morphological and functional status of the liver with the appropriate clinical history. Table 3 3.3.1.3 Histological Techniques for MASLD: Histopathological studies involve invasive techniques like liver biopsy which proceeds with the excision of a piece of liver or liver tissue followed by the microscopic examination by the pathologist for signs of MASLD. Liver biopsy, an essential diagnostic tool for MASLD and MASH reveals distinct histological characteristics. In MASLD, steatosis is marked by the accumulation of fat droplets in the hepatocytic cytoplasm, which can be macro or microvesicular.

MASLD is also **defined** by **the presence of steatosis in** 41

at least 5% of hepatocytes. In addition to steatosis, MASH is also identified by hepatocellular ballooning and lobular inflammation. Ballooned hepatocytes, which indicate hepatocellular injury, lack caspase 9 and are linked to the activation of the hedgehog signaling pathway. Furthermore, Mallory-Denk Bodies (MDBs), which are cytoplasmic aggregates of keratins, ubiquitin, and p62, are not unique to MASLD and can also be found in other liver diseases. Lobular necroinflammation, primarily composed of mononuclear cells, is prominent in zone 3 and tends to decrease in cirrhosis. Other histological findings include enlarged mitochondria (megamitochondria), glycogenotic nuclei, and occasionally portal inflammation. Fibrosis

typically begins in zone 3 and **progresses** to **bridging fibrosis and cirrhosis** 71

, with pediatric cases often showing periportal fibrosis initially. These histological features are crucial for diagnosing and staging MASLD and MASH, providing valuable insights into disease progression and guiding treatment strategies (124). It is

considered a "Gold Standard" despite its demerits like invasiveness, sampling error, discomfort and pain of the patient, and limited frequency of monitoring (125). In the loop limitation, early detection of MASLD immensely contributes to the management along with the accuracy of findings. 3.3.1.4 Artificial intelligence in MASLD diagnosis (prediction, diagnosis): Recently, Artificial Intelligence (AI) has emerged as an effective tool for predicting and interpreting disease risk, presence of disease, and prognosis. AI is a broad field of computer science consisting of various technologies aimed at performing tasks that require human intervention and intelligence. Based on the technology, they can be categorized

into machine learning, deep learning, Natural Learning **Processing (NLP**), robotics, **and** computer vision. Machine **learning** 75

, a subset of AI enables the computer to make decisions based on the identification and data of the patterns rather than using technologies. This newly developed technique is widely used in radiological imaging, clinical diagnosis, medicine, risk stratification, and many more. AI can be directly or indirectly employed for the prediction or diagnosis of MASLD. AI plays a crucial role in developing machine learning models, followed by utilization of such machine learning models to predict disease risk which facilitates the designing of appropriate interventions for overcoming the disease. 3.3.1.4.1 Machine learning models for the analysis of reports and results: AI can also be utilized to develop tools for improving interpretations, transparency, and generalizability for increasing the efficiency of clinical decision-making. For ex, deep learning algorithms can enhance the automated interpretation of elastography, MRI, and CT scan results. An elaborate review on the utilization of machine learning approaches as new tools for histopathological diagnosis of MASH and MASLD is done elsewhere emphasizing more on the algorithms and machine learnings utilized for analysis of histopathological results and images (126). 3.2.1.4.2 Machine learning models for MASLD risk assessment and disease prediction: Machine learning models ease the statistical analysis when trained well. Thus, the machine learning models can accurately predict the MASLD risk, providing preliminary insights towards detailed targeted liver examinations. Investigation by Ma et al. involving 10508 patients, explored 11 machine learning algorithms to develop a diagnostic model for MASLD. They found Logistic Regression (LR) achieved 83.41% accuracy, while Support Vector Machine (SVM) excelled in specificity (0.946) and precision (0.725), and the AODE model exhibited the highest sensitivity (0.680). Utilizing the F-measure for analysis, the Bayesian Network (BN) model demonstrated the best performance, outperforming the Fatty Liver Index (FLI) by 9.17% in F-measure score, highlighting its potential for accurate MASLD diagnosis (127). Docherty et al. (2021) attempted

to develop a novel **machine learning model to predict** MASLD using **the** 82

data from NIDDK and Optum databases consisting of training an Extreme Gradient Boosting model (XGBoost). This model resulted in 0.82, sensitivity of 81%, and precision of 81%) in predicting MASH with high accuracy (128). A recent similar study used an AI

machine learning trained **XGBoost model to** predict **high-risk MASH** 19

using NHANES 2017 - March 2020 data which achieved high

sensitivity (0.82), specificity (0.91), accuracy (0.90), and AUC (0.95 19

), outperforming traditional biomarkers like

FIB-4, APRI, BARD, and MASLD fibrosis scores 19

(129). A recent approach to the identification of MASLD in patients with diabetes mellitus through machine learning approaches demonstrated high performance with the success rate of correctly identifying 82.24% (815/991) and 75.00% (586/744) MASLD (+) and MASLD (-) patients respectively (130). Similarly, Hassoun et al. recently developed NAIF (NAFLD-AI-Fibrosis), a novel AI-

based tool for accurate diagnosis of advanced **liver fibrosis in the general adult population** 60

which demonstrated superior sensitivity compared to traditional scoring methods such as APRI and Fib4 (stage F3/F4). NAIF achieved 72% precision, 61% sensitivity, and 77% specificity using data from the NHANES database (131). Machine learning models can be trained using XGBoost to detect the risk even in the absence of a few data sets. For ex, the application of explainable AI techniques in the medical field such as shapely additive explanation can improve the interpretation, transparency, and generalizability of machine learning models. Such models have enormous applications in medicine, as they facilitate clinical decision- making by converting clinical data into real-world applications. While the hurdles and challenges remain, the use of AI in diagnosis is promising and warrants further exploration in the future for its potential application in the medical field. Given the lack of efficient therapy for MASLD, early and accurate diagnosis is essential. Therefore, the availability of various non-invasive biomarkers and imaging techniques plays a significant role in the diagnostic process worldwide. 3.3.1.5 Resmetirom Phase 3 Trial: Spotlight on the Urgent Need for Surrogate Biomarkers in MASLD Resmetirom

liver-targeted thyroid hormone receptor-β selective drug 49

was recently approved by FDA as a new drug for MASH stage of non-cirrhotic patients. The drug has shown its

efficacy in reducing hepatic fat content, improving fibrosis and **MASH resolution, and** 49

reduction of liver damage(132). The drug has shown acceptable safety with mild to moderate common gastrointestinal adverse events. The long-term monitoring of the effect of drug in the MASLD population is essential to determine the effect of the drug on bone, thyroid and gonadal pathology. However, the clinical evaluation of effect of the drug relies heavily on noninvasive liver fibrosis assessments, yet current imaging techniques have several limitations, making it challenging for clinicians to make informed decisions, highlighting the urgent need for improved diagnostic markers(133). Hence, establishing universally accepted parameters will help maintain consistency among healthcare workers and researchers, thereby improving the quality of patient care. 4. Extracellular Vesicles in MASLD: 4.1 Role of EVs in MASLD: The liver, being the highly vascularized largest organ of

the human body, is exposed to large amounts of circulating antigens and 2

serves as frontline immune tissue (134). It is the primary organ responsible for removing circulating EVs, which are mainly eliminated by liver macrophages called Kupffer cells (135). Liver comprised of a heterogeneous population of cell types, including

hepatocytes, cholangiocytes, hepatic Stellate Cells (HSCs), liver sinusoidal endothelial cells (LSECs), Kupffer cells, and a range of **other immune cell populations, all** of which **secrete** 12

EVs (85,136–139). The number of EVs and molecular cargo that the individual EV carries greatly depends upon the physiological status of the cells and is altered under disease conditions (44–48). It is evident from recent studies that liver macrophages (138,140), hepatocytes (141,142), and HSCs (143) are involved in the disease pathology of MASLD. The pathophysiology of MASLD is a complex process which initiates with the primary manifestation of hepatocyte cell death, followed by a substantial accumulation of inflammatory cells in the affected area. The MASLD is markedly associated with obesity, elevated triglyceride levels, elevated ROS generation, oxidative DNA damage, impaired hepatic catalase activity reflecting the failure of the anti-oxidant mechanism ultimately leading to Lipoapoptosis (144), Necroptosis (145) or Pyroptosis (146) of hepatocytes. The oxidative stress in MASLD is often associated with elevated

activation of inflammatory pathways such **as the c-Jun-N-terminal kinase (JNK** 64

)/NFκB (147). Liver cells are known to release EVs both in healthy individuals and liver patients. The pathophysiological cascade initiated with liver damage significantly alters the nature, composition, and functional properties of the EVs produced by the cells. Since EVs are involved in intercellular communication, structural damage to the parent cells alters EV communication. The EVs released from the damaged liver will communicate with surrounding cells and influence the microenvironment within the liver. The inflammatory cells accumulated at the affected pathological site also cause a significant change in the EV pool by producing many inflammatory EVs in the affected area. The earliest evidence of EVs as disease indicators came from the study of an animal model

fed with a **high-fat diet** which showed a **significant increase in the** amount **of** 73

circulating EVs. This study further provided primary evidence for the reflection of the severity of steatohepatitis in mice with increased circulating EV concentration (148). Furthermore, the apoptotic bodies released by the hepatocytes increase the expression of death receptor ligands in Kupffer cells inducing apoptosis of hepatocytes leading to inflammation and fibrosis (149). Similarly, an in vitro study revealed that the application of membrane-bound microparticles derived from murine or human hepatocytes exposed to lipotoxic stress on endothelial cells demonstrated that these particles were proangiogenic in nature which increases the severity of steatohepatitis (150). Although the role of macrophages in exacerbating the disease is well known, the precise role of EVs in disease remained unclear until a study by Kakazu et al. which shed light on the connection. They elucidated the role of EVs in bridging these gaps. Accumulation of saturated fatty acids such as palmitate, a precursor of ceramide, a lipotoxic lipid, leads to ER stress, a commonly observed condition in diseases like MAFLD. These EVs are enriched in C16:0 ceramide and stimulate macrophage chemotaxis via sphingosine-1-phosphate (S1P) generation. Increased levels of C16:0 ceramide-enriched circulating EVs are observed in both mice and human NASH, suggesting their potential as bioactive biomarkers (151). Several such studies have shown the role of lipotoxic hepatocyte-derived EVs in aggravating inflammation. For instance, a study on hypoxia condition in a fat-laden hepatic cell

by stabilizing hypoxia- inducible factor 1-alpha (HIF-1α 80

),

showed a significant increase in the number **of** EVs released. **The** 31

hypoxia-induced promoted inflammatory signals and contributed to enhanced EV secretion. In addition to this, when the EVs obtained from hypoxic fat-laden tissues were used to treat Kupffer cells, there were phenotypic occurrences of hypoxic conditions in Kupffer cells suggesting the impact of EVs in disease development through crosstalk (152). The analysis of

serum extracellular vesicles by Sakane et al., 2024 revealed the presence of proteomic signature Fibulin 3 correlation with the liver-related events including MASLD and Fibrosis (49). Furthermore, MASLD

is associated with elevated levels of inflammatory cytokines including IL-6 and 65

TNF-α (153). The elevated inflammatory cytokines influence the release and composition of hepatocyte derived EVs (154). As mentioned previously these EVs can exacerbate liver inflammation and promote the apoptosis in surrounding cells. The dysregulation of autophagy followed by apoptosis increases lipotoxicity. The role of EVs in autophagy in MASH is elaborately explained elsewhere (155). Muscle and liver play crucial regulatory role in metabolism, working together to exhibit the key metabolic functions such as maintenance of energy balance and regulation of glucose and lipid levels. The dysfunction in any of these can aggravate the other, leading to a vicious cycle of muscle and liver deterioration. MASLD is also intricately interlinked with muscle pathology, sarcopenia in particular. MASLD and Sarcopenia can coexist in both obese and nonobese individuals (156,157). Sarcopenia and obesity-associated MASLD share common pathological manifestations including muscle loss, metabolic dysregulation, inflammation, and insulin resistance. The lean MASLD patients experience muscle wasting due to altered inflammatory and metabolic signaling. Evidence suggests that the EVs contribute significantly to the deterioration of skeletal muscles in sarcopenic conditions. The molecular payloads of such EVs can exacerbate the key pathological events including inflammation, protein degradation, and mitochondrial functions leading to muscle atrophy and impairment of muscle regeneration (158,159). EVs likely contribute to muscle wasting in non-obese MASLD through different mechanisms compared to obesity-associated MASLD. EVs in obesity-associated MASLD contribute to inflammation, insulin resistance, and lipid accumulation in muscles while EVs in nonobese MASLD contribute to oxidative stress, impaired metabolism, and muscle regeneration. Similar to MASLD a significant limitation associated with the Sarcopenia is lack of diagnostic markers to support the clinical investigations (160). Recognition of these profound differences in EV's role in disease pathology could aid in understanding the disease pathology and identification of new molecular signatures for the disease. 4.2 EVs as potential biomarkers The accumulated evidence in the past indicates that EVs are instrumental in the pathogenesis of MASLD, underscoring their significance as biomarkers for disease evaluation. Several studies have specifically identified EVs as promising biomarkers for MASLD. 4.2.1 TRAIL-enriched EVs: A study on lipid-induced signaling aggravating inflammatory response through EVs enriched with

Tumor necrosis factor-Related Apoptosis-Inducing Ligand (TRAIL) released from hepatocytes **has** 36

demonstrated that Lipotoxic stress induced by lipids such as palmitate and Lysophosphatidyl Choline (LPC) enhances the EV secretion which interns activates the inflammation by inducing macrophage activation. The study also demonstrated that inhibition of EV release ameliorates the NASH in murine models. The study essentially highlights the importance of TRAIL-enriched EVs as a potential biomarker unfolding a novel therapeutic target (inhibition of ROCK1-dependent release of EV by

hepatocytes) for NASH (161). 4.2.2 ITGβ1-enriched EVs: Monocyte-derived macrophages infiltrated into the liver contributing to the inflammatory response in NASH (MASH). Guo et al. in 2019 showed that EVs enriched with Integrin Beta-1 (ITGβ1), released from LPC-treated hepatocytes

mediate monocyte adhesion and promote liver inflammation in a murine model 4

for NASH. They used hepatocytes treated with either vehicle control or LPC for EV isolation and proteomic analysis. Then the diet-induced NASH murine model was treated with anti-integrin β1 (ITGβ1) neutralizing antibody (ITGβ1Ab) or a control IgG isotype. The finding suggested presence of a new biomarker for NASH. The study also emphasizes that EVs derived from the hepatocytes enriched with ITGβ1, regulate NASH inflammation and antibodies against ITGβ1 ameliorate NASH in diet-induced murine models for NASH, suggesting a potential anti-inflammatory therapeutic strategy for NASH (142). 4.2.3 S1P-enriched EVs: Hepatocyte lipotoxicity leads to inflammatory macrophage effector responses during NASH. A study on EVs released from palmitic acid-treated hepatocytes revealed that they are enriched with Sphingosine-1-Phosphate (S1P) and are involved in recruiting macrophages to the liver. The study also demonstrated that the EV S1P enrichment largely is influenced by the activity of enzymes upon sphingosine kinases 1 and 2 and the pharmacological inhibition of these enzymes alleviated EV cargo enrichment and concomitant macrophage recruitment inferring that the Sphingosine-1-Phosphate (S1P) enriched EVs could be a potential biomarker and therapeutic target for NASH (162). 4.2.4 Hepatic Stellate Cell (HSC)-derived EVs: Hepatic fibrosis involves the excessive accumulation of extracellular matrix leading to scar formation in the liver mediated by activated HSCs under lipotoxic stress. Lipotoxic stress elevates the secretion of exosomes carrying micro-RNAs by hepatocytes, which upon internalization can activate HSCs to undergo proliferation and migration. Internalization of EVs not only elevates the proliferation migration of HSCs but also mediates the

expression of profibrotic factors (Transforming Growth Factor-beta (TGF-β 44

), Cellular Communication Network 2 (CCN2),

collagen type 1 , and **Alpha-Smooth Muscle Actin (α-SMA** 20

) (163,164). Isolation and characterization of such exosomes open a new diagnostic opportunity for monitoring the progression towards fibrosis in MASLD. Similarly, recent studies on EVs secreted by healthy EVs have revealed that these EVs can inhibit the progression towards fibrosis largely by alleviating the activation of HSCs or by suppressing the inflammatory pathway (165). 4.2.5 Liver Sinusoidal Endothelial Cell (LSEC)-derived EVs: Upon chronic liver diseases such as NASH fenestrated lining of liver arteries and veins lose their discontinuity due to de- differentiation of LSECs. LSECs form fenestrated linings in arteries and veins of the liver. A study on transcriptomic analysis of LSECs demonstrated that the EVs

secreted by the LSECs were potent angiocrine effectors and showed a deactivating effect on HSCs. The study also revealed several stage-specific proteomic signatures of EVs in chronic liver diseases unraveling new therapeutic targets and potential biomarkers (166). The accumulating literature clearly identifies tiny EVs as promising vehicles carrying enormous cellular information that could be potentially exploited as biomarkers for MASLD. The unique characteristics of EVs and potential advantages over the existing biomarker highlight the importance of EV's potential in diagnostics. The diagnostic potential of EVs has been clearly illustrated by a recent investigation by Jiang et al. on plasma exosomal metabolites derived from MASLD patients with impaired fasting glucose. The investigation revealed that the exosomes derived from the patients had elevated levels of fatty acids, including linoleic acid, palmitate, ceramide, and oleamide in their exosomes, and reduced phosphatidylethanolamine (PE). The detailed pathway analysis elucidated altered linoleic acid metabolism as a characteristic feature of MASLD with impaired fasting glucose. The findings clearly supported the fact that alteration of specific lipid components on EVs clearly reflects the early metabolic dysfunction, providing valuable biomarkers for diagnosis of disease progression(167). EVs offer many advantages over existing biomarkers as they can increase the sensitivity and specificity of non-invasive diagnostics, stability, and long-term storage of EVs, enabling the window period to develop standardized protocols and procedures for analysis. The diversity of EV molecular cargo offers a new benefit as it can potentially be utilized for multiparametric diagnostic analysis. The recent development in EV research involving standardization of EV analysis protocols provides hope for developing new diagnostic strategies for MASLD using EVs. Despite limitations such as lack of technical advancements for thorough analysis of EV molecular cargo, rapid progress promises a promising future for EVs in diagnostics. The swift advancements of multi-omics approach in analyzing EVs and continuous research outputs in this domain suggest that EVs could play an essential role in next-generation diagnostic techniques offering more precise early detection of MASLD. 4.3 Challenges in Utilizing EVs for MASLD Diagnostics: 4.3.1 State of EV-based Diagnostics for MASLD: The studies on EV based diagnostics are largely in state of proof-of-concept phase, with most of the studies identifying the molecular signatures through omics approaches(168). While these studies only suggest certain molecular species are expressed differently in MASLD translating this knowledge into clinically relevant methods remain challenging. 4.3.2 Selection of EV subtypes and Isolation of EVs: A key challenge in EV diagnostics is selection and isolation of EV subtypes. EVs display heterogeneity in size, composition and function. Isolating specific subtypes of EVs is crucial, especially for evaluation of disease specific molecular markers. For instance, isolation of liver specific EVs from biological fluids is essential for accurate disease profiling in MASLD. While liver-specific markers like ASGR (Asialoglycoprotein Receptor) protein can assist in isolating liver-specific EV populations(169), incorporating this step would add further complexity to the overall EV isolation process. EV isolation techniques like Size Exclusion Chromatography (SEC) and Ultra-Centrifugation (UC) are widely used, but they have notable drawbacks(170). SEC separates EVs into different size fractions, so choosing the right fraction is crucial. Focusing on one fraction risks missing important molecular signatures in the others. Similarly, UC can cause EV rupture leading to loss of molecular cargo. Even though bulk precipitation methods are cost effective, they can often introduce several other contaminants further complicating purity and accuracy. 4.3.3 Challenges in EV Data and Population Studies: Existing databases such as ExoCarta and Vesiclepedia provide some basic information about EVs, however extensive population specific information as seen in genetic databases is lacking. Similarly, global data on EV cargo across different geographic and ethnic populations is lacking, necessitating extensive validation for EV-based markers. 4.3.4 Knowledge Gaps in EV Biodistribution and Circulation: Factors such as

physiological state of patients, time and day are understood poorly. Understanding these dynamics is essential for reliable diagnosis. 4.3.5 Need for Clinical Validation in large cohorts: Despite large amount of evidence from invitro studies, there is a need for validation of these findings in patients derived samples in order to understand complex human physiology across various diverse populations before moving into clinical settings. 5. Exploration of EVs for the MASLD therapy: The existing therapeutic modalities for MASLD is discussed elaborately elsewhere (171), and all the developing therapeutic modalities that are being tested as a clinical trial are summarized in the Table 4. The clinical trial data clearly suggests that the drugs being tested are specific for a few stages of MASLD. While most small molecules (drugs) developed address the reduction of inflammation, fat accumulation, and scarring caused due to fibrosis other interventions developed focus on lifestyle modifications, such as diet, exercise, and probiotics. There is a need for precise stage- specific therapeutic modalities for increasing the life expectancy of individuals with MASLD. These synthetic drugs and suggested lifestyle modifications can improve the patient's conditions by retarding inflammation and preventing further accumulation of fat in the liver. Contrary to this, synthetic drugs do pose challenges in attaining the target affecting non- targeted cells, tissues, or organs causing unwanted side effects. Several siRNA-based therapeutic modalities developed for MASLD, even though they show higher efficacy, failed to translate into clinics. This is in part due to the failure of the delivery of therapeutic cargo to a suitable action site. Similarly, the re-establishment of cellular physiology in the affected liver is possible only when the cellular components of the damaged tissue microenvironment are regenerated. Although the liver is an organ with the highest regeneration capacity, the functional retardation of cellular components of the liver during MASLD reduces the regeneration. These key challenges bring biotherapeutics into the limelight in MASLD therapeutics. Table 4 EVs as a therapeutic modality is one of the most promising strategies that is gaining importance as an answer to conquering diseases by providing an effective way of specifically treating disease alone. These membrane-bound naturally produced lipid nanoparticles can protect the molecular cargo from degradation in the biological environment. The inherent property of EVs as cargo carriers can be utilized for delivering therapeutic cargo to the site of action. The diverse range of molecular payloads, including proteins, lipids, microRNAs, and nucleic acids, is selectively, actively, or passively encapsulated in EVs, influencing their functionality. Moreover, EVs derived from stem cells inherently possess the potential to activate cellular regeneration. EVs harbor unique protein barcodes on their surface and can acquire definite bimolecular corona depending on their surroundings that enable them to interact with the specific organ of interest or receptor of interest. Nucleic acids, chemotherapeutic drugs, small molecules, and even viruses can be essentially packed inside the EVs and delivered to a targeted site. EVs are becoming therapeutic vehicles of great importance due to their biocompatibility, reduced immunogenicity, ability to cross biological barriers, versatility in cargo loading, and many more. Next-generation EV therapeutics aim to utilize EVs fortified with therapeutic cargo or drugs for delivering it to the target of interest therapy off-target effects. The inherent characteristics of EVs with their cargo-specific therapeutic effects along with the site-specific actions due to protein barcoding make them suitable candidates for the development of therapeutic modalities for any disease. EVs as therapeutic modalities can be utilized in two different ways, as delivery vehicles and as therapeutic agents. Based on these functions they can be briefly classified into two different classes namely Naturally occurring EVs and Engineered EVs. The naturally occurring EVs bear endogenously packed cargoes derived from parent such as immune cells, and mesenchymal stem cells, bearing inherent therapeutic potential due to their origin whereas artificial/ engineered EVs are the EVs that are altered through surface modifications through biological, or chemical methods, subjected to physical or

biological treatments for loading cargo of interest. Though there are multiple ways of loading materials onto the EVs, the selection of method is crucial in ensuring efficient loading. It

**depends on the physicochemical properties of** cargoes, source **of** cargo, **and**	39

EV subtype. The effectiveness of EVs as a “delivery vehicle” and EVs as a “therapeutic agent” is diversely supported by studies of different diseases. EVs naturally produced by the cells harbor enormous cellular components that aid their therapeutic value. The majority of MSC-derived EVs are being therapeutically employed for their potential immunomodulation or immunoregulation and regeneration. As of 2024, there are a dozen clinical trials ongoing wherein EVs derived from MSC have been employed for immune regulation and regenerative medicine. The therapeutic utility of MSC-derived EVs has been reviewed in detail elsewhere with relevant ongoing clinical trials (172). The role of MSC- derived EVs as therapeutic agents in treating liver diseases is being thoroughly investigated and the clinical trials registered to employ EVs for liver-related disorders are listed in Table 5. Table 5: The role of MSC-derived EVs as therapeutic agents in treating liver diseases Register Title Phase Status Condition Intervention No. NCT05940 The Safety and Phase1/ Withdrawn Acute-on- MSC-EVs 610 Efficacy of MSC- Phase 2 chronic liver EVs

**in Acute** / failure **Acute-on-Chronic Liver Failure** NCT05881 MSC-EV **in**	16

Phase1 Withdrawn Acute-on- MSC-EVs 668 Acute-on-Chronic chronic liver Liver Failure failure after After Liver Liver Transplantation Transplantati on NCT05871 Effect of Phase 2 Recruiting Decompensat MSC-derived 463 Mesenchymal ed liver exosomes Stem Cells- cirrhosis derived Exosomes in Decompensated Liver Cirrhosis

**MSC-EVs, Mesenchymal stem cells-derived extracellular vesicles** 5.1 **EVs**	13

as potential therapeutic agents for MASLD: Although there is limited evidence for EVs as therapeutic modalities for treating MASLD, the accumulating evidence on utilization of EVs for therapeutics and delivery systems in several liver diseases and invitro models resembling MASLD suggests the scope of EV therapy for MASLD. 5.1.1 EVs in Hepatic steatosis and Inflammation therapy: 5.1.1.1 MSC-derived EVs for Hepatic steatosis and Inflammation: Mesenchymal stem cell- derived EVs from various stem cell sources have shown their anti-inflammatory and regenerative effects which can be utilized as a therapeutic strategy for treating Hepatic steatosis and inflammation. Hepatic steatosis and inflammation are the key pathological events occurring in the initial stages of MASLD which progresses towards severe stages such as fibrosis. Several studies have been conducted assessing

the ability of MSC-derived EVs to tackle Hepatic steatosis **and** 84

inflammation. As described in previous sections EVs derive their molecular cargo and functionality from the parent cells. A study on exploring the therapeutic utility of microRNA-136-5p of

EVs derived from mice **bone marrow** -derived **mesenchymal cells** demonstrated **the** inhibition **of** 76

GNAS/STAT3 signaling pathway and Lipopolysaccharide (LPS)-induced inflammation resulting in reduced liver inflammation and enhancement of M2 macrophage polarization through the GNAS-mediated PI3K/ERK/STAT3 axis in an animal model with chronic liver damage induced by carbon tetrachloride (176). A similar study on

exosomes derived from human umbilical cord- mesenchymal stem cells (hUC-MSCs) demonstrated efficacy **in** mitigating. The **study** 61

emphasized the influence of MSC-derived exosomal miR-24-3p in reducing lipid accumulation, oxidative stress, and inflammation, leading to improved hepatic function and decreased steatosis in both palmitate- treated mouse hepatocytes invitro and

a high-fat diet-induced NAFLD mouse model 30

invivo. miR-24-3p excreted these protective effects by targeting

Kelch-like ECH-Associated Protein 1 (KEAP-1) signaling 81

, thereby attenuating hepatic lipid metabolism disturbance, inflammation, and oxidative stress (177). A study on direct employment of

human umbilical cord mesenchymal stem cell (hUC-MSC)- derived exosomes for their **therapeutic potential** 35

in nonalcoholic steatohepatitis (NASH) using an MCD-induced mouse model revealed that the intravenous transplantation of hUC-MSC exosomes improved body weight loss and liver damage induced by MCD in mice. Furthermore, it also reduced inflammatory cytokines in liver tissue and induced anti-inflammatory phenotypes in macrophages. Macrophage polarization was evident in

in vitro and in vivo experimental models. The 54

exosomes were also capable of reversing the downregulation of PPARα protein expression in ox-LDL-treated hepatocytes in vitro and in vivo NASH mouse liver (178). The potential application

of human placenta-derived MSCs extracellular vesicles (hPMSCs- EVs 32

) in liver regeneration following hepatectomy was investigated in 2022 by Li et al. Intravenously administered hPMSCs-EVs before partial hepatectomy could potentially

improve liver regeneration in vivo and hepatocyte **proliferation in vitro** 32

. The findings suggested that hPMSCs-EVs hold the potential for preventing hepatic dysfunction and improving liver regeneration, possibly through circ-RBM23 delivery (179). Similarly, a study on the delivery of inherent RNF31 through EVs derived from mesenchymal stem cells showed that the RNF31 delivery significantly improved liver function by alleviating

hepatic steatosis in high-fat diet- fed 30

mice (180). 5.1.1.2 Non-MSC EVs for Hepatic steatosis and Inflammation: Literature evidence suggests that breastfeeding reduces the risk of MASLD/NAFLD. A study on the effect of EVs derived from mothers' milk on mouse NAFLD models

and primary hepatocytes treated with free fatty **acid** 30

showed that the breast milk-derived EVs alleviated

hepatic steatosis and insulin resistance **in** NAFLD **mice by inhibiting** lipogenesis **and** 30

promoting lipolysis. These effects are likely due to EV cargo (proteins and miRNAs) related to lipid metabolism, suggesting a new therapeutic strategy for NAFLD treatment (181). Exosomes derived from Stem Cells of the Apical Papilla (SCAPs) also have shown significant therapeutic potential for treating

non-alcoholic steatohepatitis (NASH) in **a Methionine-Choline Deficient (MCD) diet** -induced **mouse model** 67

. A study on these exosomes demonstrated that the administration of SCAPs-derived exosomes led to reductions in liver damage and hepatic fat accumulation and improved lipid metabolism through the upregulation of p-AMPK and mitochondrial biogenesis factors (182). Immune cells play a key regulatory action in exhibiting an immune cascade. Macrophage polarization differentiation of macrophages to attain different phenotypes is an important event in inflammatory and anti-inflammatory reactions. M2 macrophage-derived exosomes loaded with siRNA targeting RIPK3 demonstrated significant reductions in pro-inflammatory cytokines, improved liver pathology, and balanced Th17/Treg cell ratios in a mouse model of immune hepatitis. These findings suggest that EVs can effectively deliver therapeutic agents to liver cells, offering a potential strategy for treating NAFLD (183). 5.1.2 EVs in Fibrosis Therapy: 5.1.2.1 MSC derived EVs for Fibrosis: EVs, a paracrine effector of MSCs, can be employed for the regeneration of the hepatic cell population for recovery from fibrosis. MSCs have been already explored as a therapeutic modality for various fibrotic conditions including pulmonary fibrosis, spinal cord injury, scarring, and organ transplantation. Recent studies on MSC-derived EVs in treating liver fibrosis unravel MSC-derived EVs as potential therapeutic agents for liver fibrosis. A study on the efficacy

of human Amniotic Mesenchymal Stem **Cell-derived Extracellular Vesicles** (AMSCs- **EVs** 21

) to treat hepatic fibrosis elucidated that the

AMSCs-EVs delivered miR-200a into hepatocytes 55

suppressing ZEB1/PIK3R3 axis. Suppression of the ZEB1/PIK3R3 axis reduced hepatic fibrosis by inhibiting its anti-fibrotic-effect (184). A similar study explored the utilization of

Wharton's jelly mesenchymal stem cells (hWJMSC- Exo 31

) derived exosomes for improving liver function and regeneration during liver fibrosis. The study demonstrated delivering

miR-124 via **exosomes from human Wharton's jelly mesenchymal stem cells (hWJMSC-Exo** 31

) to improve liver fibrosis. The exosomes enriched with miR-124 could significantly reduce

inflammation and collagen accumulation. The fibrotic 27

inflammatory markers

IL-6, IL-17, TGF-β, STAT3, α-SMA , COL **in** 27

a CCl4-induced mouse model, were downregulated significantly after administration of

miR-124-enriched exosomes (ExomiR-124). The 27

study also demonstrated that the ExomiR-124 also promoted the shift

of splenic monocytes from inflammatory to restorative 27

phenotypes confirming ExomiR-124 as a promising anti-inflammatory and anti-fibrotic therapeutic option for liver fibrosis (185). A study investigating the therapeutic efficacy of MSC-ectosomes and medical ozone on CCL4- induced liver fibrosis rat model showed superior efficacy of EVs over ozone in reducing liver enzyme levels, oxidative stress markers, and histological liver damage. The evidence given by the study highlights the therapeutic potential of MSC-MVs and the authors also conclude that future research is needed to optimize dosages and explore combined therapies for enhanced effectiveness (186).

The anti-fibrotic effect of human- **derived EVs** was supported **by** 55

another study where

EVs derived Human Liver Stem Cells (HLSCs) **were** 91

evaluated for treating NASH, a stage in MASLD spectrum in immunocompromised mice. EV treatment significantly

reduced signs of liver fibrosis and inflammation and 50

downregulated 28

out of 29 fibrosis-associated genes upregulated in NASH liver 50

(187). Human placental mesenchymal stem cell-derived exosomes (ExoMSC) employed for treating liver fibrosis showed an effective reduction of fibrosis and improvement of liver microenvironment in a PSC mouse model and patient-derived organoids autoimmune diseases by inhibiting Th17 differentiation and reducing ER stress. ExoMSC were capable of downregulating IκBζ expression and PERK/CHOP signaling suggesting potential therapeutic applications of ExoMSC for PSC and Th17-related liver diseases (188). 5.1.2.2 Non-MSC EVs in Fibrosis: Semaglutide, a GLP-1 receptor agonist, shows promise in treating MASLD associated with T2D by modulating exosome composition. In a study with T2D patients, responders to semaglutide treatment exhibited significant improvements in liver fibrosis markers, as their exosomes reduced stellate cell activation and fibrosis-related protein expression. This suggests semaglutide’s therapeutic potential through indirect mechanisms involving exosome-mediated cell signaling, though the exact pathways require further investigation. Even though exosomes or EVs are not directly employed for the therapy the findings underscore the importance of exosome research in developing MASLD therapies (189). Curcumin's therapeutic potential is hampered by its hydrophobicity and low bioavailability. small Extracellular Vesicles (sEVs) offer a promising solution for curcumin delivery due to their favorable size, composition, and non-immunogenic properties. This study investigates curcumin-loaded milk sEVs, using passive and active (saponin-assisted) loading methods, which maintain nanoparticle integrity and size. Active loading achieved significantly higher curcumin encapsulation. In vitro tests showed higher cytotoxicity in cancer cells versus primary hepatocytes, while in vivo studies demonstrated reduced liver damage and fibrosis in a liver fibrosis model, highlighting sEVs as effective curcumin delivery systems (190). 5.2 Advances in EVs therapy for MASLD: 5.2.1 Targeted delivery for MASLD therapy: Surface decoration of EVs alters the EV uptake by the cells, which can be utilized for targeting

the EVs at specific sites. The EVs can be surface engineered to achieve specific targeting. A detailed review of extracellular vesicle targeting to non-parenchymal cells for tackling liver fibrosis is given elsewhere (191). EVs decorated with ligands of interest or targeting moiety produced either by direct modification or parent cell modification are utilized for targeting the specific receptors, cells, or tissues. Notably recently several preclinical evidence experimentally demonstrated the importance of targeting the extracellular vesicles for precise delivery of therapeutic cargo eliminating off-target effects and enhancing the efficacy of the therapy. The study on the attenuation of Hepatic steatosis by Yu et al employed a unique targeting strategy for delivering pirfenidone-laden EVs to HSCs. The study demonstrated that the Hyaluronic Acid modified EVs carrying pirfenidone to HSCs, showed superior efficacy in inhibiting HSC activation and reducing collagen synthesis in both rat HSC-T6 cell line and BRL cell line. Furthermore, the study also demonstrated that in the murine hepatic fibrosis model the therapeutic strategy could significantly improve the hepatic cell morphology and amelioration of hepatic fibrosis(192). Similarly, HSTP1 peptide fused with exosomal membrane protein Lamp2b through genetic engineering was employed for targeting the HSCs for effective reversal of fibrosis by

Human umbilical cord Mesenchymal Stem Cell (Huc- MSC)-derived exosomes (193). **In** 33

a similar study

EVs from human Adipose-Derived Stem Cells (ADSCs) **were** 1

exogenously modified to bear vitamin A on their surface and showed enhanced targeting towards HSCs. The exogenously modified EVs were capable of reversing fibrotic cascade even at 10-fold lower doses compared to the antifibrotic effects of bare EVs (194). Screening of targeting peptides, ligands, or chemical compounds for evaluating efficacy and specificity provides the ideal candidates for the targeting of a particular receptor. Such ideal targeting moiety can be further employed for targeting specific hepatic cellular niches. The knowledge about MASLD pathology provides essential grounds for the identification of a suitable cellular population to be targeted for the effective reversal of the disease. Future EV therapeutics rely on the development of essential loading strategies for loading molecular cargo and targeting the EVs to the suitable hepatic cellular niche. Advancements in the targeting and loading strategy essentially enhance EV's therapeutic potential for conquering MASLD. 5.2.2 Hybrid EVs for MASLD therapy: The efficacy of EVs can also be enhanced by coupling EVs with other nanocarriers to enhance therapeutic potential. Internalization of EVs is generally driven by stereochemical factors of cellular membranes. Anionic components of cellular membranes often offer electrostatic repulsion towards the exosomes affecting the EV internalization. Sato et al. developed a biological nano transporter hybrid by the fusion of liposomes and EVs. The hybrid biological nano transporter aided with PEG surface modifications improved interaction with the target cells facilitating efficient delivery of therapeutic cargo (195). Similarly, Piffoux et al. and Mukherjee et al. demonstrated enhanced drug delivery efficiency by mixing MSC-derived EVs with PEGylated liposomes, suggesting a promising strategy for improving drug delivery systems (196,197). Evers et al. compared the physicochemical properties and functionality of

liposomes with hybrid nanoparticles. An effective delivery vehicle was created by fusing engineered EVs with liposomes, exhibiting greater efficacy compared to liposomes alone (198). Similar studies on hybrid EVs

revealed that EVs **in combination with other** nano-carriers can **increase the** efficacy **of** 77

carrier systems. However, they also pose several hurdles as efficient cargo carriers; stability issues and natural heterogeneity of EVs, reproducibility, and consistency make the way of this carrier system towards clinics difficult. However, when these limitations are properly addressed a nanocarrier system coupled with surface-engineered EVs for efficient targeting when utilized can effectively enhance therapeutic potential. 5.3 Challenges in EV therapeutics: In conclusion, EVs are emerging therapeutic measures for MASLD both as cargo carriers and therapeutic agents. However, the emerging EV therapeutics pose considerable challenges to be translated into clinics. Major challenges associated with EV therapeutics are maintaining the consistency of the therapeutic products as they are biogenic in origin. The heterogeneity of the EV population also creates the need for efficient isolation of clinical-grade EV subpopulations. Producing high-quality EVs with consistent properties remains a significant hurdle as it requires scalable manufacturing and reproducible manufacturing processes. The significant strides in improving bioengineering EVs in the past 5 years and efforts to optimize the manufacturing of EVs for clinics poised to overcome current challenges paving the way for a reliable EV-based therapy for MASLD. The current trend and rapidity in EV research ensure that these challenges will soon be addressed, resulting in the production of effective EV-based therapeutic measures for MASLD. This rapid progress holds promise for significantly improving patient outcomes by providing precise, targeted, and efficient therapeutic strategies for MASLD. 6. Strengths and limitations: This review consolidates a substantial body of research work carried out in the past on EVs and MASLD making it a literature resource for clinicians and researchers. Key topics regarding EVs and MASLD are simplified with the help of tables and figures to grasp the interaction of EVs in MASLD easily. This study included multiple clinical trials and the concerning issues surrounding MASLD for validating the claims and to ensure its timely relevance. The comprehensive tables aid in the practical application of this knowledge in clinical settings. By presenting both the positive and negative aspects of EVs in diagnostics and therapeutics without bias,

this review provides an objective **overview of the** current state **of** 13

research, which will help researchers identify existing knowledge gaps and potential areas for future investigation. This review provides a broad perspective on the disease pathology of MASLD along with other liver diseases, addressing clinical trials and the role of EVs in disease manifestation. Due to the limited availability of EV-related studies specific to MASLD in the current literature, other liver diseases were also explored with overlapping pathological features. This approach is based on the rationale that MASLD shares several similar pathological events with other liver diseases, allowing insights into the potential utility of EVs in MASLD by analogy. However, a more focused bioinformatic study would help synthesize precise evidence regarding EV-based diagnostic markers specifically for MASLD. 7. Conclusion and Future Prospective: EVs are one

such newly found exploring research field of science with continuous evolution where there is significant research ongoing in the last 20 years, peaking in 2016 with 20.47% of the total articles from the entire period (199), growing rapidly with much wider and more practical applications for systematic problems of the population. EVs as a therapeutic modality are also gaining significant attention, as demonstrated by bibliometric data. This is evident from the notable increase in research, particularly on exosomes as delivery vehicles, which has grown at an impressive annual rate of 55.8% since 2013(200). A noteworthy number of patents were generated, and the number of grants sanctioned almost doubled in 2016(199). As the research digs the well of knowledge, the field delves deeper, and there is a vast area left to explore. Earlier, the major focus was on EV-related biofluids and cell types. With the growth, the research started paying pronounced attention to

the role of these **EVs in disease** diagnosis **and** 92

therapy. Current research focuses on the exploration of disease through advanced technologies such as multi-omics analysis with the assistance of bioinformatics analysis, thus providing accurate resolutions. Diagnostically, the EV research focus in the future would be prominently on two aspects. Firstly, there is a requirement for the development of an EV-based rapid disease detection kit that can isolate and characterize organ-specific EVs from circulating biofluids to ascertain the presence of disease in a specific site of the body. Secondly, need for a device that can accurately characterize the organ-specific EVs through multi-omics approaches, providing detailed insights into the incidence of the disease by analyzing risk factors, pathogenesis, progression of the disease, the severity of the disease along mortality predictions. Therapeutically, the EV research field is concerned with the development of EVs as efficient therapeutic agents and cargo carriers. Research focus soon would be on understanding the mechanism of the components of EVs that are effectively improving the disease, which can be implemented for precisely improving the efficiency of EVs as therapeutic agents. The large- scale manufacturing of therapeutic EVs involving selectively enriching the effector EV subpopulation would be the immediate aim of EV therapeutics. Furthermore, EV-based research towards standardized platform development that can accurately deliver the cargo of therapeutic importance to the targeted site more effectively and efficiently, improving the disease condition is required to enhance and extend the targeted delivery utilizing EVs. Synergistic approaches to solve or select targeting moiety utilizing omics and bioinformatics and efficient engineering techniques for expressing the targeting moiety along with EV cargo loading strategy would efficiently pave the way for EVs as therapeutic carriers towards clinics. From finding out the existence of this lipid-layered nanoparticle, and utilizing EVs as next- generation drug carriers, the paradigm is shifting toward translational applications in EV research. Among several lifestyle disorders, MASLD is one such silent disease prevailing worldwide due to metabolic syndrome in most of the population. As prominent particles of intercellular communication, through the development of EV-based devices, they can make one of the minimally invasive methods much more efficient in conveying the mechanism of disease progression along with the severity and efficient therapeutic utility. As the trend in EV research is investigated, they can become “EV” erything for MASLD.

sources:

1 18 words / < 1% match - Internet from 08-Dec-2022 12:00AM
www.frontiersin.org

2 12 words / < 1% match - Internet from 14-Oct-2022 12:00AM
www.frontiersin.org

3 11 words / < 1% match - Internet from 19-Sep-2022 12:00AM
www.frontiersin.org

4 11 words / < 1% match - from 31-Jul-2024 12:00AM
www.frontiersin.org

5 10 words / < 1% match - from 15-Jul-2024 12:00AM
www.frontiersin.org

6 9 words / < 1% match - from 19-Mar-2024 12:00AM
www.frontiersin.org

7 8 words / < 1% match - Internet from 10-Jan-2022 12:00AM
www.frontiersin.org

8 8 words / < 1% match - from 23-Mar-2024 12:00AM
www.frontiersin.org

9 61 words / < 1% match - Internet
Nicolas Cheuk Hang Lau, Judy Wai Ping Yam. "From Exosome Biogenesis to Absorption: Key Takeaways for Cancer Research", Cancers

10 8 words / < 1% match - Internet
Charis Ringland, Jonas Elias Schweig, Maxwell Eisenbaum, Daniel Paris et al. "MMP9 modulation improves specific neurobehavioral deficits in a mouse model of Alzheimer's disease", BMC Neuroscience

11 8 words / < 1% match - Internet
JosefineEilsø Nielsen, Reidar Lund. "Molecular Transport and Growth of Lipid Vesicles Exposedto Antimicrobial Peptides", Langmuir

12 37 words / < 1% match - from 30-Apr-2023 12:00AM
www.mdpi.com

13 17 words / < 1% match - from 26-Aug-2024 12:00AM
www.mdpi.com

14 13 words / < 1% match - from 02-Jun-2023 12:00AM
www.mdpi.com

15 8 words / < 1% match - from 01-May-2024 12:00AM
WWW.MDPI.COM

16 69 words / < 1% match - from 25-Apr-2024 12:00AM
mts.intechopen.com

17 30 words / < 1% match - from 23-Mar-2024 12:00AM
eprints.whiterose.ac.uk

18 17 words / < 1% match - from 07-Sep-2024 12:00AM
eprints.whiterose.ac.uk

19 29 words / < 1% match - from 18-Apr-2024 12:00AM
discovery.researcher.life

20 9 words / < 1% match - from 20-May-2024 12:00AM
discovery.researcher.life

21 8 words / < 1% match - from 24-Nov-2023 12:00AM
discovery.researcher.life

22 20 words / < 1% match - from 21-May-2024 12:00AM
www.science.gov

23 16 words / < 1% match - from 01-Jun-2024 12:00AM
www.science.gov

24 8 words / < 1% match - from 08-Jul-2024 12:00AM
www.science.gov

25 43 words / < 1% match - from 08-Jun-2024 12:00AM
docslib.org

26 36 words / < 1% match - Crossref
"Extracellular Vesicles", Springer Science and Business Media LLC, 2017

27 30 words / < 1% match - Crossref
Bahare Niknam, Kaveh Baghaei, Seyed Mahmoud Hashemi, Behzad Hatami, Mohammad Reza Zali, Davar Amani. "Human Wharton's jelly mesenchymal stem cells derived-exosomes enriched by miR-

124 promote an anti-fibrotic response in an experimental model of liver fibrosis", International Immunopharmacology, 2023

---

28 | 21 words / < 1% match - Internet from 08-Sep-2022 12:00AM
www.nature.com

29 | 9 words / < 1% match - from 08-May-2023 12:00AM
www.nature.com

30 | 29 words / < 1% match - Crossref
Rou Tang, Rui Li, He Li, Xiao-Lei Ma, Peng Du, Xiao-You Yu, Ling Ren, Lu-Lu Wang, Wen-Sheng Zheng. "Design of Hepatic Targeted Drug Delivery Systems for Natural Products: Insights into Nomenclature Revision of Nonalcoholic Fatty Liver Disease", ACS Nano, 2021

31 | 29 words / < 1% match - from 16-Dec-2023 12:00AM
journals.sums.ac.ir

32 | 18 words / < 1% match - Internet from 05-Jan-2023 12:00AM
www.researchgate.net

33 | 11 words / < 1% match - Internet from 24-Feb-2023 12:00AM
www.researchgate.net

34 | 28 words / < 1% match - from 17-Nov-2024 12:00AM
www.ajmc.com

35 | 12 words / < 1% match - from 26-Oct-2023 12:00AM
pubmed.ncbi.nlm.nih.gov

36 | 10 words / < 1% match - from 06-May-2024 12:00AM
pubmed.ncbi.nlm.nih.gov

37 | 14 words / < 1% match - from 06-May-2024 12:00AM
www2.mdpi.com

38 | 8 words / < 1% match - from 01-Jun-2024 12:00AM
www2.mdpi.com

39 | 21 words / < 1% match - Publications
Lara Scheherazade Milane, Mansoor M. Amiji. "Organelle and Molecular Targeting", CRC Press, 2021

40 13 words / < 1% match - from 10-Apr-2024 12:00AM
arts.units.it

41 8 words / < 1% match - from 20-Sep-2024 12:00AM
arts.units.it

42 11 words / < 1% match - from 11-Jul-2024 12:00AM
doaj.org

43 9 words / < 1% match - from 21-Jan-2024 12:00AM
doaj.org

44 10 words / < 1% match - Internet from 23-Sep-2022 12:00AM
link.springer.com

45 10 words / < 1% match - Internet from 29-Dec-2019 12:00AM
link.springer.com

46 19 words / < 1% match - Publications
Richard K. Burt, Dominique Farge, Milton A. Ruiz, Riccardo Saccardi, John A. Snowden. "Hematopoietic Stem Cell Transplantation and Cellular Therapies for Autoimmune Diseases", CRC Press, 2021

47 19 words / < 1% match - ProQuest
Stevens, Lisa Michelle. "Understanding the early events in Bluetongue virus cell entry.", University of Surrey (United Kingdom), 2017

48 19 words / < 1% match - Internet
Chiu, Justin K, .. "The Rho GTPases Rac1, Cdc42, and RhoA Regulate APP Transport to Lysosomes and Aβ Production", Scholarship@Western, 2015

49 18 words / < 1% match - from 17-May-2024 12:00AM
air.unimi.it

50 18 words / < 1% match - Internet
Bruno, Stefania, Pasquino, Chiara et al. "HLSC-Derived Extracellular Vesicles Attenuate Liver Fibrosis and Inflammation in a Murine Model of Non-alcoholic Steatohepatitis", 'Elsevier BV', 2019

51 17 words / < 1% match - Internet from 15-May-2018 12:00AM
pure.uva.nl

52 17 words / < 1% match - Internet from 20-Dec-2022 12:00AM
www.halic.edu.tr

53 8 words / < 1% match - from 09-Aug-2024 12:00AM
worldwidescience.org

54 8 words / < 1% match - from 12-Jul-2024 12:00AM
worldwidescience.org

55 15 words / < 1% match - Crossref
Ai-Lei Xu, Long Han, Jun Yan, Dan Liu, Wei Wang. "Effects of Mesenchymal Stem Cells-Derived Extracellular Vesicles on Inhibition of Hepatic Fibrosis by Delivering miR-200a", Tissue Engineering and Regenerative Medicine, 2024

56 15 words / < 1% match - Crossref
Shenmin Xie, Qin Zhang, Li Jiang. "Current Knowledge on Exosome Biogenesis, Cargo-Sorting Mechanism and Therapeutic Implications", Membranes, 2022

57 14 words / < 1% match - Crossref
Huilin Shao, Hyungsoon Im, Cesar M. Castro, Xandra Breakefield, Ralph Weissleder, Hakho Lee. "New Technologies for Analysis of Extracellular Vesicles", Chemical Reviews, 2018

58 14 words / < 1% match - from 02-Nov-2024 12:00AM
f1000research.com

59 14 words / < 1% match - from 03-Jun-2024 12:00AM
mdpi-res.com

60 13 words / < 1% match - Crossref
Samir Hassoun, Chiara Bruckmann, Stefano Ciardullo, Gianluca Perseghin et al. "NAIF: A novel artificial intelligence-based tool for accurate diagnosis of stage F3/F4 liver fibrosis in the general adult population, validated with three external datasets", International Journal of Medical Informatics, 2024

61 13 words / < 1% match - from 07-Nov-2024 12:00AM
www.fortunejournals.com

62 12 words / < 1% match - Crossref
Oyekoya T. Ayonrinde. "Historical narrative from fatty liver in the nineteenth century to contemporary NAFLD – Reconciling the present with the past", JHEP Reports, 2021

63 12 words / < 1% match - from 22-Oct-2023 12:00AM
acervo.ufrn.br

64 12 words / < 1% match - from 09-Mar-2024 12:00AM
dokumen.pub

65 12 words / < 1% match - from 08-Apr-2024 12:00AM
omjournal.org

66 12 words / < 1% match - Internet from 18-Mar-2020 12:00AM
royalsocietypublishing.org

67 12 words / < 1% match - from 02-Oct-2023 12:00AM
www.omicsdi.org

68 11 words / < 1% match - Internet from 16-Oct-2022 12:00AM
search.bvsalud.org

69 11 words / < 1% match - from 27-Oct-2024 12:00AM
www.wjgnet.com

70 10 words / < 1% match - Publications
Carlos Simón, Carmen Rubio. "Handbook of Genetic Diagnostic Technologies in Reproductive Medicine - Improving Patient Success Rates and Infant Health", CRC Press, 2022

71 10 words / < 1% match - Crossref
Gabriel Scarlat, Bassil Dona, Mihai Cârstea, Marilena Stoian. "Insights into Non-Alcoholic Fatty Liver Disease and Non-Alcoholic Steatohepatitis", Internal Medicine, 2022

72 10 words / < 1% match - Crossref
McGlynn, K.A.. "Epidemiology and natural history of hepatocellular carcinoma", Best Practice & Research Clinical Gastroenterology, 200502

73 10 words / < 1% match - Publications
Nayan A. Gujarathi, Raj K. Keservani, Rajesh K. Kesharwani, Bhushan R. Rane, Yogeeta Sameer Goyal. "Antioxidants As Nutraceuticals", Apple Academic Press, 2025

74 10 words / < 1% match - Internet from 23-Jan-2022 12:00AM
molecular-cancer.biomedcentral.com

75 10 words / < 1% match - from 25-Sep-2023 12:00AM
www.emergenresearch.com

76 9 words / < 1% match - Crossref
Alvin Tieu, Manoj M. Lalu, Mitchell Slobodian, Catherine Gnyra et al. "An Analysis of Mesenchymal Stem Cell-Derived Extracellular Vesicles for Preclinical Use", ACS Nano, 2020

77 9 words / < 1% match - Publications
Raj K. Keservani, Sharangouda J. Patil, Ivan Aranha. "Nutraceuticals for the Treatment and Prevention of Sexual Disorders", Apple Academic Press, 2025

78 9 words / < 1% match - Internet from 25-Nov-2022 12:00AM
downloads.hindawi.com

79 9 words / < 1% match - from 26-Apr-2023 12:00AM
iris.unige.it

80 9 words / < 1% match - Internet from 06-Oct-2020 12:00AM
onlinelibrary.wiley.com

81 9 words / < 1% match - Internet from 12-Jul-2020 12:00AM
pesquisa.bvsalud.org

82 9 words / < 1% match - from 21-Sep-2024 12:00AM
www.i-jmr.org

83 8 words / < 1% match - Crossref
Celeste Caruso Bavisotto, Antonella Marino Gammazza, Claudia Campanella, Fabio Bucchieri, Francesco Cappello. "Extracellular heat shock proteins in cancer: From early diagnosis to new therapeutic approach", Seminars in Cancer Biology, 2021

84 8 words / < 1% match - Crossref
George Chigozie Njoku, Cathal Patrick Forkan, Fumie Mitani Soltysik, Peter Lindberg Nejsum, Flemming Pociot, Reza Yarani. "Unleashing the potential of extracellular vesicles for ulcerative colitis and Crohn's disease therapy", Bioactive Materials, 2025

85 8 words / < 1% match - Publications
Peixuan Guo, Kirill A. Afonin. "RNA Nanotechnology and Therapeutics", CRC Press, 2022

86 8 words / < 1% match - ProQuest
Simão, André Daniel Lopes. "Role of Mitochondria-Targeting MiRNAs in Non-Alcoholic Fatty Liver Disease", Universidade de Lisboa (Portugal), 2024

87 8 words / < 1% match - Internet from 01-Oct-2021 12:00AM
academic.oup.com

88 8 words / < 1% match - ProQuest
dos Santos, Carolina Marques. "Can the Extracellular Vesicles Released by Müller Glial Cells Provide Neuroprotection to Retinal Ganglion Cells?", Universidade de Coimbra (Portugal), 2024

89 8 words / < 1% match - from 30-May-2024 12:00AM
encyclopedia.pub

90 8 words / < 1% match - from 28-Nov-2024 12:00AM
pmc.ncbi.nlm.nih.gov

91 7 words / < 1% match - Crossref
Ciro Tetta, Maria Chiara Deregibus, Giovanni Camussi. "Stem cells and stem cell-derived extracellular vesicles in acute and chronic kidney diseases: mechanisms of repair", Annals of Translational Medicine, 2020

92 7 words / < 1% match - ProQuest
Clarke, Emily-Jayne. "Exploring the Role of Extracellular Vesicles in Equine Musculoskeletal Pathologies and Associated Regenerative Therapies", The University of Liverpool (United Kingdom), 2024

93 7 words / < 1% match - Publications
David Sherman, Ronald Ross Watson. "Ethanol and the Liver - Mechanisms and Management", CRC Press, 2019

94 7 words / < 1% match - Crossref
Kettle, Emma, Scott L. Page, Garry P. Morgan, Chandra S. Malladi, Chin L. Wong, Ross A. Boadle, Brad J. Marsh, Phillip J. Robinson, and Megan Chircop. "A cholesterol-dependent endocytic mechanism generates midbody tubules during cytokinesis : Endocytic midbody tubules linked with cytokinesis", Traffic, 2015.

95 7 words / < 1% match - Publications
Manzoor Ahmad Mir. "p53 in Breast Cancer - Molecular Mechanisms, Clinical Implications, and Therapeutic Targets", CRC Press, 2024

96 7 words / < 1% match - Crossref
Nicolas Cheuk Hang Lau, Judy Wai Ping Yam. "From Exosome Biogenesis to Absorption: Key Takeaways for Cancer Research", Cancers, 2023

97 7 words / < 1% match - ProQuest
Pandit, Rachana. "Canine Coronavirus Infection Alters the Cell Derived-Extracellular Vesicles Biogenesis and Pharmacological-Mediated Cellular Activity", Alabama State University, 2024

98 7 words / < 1% match - Crossref
Tayyaba Saleem, Aleena Sumrin, Muhammad Bilal, Hamid Bashir, Muhammad Babar Khawar. "Tumor-derived extracellular vesicles: Potential tool for cancer prognosis, diagnosis, and therapy", Saudi Journal of Biological Sciences, 2022
